# Targeted therapy for head and neck cancer: signaling pathways and clinical studies

**DOI:** 10.1038/s41392-022-01297-0

**Published:** 2023-01-16

**Authors:** Qingfang Li, Yan Tie, Aqu Alu, Xuelei Ma, Huashan Shi

**Affiliations:** Department of Biotherapy, Cancer Center, West China Hospital, Sichuan University, Chengdu, China

**Keywords:** Head and neck cancer, Head and neck cancer, Cancer therapy

## Abstract

Head and neck cancer (HNC) is malignant, genetically complex and difficult to treat and is the sixth most frequent cancer, with tobacco, alcohol and human papillomavirus being major risk factors. Based on epigenetic data, HNC is remarkably heterogeneous, and treatment remains challenging. There is a lack of significant improvement in survival and quality of life in patients with HNC. Over half of HNC patients experience locoregional recurrence or distal metastasis despite the current multiple traditional therapeutic strategies and immunotherapy. In addition, resistance to chemotherapy, radiotherapy and some targeted therapies is common. Therefore, it is urgent to explore more effective and tolerable targeted therapies to improve the clinical outcomes of HNC patients. Recent targeted therapy studies have focused on identifying promising biomarkers and developing more effective targeted therapies. A well understanding of the pathogenesis of HNC contributes to learning more about its inner association, which provides novel insight into the development of small molecule inhibitors. In this review, we summarized the vital signaling pathways and discussed the current potential therapeutic targets against critical molecules in HNC, as well as presenting preclinical animal models and ongoing or completed clinical studies about targeted therapy, which may contribute to a more favorable prognosis of HNC. Targeted therapy in combination with other therapies and its limitations were also discussed.

## Introduction

Head and neck cancer (HNC) is the sixth most frequent cancer type worldwide, with over 870,000 new cases and 440,000 deaths in 2020.^1^ Head and neck squamous cell carcinoma (HNSCC) is the most common type of HNC, accounting for approximately 90% of HNC cases, and primarily originates from the mucosal epithelium of the oral cavity, pharynx and larynx.^2^ The incidence of HNSCC is increasing and is predicted to rise to 1.08 million new cases per year by 2030.^3^ Exposure to tobacco-derived carcinogens and chronic heavy alcohol consumption are high-risk factors for HNC globally. Recently, increasing tumors in the oropharynx have been associated with previous infection with human papillomavirus (HPV, mainly HPV-16 and HPV-18), especially in Western countries.^4^ As the most common oncogenic HPVs, HPV-16 and HPV-18 infection can be prevented by commercialized HPV vaccines. Therefore, it is feasible to prevent HPV-positive HNSCC by mass vaccination worldwide, just as it is to prevent cervical cancer. Cancers that arise in the oral cavity and larynx are mostly related to smoking and are classified as HPV-negative HNSCC.^5^ Epstein‒Barr virus infection is another risk factor that can contribute to the carcinogenesis of nasopharyngeal carcinoma (NPC).^6^

There are no effective screening strategies for HNC, and most patients are often diagnosed at late stages. HNC is remarkably heterogeneous, and its treatment remains a challenge. Treatment of HNC patients requires aggressive multimodality approaches, including surgery followed by radiotherapy alone or with chemotherapy (known as chemoradiotherapy or chemoradiation) for oral cavity cancers and primary chemoradiotherapy for pharynx and larynx cancers. HPV-positive HNSCC usually displays a more favorable clinical outcome than HPV-negative HNSCC, resulting in the adaptation in the eighth edition of the tumor–node–metastasis (TNM) staging to include p16INK4A immunostaining to indicate HPV status.^2^ Recently, two immune checkpoint inhibitors, pembrolizumab and nivolumab, have been approved by the Food and Drug Administration (FDA) for the treatment of recurrent or metastatic HNSCC (R/M-HNSCC), and pembrolizumab is a first-line therapy for unresectable tumors.^7–9^ However, the prognosis remains poor. There is a lack of significant improvement in survival, and over half of HNSCC patients experience locoregional recurrence or distal metastasis.^5,10^ In addition, patients receiving chemoradiotherapy may exhibit serious side effects and a poor quality of life.^11–14^ Therefore, there is an urgent need to explore more effective and tolerable strategies to improve the clinical outcomes of HNC patients.

**Fig. 1:**
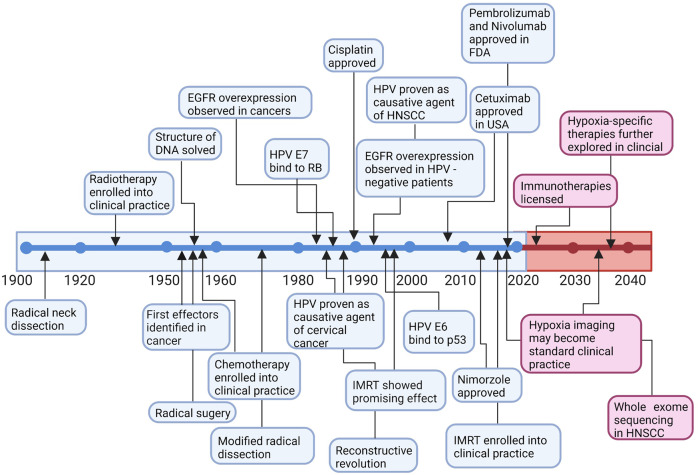
Timeline of treatment regimens and targeted therapy development in head and neck cancer and further investigation. Early on, surgery was first used to treat head and neck cancer. With further investigation, more therapies have been used to treat head and neck cancer. In the 1980s, an advanced understanding of HNSCC was made, and some major discoveries were made between the 1980s and 2020s. The prognosis of head and neck cancers was slightly improved but unsatisfactory. Some discoveries might first be made in cancers other than head and neck cancers, and the same discoveries were identified in head and neck cancers after years. More agents were found and approved in head and neck cancers, which may dramatically improve the prognosis of head and neck cancer patients. DNA deoxyribonucleic acid, EGFR epidermal growth factor receptor, HPV human papillomavirus, HNSCC head and neck squamous cell carcinoma, FDA Food and Drug Administration, IMRT intensity modulated radiation therapy

In recent decades, great success has been achieved in targeted therapy of HNC, which can accurately identify and kill cancer cells with low toxicity and side effects (Fig. 1). In 1984, Hendler et al. discovered a 2.5- to 5-fold increase in the expression of epidermal growth factor receptor (EGFR, HER1 or ErbB1) in human squamous cell lung cancers and epidermoid head and neck cancers.^15^ EGFR belongs to the HER/ErbB family (consisting of HER/EGFR/ErbB 1 to 4) of receptor tyrosine kinases (RTKs), the activation of which leads to proliferation and metastasis of malignant cells and increased angiogenesis.^16–19^ Early in 2001, the efficacy of cetuximab was investigated in squamous cell carcinomas in vivo, which also enhanced the efficacy of radiotherapy.^20–22^ Shortly thereafter, cetuximab showed dose-dependent pharmacokinetics, tolerability, and biologic activity when combined with cisplatin in patients with advanced tumors overexpressing EGFR.^23^ Further studies indicated that cetuximab is an effective radiation sensitizer,^24,25^ resulting in the FDA approval of cetuximab plus radiotherapy for the treatment of locally advanced HNSCC (LA-HNSCC) patients in 2006. The addition of cetuximab to platinum-based chemotherapy increased the median overall survival (OS) to 10.1 months, which makes the EXTREME protocol (cetuximab, cisplatin, or carboplatin, 5-fluorouracil) the standard first-line treatment for R/M-HNSCC patients.^26^ Although cetuximab increased the clinical efficacy and safety of conventional chemotherapy/radiotherapy, there is still much room for improvement in clinical outcomes and quality of life.^27^ Current efforts are focusing on developing more potent and safe agents targeting EGFR and other signaling pathways, including vascular endothelial growth factor receptor (VEGFR), signaling, phosphatidylinositol 3-kinase (PI3K) signaling, and hepatocyte growth factor receptor (c-MET) signaling pathways.

In this review, we summarized the vital signaling pathways and discussed the current potential therapeutic targets in HNSCC as well as presenting preclinical animal models and ongoing or completed clinical studies about targeted therapy, which may contribute to a more favorable prognosis of HNSCC.

## The genetic landscape in head and neck cancer

### EGFR pathway

EGFR is a transmembrane glycoprotein and a cell surface receptor and is the primary member of the HER/ErbB family responding to RTKs. In HNC, EGFR mutation and amplification are rare. However, EGFR is overexpressed in ~80% of HNSCC cases and is closely related to poor prognosis.^28^ EGFR binds with HER family ligands involving epidermal growth factor (EGF), heparin binding-EGF, amphiregulin, transforming growth factor-alpha (TGF-α), epiregulin, and betacellulin, leading to a signal transduction cascade thereby promoting tumor proliferation, invasion, angiogenesis and metastasis and determining the outcomes of diseases.^29^ Approaches to activate EGFR are multitudinous in head and neck cancer. The autocrine or paracrine effects of EGFR ligands, increasing the production of amphiregulin and TGF-α in response to tobacco smoke, activating G-protein-coupled receptors (GPCRs) and increasing the GPCR ligand PGE2 are involved in EGFR activation.^30,31^ The EGFR pathway is complicated and multidimensional. The binding between EGFR and its ligand leads to homodimerization or heterodimerization with HER2, HER3, or other RTKs, such as insulin-like growth factor (IGF)-1R or MET.^32^ The downstream signal transduction cascades, including the JAK/STAT, PI3K/AKT, MAPK, PLCγ/PKC, and Src pathways, and the crosstalk among these signals are potential and attractive targets for HNC.^30^

**Fig. 2:**
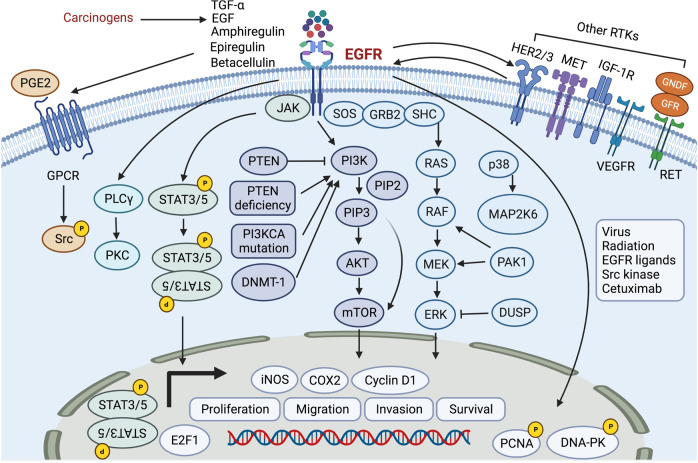
The EGFR signaling pathway, PI3K/AKT/mTOR pathway, MAPK pathway, STAT pathway, and MET pathway in head and neck cancer. EGFR epidermal growth factor receptor, TGF-α transforming growth factor-alpha, EGF epidermal growth factor, RTKs receptor tyrosine kinases, HER2/3 human epidermal growth factor receptor 2/3, c-MET c‑mesenchymal–epithelial transition factor, IGF-1R insulin-like growth factor 1 receptor, PGE2 prostaglandin E2, GPCR G-protein-coupled receptor, PLCγ phospholipase c-γ, PKC protein kinase C, JAK Jenus-activated kinase; STAT3/5, signal transducer and activator of transcription 3/5, PTEN phosphatase and tensin homolog, PI3K phosphoinositide 3-kinase, PIP2 phosphatidylinositol 4,5-bisphosphate, PIP3 phosphatidylinositol 3,4,5-trisphosphate, AKT serine/threonine-specific protein kinase, mTOR mammalian target of rapamycin, DNMT-1 DNA methyltransferase 1, SOS son of sevenless, GRB2 growth factor receptor-bound protein 2, SHC SRC homology domain c-terminal adaptor homolog, ERK: extracellular signal-regulated kinase, MAP2K6 mitogen-activated protein kinase–kinase 6, PAK1 p21-activated kinase 1, DUSP dual-specificity phosphatases, PCNA proliferation cell nuclear antigen, DNA-PK DNA-dependent protein kinase

In addition to the membrane-bound form, EGFR is supposed to translocate to the cell nucleus and play multiple roles. EGFR can return to the cell membrane surface and undergo signal transduction and function. Nuclear EGFR is also associated with lysosome degradation, leading to downstream signal activation.^33^ Moreover, nuclear EGFR can serve as a transcription factor, binding to several gene promotors (cyclo-oxygenase 2 (COX2), inducible nitric oxide synthase (iNOS) and cyclin D1) and DNA-binding transcription cofactors (signal transducer and activator of transcription (STAT3/5) and E2F1), along with PCNA and DNA-PK phosphorylation.^34^ EGFR heterointeraction with Axl has been demonstrated to enhance oncogenic and invasive potential.^35^ The nuclear translocation of EGFR can be triggered by EB virus (EBV), radiation, EGFR ligands, Src family kinase, and cetuximab and is related to poor prognosis and therapeutic resistance.^30,36^ EGFR signaling transduction and crosstalk with other signaling pathways in HNC are shown in Fig. 2.

### PI3K/AKT/mTOR pathway

The PI3K/AKT/mTOR pathway is the most deregulated cancer-driving signaling pathway in HNC and is active in more than 90% of HNSCC cases.^37,38^ The PI3KCA gene is mutated in 16–25% of HNC cases, as previously reported.^39,40^ The mutation frequency of the PI3KCA gene in HPV-positive and HPV-negative HNSCC differs significantly. The chance of HPV-positive HNSCC harboring mutations in the PI3KCA gene is much higher than that of HPV-negative cancer, which makes the PI3K/AKT/mTOR pathway a potential target for HPV-positive disease.^41–43^ PI3Ks are enzyme clusters that are essential for tumor cell growth and differentiation and are activated by RTKs, including EGFR.^44,45^ PI3K is supposed to phosphorylate phosphatidylinositol 4,5-bisphosphate (PIP2) and convert it into phosphatidylinositol 3,4,5-trisphosphate (PIP3).^46^ PIP3 can be dephosphorylated by phosphatase and tensin homolog (PTEN), which in turn blocks the PI3K/AKT/mTOR pathway^47,48^ (Fig. 2).

Mammalian target of rapamycin (mTOR) is a serine/threonine kinase that is essential for tumor growth and proliferation in response to PI3K/AKT signaling in HNC.^49,50^ The mTOR complexes are composed of mTORC1 and mTORC2. The latter is vital for AKT phosphorylation and the downstream signal SGK1 activation.^51,52^ In addition to EGFR activation, the PI3K/AKT/mTOR pathway can be activated through several mechanisms. The PIK3CA gene encodes PI3K, the mutations and amplifications of which are supposed to activate the PI3K/AKT pathway.^53,54^ PTEN mutation is rare in HNC, while PTEN deficiency and PTEN gene copy number loss are prevalent and supposed to activate the PI3K/AKT/mTOR pathway.^55–57^

Activation of the PI3K/AKT/mTOR signaling cascade is associated with therapeutic resistance via enhanced DNA repair mechanisms.^58,59^ Moreover, activation is also associated with EBV-encoded latent membrane proteins 1, 2A, and 2B (LMP1, LMP2A, and LMP2B) in NPC.^60,61^ LMP1 restrains tumor necrosis factor-related apoptosis-inducing ligand (TRAIL)-mediated apoptosis by PI3K/AKT/mTOR activation, which is vital in the induction and maintenance of cancer stem cell properties and tumor cell proliferation and invasion in NPC.^62–64^ LMP1 has been demonstrated to upregulate DNA methyltransferase 1 (DNMT-1) expression and activity, promote mitochondrial translocation, epigenetically silence PTEN, and activate AKT signaling in NPC.^65^ Moreover, the activation of the PI3K/AKT/mTOR pathway mediated by LMP2A is closely related to vasculogenic mimicry formation in EBV-associated epithelial cancers.^66^

### MAPK pathway

The mitogen-activated protein kinase (MAPK) signaling pathway is vital in tumor cell proliferation, differentiation, angiogenesis, metastasis and therapeutic resistance.^67–69^ The MAPK pathway consists of RAS (H/K/NRAS), RAF (A-/B-/C-RAF), mitogen-activated protein kinase–kinase (MEK, MEK1/2), extracellular signal-regulated kinases (ERK, ERK1/2), adaptor molecules (GRB2, SHC1/2/3/4), and dual-specificity phosphatases (DUSP3/5/6/7/9), which are specific negative regulators of ERK.^70,71^ Activation of several kinases, including BRAF, KRAS, HRAS, and ERK1/2, has been demonstrated to induce tumorigenesis and invasion.^72^ The crosstalk between the MAPK pathway and other signals (such as ErbB3, PI3K/AKT/mTOR, JAK/STAT) is thought to drive human oncogenesis and promote tumor progression^73–76^ (Fig. 2).

In HNC, MAPK pathway mutations occur in ~18% of patient tumors.^73^ These mutations predominantly occur in BRAF, HRAS, KRAS, and ERK.^77^ The activators and regulators of the MAPK pathway (NF1, fibroblast growth factor receptor 2 (FGFR2), FGFR3, and ErbB3) have also been found.^78^ Moreover, almost half of the MAPK pathway mutations in head and neck cancer are activating or drivers of tumorigenesis.^70^ When growth factors bind to RAS, a signal cascade is activated. ERK1/2 separates from the RAS/RAF/MEK/ERK1/2 complex and phosphorylates multiple kinases and transcription factors, such as transformation specific-1 (ETS-1), activator protein 1 (AP-1), nuclear factor kappa-B (NF-κB), and c-Myc.^79,80^ In the p38/MAPK pathway, mitogen-activated protein kinase–kinase 6 (MAP2K6) overexpression is associated with radiotherapy resistance and unfavorable prognosis in NPC patients.^81^ The protein kinase (PAK1) is supposed to phosphorylate RAF1 at serine 338 and MEK1 at serine 298, leading to MAPK activation.^82^

### JAK/STAT pathway

Upregulation of the Jenus-activated kinase (JAK)/STAT pathway, especially STAT3 and STAT5, is associated with cell proliferation, angiogenesis, tumor immune evasion, therapy resistance and poor prognosis in HNC.^83–86^ STAT3 signaling is an immunosuppressive molecule that assists tumor cell immune escape by increasing the production of cytokines, such as TGF-β1, IL-6, IL-10, and VEGF.^87^ Mutations in the JAK/STAT pathway are rare in head and neck cancer, as reported previously^88^ (Fig. 2).

Several mechanisms are supposed to activate SATA3 signaling, including RTK (EFGR, VEGFR, Src family kinases (SFK), and JAK), TGF-alpha, alpha7 nicotinic receptor, erythropoietin receptor, G-protein-coupled receptors (GPCRs), Toll-like receptors (TLRs), and the IL-6 cytokine receptor family.^89–92^ Thereafter, phospho-STAT3 in the cell nucleus promotes the expression of downstream target genes, including cyclin D1, survivin and Bcl-xL, which are involved in tumor cell proliferation, angiogenesis and immune evasion.^85,93,94^ Moreover, STAT3 is related to increased expression of immune checkpoints, including PD-L1 and cytotoxic T lymphocyte-associated antigen 4 (CTLA-4). Combined therapy with checkpoint inhibitors is supposed to decrease the resistance against them.^95^

### HGF/MET pathway

The HGF receptor, the sole ligand of c-MET, is overexpressed in the tumor microenvironment and plays an essential role in tumorigenesis and EGFR inhibitor therapy resistance in HNC.^96^ Mutations in the c-MET gene are rare, while an increase in the MET gene copy number and overexpression of HGF are common in HNC.^97^ The HGF/MET pathway is crucial for tumor cell proliferation, angiogenesis, invasion and metastasis in HNC.^98^ HGF activates c-MET, which promotes cellular morphogenesis, epithelial-mesenchymal transition, and tumor metastasis.^99^ Major downstream adapter proteins and kinases in the HGF/MET pathway consist of growth factor receptor-bound protein 2 (GRB2), GRB2-associated adaptor protein 1 (GAB1), RAS, RAS-related C3 botulinum toxin substrate 1 (RAC1), PI3K, STAT, son of sevenless (SOS), cellular Src kinase, Src homology domain c-terminal adaptor homolog (SHC), SRC homology protein tyrosine phosphatase 2 (SHP2), p21-activated kinase (PAK), and phospholipase c-γ (PLC).^100–102^ Moreover, the activation of HGF/MET signaling has been demonstrated to influence the cancer stem cell traits of HNC^103^ (Fig. 2).

The HGF/MET pathway is supposed to crosstalk with other signaling pathways, including the PI3K/AKT pathway, MEK/ERK pathway, STAT pathway and Wnt pathway, to promote tumor progression.^104,105^ The crosstalk between the HGF/MET pathway and the EGFR pathway and VEGFR pathway contributes to therapeutic resistance.^104^

### p53/retinoblastoma (RB) pathway

**Fig. 3:**
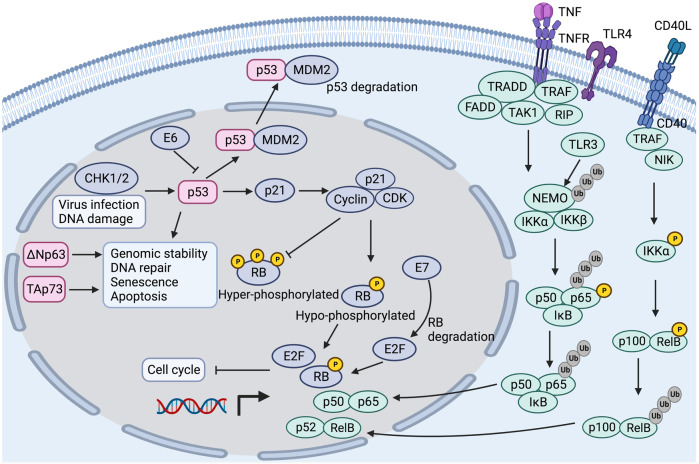
The p53 signaling pathway and NF-κB pathway in head and neck cancer. CHK1/2 cell cycle checkpoint kinase 1/2, MDM2 murine double minute 2, CDK cyclin-dependent kinase, RB retinoblastoma, TNF tumor necrosis factor, TNFR tumor necrosis factor receptor, TLR4 Toll-like receptor 4, TRAF TNFR-associated factor, RIP receptor-interacting protein, IKK IκB kinase

TP53, a tumor suppressor gene, is one of the most prominently mutated genes in HNC and is associated with tumor progression, recurrence, and therapeutic resistance.^106–108^ HPV-positive tumors are associated with p53 degradation, retinoblastoma inactivation, and p16 upregulation, while tobacco-related tumors are more associated with TP53 mutation and downregulation of the p16-encoded gene.^109^ TP53 mutations mostly occur in early tumorigenesis and are related to HPV-negative HNCs because the p53 protein is degraded by the HPV E6 oncoprotein.^110^ TP53 encodes a transcription factor that maintains genomic stability, DNA repair, cell cycle, senescence and apoptosis.^111–113^ P53 is essential for oncogene activation and DNA damage and repair.^114,115^ Murine double minute 2 (MDM2), an E3 ubiquitin-protein ligase, negatively regulates the level of p53.^116^ P53 can be activated by cell cycle checkpoint kinases (such as CHK1 and CHK2), which induce cell apoptosis and cell cycle arrest.^113,117^ The p53 signaling pathway is shown in Fig. 3.

TP63, a p53 family member, is overexpressed in approximately 80% of HNSCC cases.^118,119^ TP63 has two isoforms, TAp63 and ΔNp63. ΔNp63 is the predominant p63 isoform, which is vital for tumorigenesis and progression and senescence suppression and is associated with poor OS in HNC.^120–122^ P63 is considered a stem cell marker.^123^ Moreover, ΔNp63 affects the growth factor signaling pathway and tumor metabolic microenvironment through hyaluronic acid signaling and a transcriptional program.^124^

TP73, another p53 family member, also consists of TAp73 and ΔNp73 isoforms. P73 is related to the tumorigenesis, angiogenesis, and progression of multiple cancers, including HNC.^125–127^ Unlike TP53, the TP73 gene is rarely mutated.^128,129^ The TAp73 isoform affects tumor cell apoptosis and growth arrest instead of the ΔNp73 isoform.^130,131^ The tumor suppressor activity of TAp73 is restrained. The overexpression of TAp73 suppresses the EGFR promoter, downregulates EGFR protein, and induces cell death in HNC cell lines.^130^ More research is needed to illuminate the specific contributions of p73 in cancers.^132^

The RB gene is one of the most important tumor suppressor genes (TP53, RB, PTEN), which regulates the cell division cycle.^133,134^ RB protein binds to EBV protein E7, which leads to protein degradation with E2F release and infinite cell proliferation.^135^ When p53 protein is activated by virus infection or DNA damage, the downstream signal p21 is upregulated, which results in RB-E2F complex (RB, E2F, and dimerization partners) formation and cell cycle gene downregulation. Hence, the p53-p21-RB pathway plays an important role in cell cycle arrest.^133^

### NF-κB pathway

The NF-κB family is a cluster of multipotent dimer transcription factors that are closely related to innate and adaptive immune responses, tumorigenesis, and development.^136–138^ The NF-kB family consists of up to 15 hetero and homodimeric protein complexes drawn from a pool of five monomers.^139,140^ In mammals, the NF-κB family consists of RelA (p65), RelB, RelC, NF-κB1 (p105), NF-κB2 (p100), p50, and p52. Major carcinogens, such as tobacco, alcohol, unhealthy diet, irradiation, and oncogenic viruses, are supposed to activate NF-κB.^141,142^ The activation of NF-κB is associated with EBV infection, the immunosuppressive tumor microenvironment, the maintenance of cancer stem cell characteristics and metabolic reprogramming in nasopharyngeal cancer.^143,144^

The canonical NF-κB pathway depends on cytokine receptors, including interleukin 1 receptor (IL-1R), tumor necrosis factor receptor (TNFR), and Toll-like receptors (TLR4).^145^ The upstream signals of the NF-κB pathway are the receptor-interacting proteins (RIP), TNFR-associated factors (TRAF), the kinase TAK1, the adaptor TRADD and FADD. The NF-κB dimers are combined with inhibitory IκB proteins in the resting state but are activated by IκB kinase (IKK) complex phosphorylation when the stimulus appears. The IKK complex is composed of the active kinases IKKα and IKKβ and the regulatory subunit IKKγ (NEMO). Phosphorylated IκB proteins bind to NF-κB dimers and translocate to the cell nucleus.^146,147^ The noncanonical NF-κB pathway is activated by CD40 ligand and lymphotoxin-β (LT-β) and defined as IKKα-mediated p100 phosphorylation with RelB, resulting in p100 processing and p52-RelB complex generation. The NF-κB downstream target genes can promote cell proliferation, survival, apoptosis, migration, cell cycle control, and angiogenesis^148,149^ (Fig. 3).

The NFKB1 gene rs28362491 polymorphism is significantly associated with HNC, especially NPC, while the NFKBIA gene rs2233406 polymorphism is not.^150^ Moreover, NF-κB is supposed to crosstalk with other signaling pathways, such as the STAT pathway, PI3K/AKT pathway, and p53/RB pathway, to promote tumor prognosis and therapy resistance in multiple cancers, including HNC.^145,151–153^

### Wnt/β-catenin and Notch pathway

The Wnt/β-catenin signal cascade is associated with myriad pathologies in humans, especially in HNC.^154–156^ The Wnt/β-catenin pathway promotes tumor cell proliferation, maintains the stem-like cell phenotype and increases tumor invasiveness in HNC.^157,158^ The Wnt/β-catenin pathway includes extracellular Wnt ligands (Wnt1, 2, 3, 3a), transmembrane receptors, intracellular compounds, β-catenin and transcription factors.^159,160^ The intracellular compounds consist of disheveled (Dvl), degradation complex including glycogen synthase kinase 3 β (GSK-3β), Axin, conductin, casein kinase 1α (CK1α), and adenomatous polyposis coli (APC).^161,162^ Posttranscriptional acylation is vital for extracellular transduction and receptor recognition.^163^ The transmembrane receptors include frizzled receptors (Fzds) and receptor-related protein coreceptors (Lrps).^164^ The Fzds family is composed of more than ten G-protein-coupled receptors, while Lrps comprise Lrp5 and 6, interacting with Fzds for intracellular signal transduction.^165–167^

Wnt combines with cell membrane receptors and controls downstream β-catenin signaling. Activated intracellular β-catenin is transported into the cell nucleus and regulates gene expression as a transcription factor.^168^ Moreover, β-catenin combines with T-cell factor/lymphoid enhancing factor (TCF/LEF) through its armadillo repeats region to form the transcriptional complex, thereby manipulating gene transcription.^169,170^ Epigenetic inactivation of Wnt inhibitory factor 1 (WIF1) and SOX1 is related to aberrant activation of the Wnt/β-catenin pathway and the pathogenesis of HNC.^171,172^

**Fig. 4:**
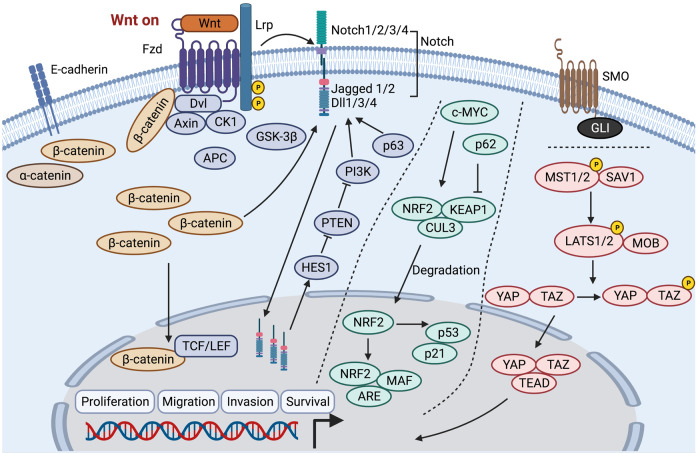
Wnt/β-catenin signaling pathway, Notch pathway, NRF2 pathway, Hippo pathway, and Sonic Hedgehog pathway in head and neck cancer. Fzd frizzled receptor, Lrp receptor-related protein coreceptor, Dvl disheveled, CK1 casein kinase 1, GSK-3β glycogen synthase kinase 3β, APC adenomatous polyposis coli, TCF/LEF T-cell factor/lymphoid enhancing factor, Dll 1/3/4 Delta-like family, HES1 hairy and enhancer of split 1, NRF2, nuclear factor erythroid 2-related factor 2, KEAP1 Kelch-like ECH-associated protein 1, CUL3 cullin-3, ARE antioxidant-responsive elements, YAP yes-associated protein, TAZ transcriptional coactivator with PDZ-binding motif

The Wnt/β-catenin signaling pathway and Notch pathway exhibit close crosstalk with each other to promote tumorigenesis and progression in HNC.^2,173,174^ The Notch pathway has four Notch receptors (Notch1, 2, 3, 4) and five ligands, including the Jagged family (Jagged 1, 2) and the Delta-like family (Dll1, 3, 4).^175,176^ β-catenin reciprocally activates Notch by inducing the expression of Notch signaling ligands (Jagged 1 and Dll1), reduces Notch ubiquitination, increases hairy and enhancer of split 1 (Hes1) expression and affects the downstream signal transduction of the Notch pathway.^159,177^ Wnt/β-catenin signaling transduction and crosstalk with the Notch pathway are shown in Fig. 4.

Notch1 plays a vital role in maintaining cancer stem cell characteristics and increasing tumor recurrence and metastasis.^178,179^ Loss of Notch can promote tumorigenesis by upregulating delta Np63 in HNC. However, delta Np63 expressed in keratinocytes can impair Notch signaling.^180,181^ Notch receptors are overexpressed in HNC samples and activate downstream signal transduction via hairy/enhancer-of-split related with YRPW motif 1 (Hey1).^108,182^ Inactivating mutations in the Notch gene are observed in 17–26% of HNC cases.^108^ Notch1 mutations are predominant, including missense mutations of functional regions, nonsense mutations of truncated proteins, deletions and frameshift insertions.^183^

### Other signaling pathways in HNC

Vascular endothelial growth factor (VEGF), an angiogenesis factor, is highly expressed in HNC and is vital for neovascularization and related to poor prognosis.^184–186^ VEGF (VEGF-A) is a member of the platelet-derived growth factor (PDGF) superfamily. The ligands of VEGF signals include VEGFR1, VEGFR-2, VEGFR-3, and the coreceptor neuropilins (NRP-1, NRP-2).^187,188^ Activated angiogenesis is related to tumor cell proliferation, migration, metastases, and increased sensitivity to radiotherapy and chemotherapy.^189,190^

The gene rearranged during transfection (RET) encodes RET kinase, which is one of the receptor tyrosine protein kinases and is related to the tumorigenesis and progression of thyroid cancer.^191^ RET has three isoforms, including RET9, RET43, and RET51. The ligands of the RET receptor belong to the glial cell line-derived neurotrophic factor (GDNF) family, which includes GDNF, neurturin, artemin, and persephin.^192^ RET receptor binding to its ligands is dependent on a cofactor, which belongs to the growth factor receptor-alpha (GFRα) family, including GFRα1, GFRα2, GFRα3, and GFRα4. GFRα1–4 bind to the RET ligands GDNF, neurturin, artemin, and persephin, respectively, with high affinity and specificity, thereby forming a binary complex and stimulating RET kinase.^193^ The phosphorylation of RET kinase activates multiple downstream pathways, including the MAPK pathway, JAK/STAT pathway, PI3K/AKT/mTOR pathway, and PKC, which are associated with tumor cell proliferation, invasion, migration and survival.^194,195^ Mutations and rearrangements of the RET gene are commonly discovered in thyroid cancer. Mutations of the RET gene are usually observed in papillary and medullary carcinomas, which are key factors in tumorigenesis and progression.^196,197^ Somatic gene rearrangements and fusions of RET occur in ~2.5–73% of papillary thyroid carcinomas, which are the most common subtype of differentiated thyroid cancer. The most common fusions of the RET gene are NCOA4-RET and CDCC6-RET in papillary thyroid carcinoma.^198,199^

Nuclear factor erythroid 2-related factor 2 (NRF2) pathway activation is related to promoting cellular resistance to oxidative stress, proliferation, xenobiotic efflux, metabolic reprogramming, resistance to chemotherapy and radiotherapy.^200,201^ Carcinogens trigger c-MYC-mediated NRF2 activation. Under conditions such as oxidative stress, NRF2 eliminates the Kelch-like ECH-associated protein 1/cullin-3 (KEAP1/CUL3) complex and is translocated into the cell nucleus. In the cell nucleus, NRF2 binds to MAF proteins and antioxidant-responsive elements (AREs) and transactivates downstream targeted genes to promote tumor progression in HNC.^202–204^ Hence, NRF2 upregulation is positively related to the malignant characteristics of HNC.^205^ Moreover, NRF2 has been demonstrated to activate the p53/p21 signaling pathway and influence the cell cycle, whereas p62 can inhibit the NRF2/KEAP1/CUL3 complex.^206,207^ Mutations in the NRF2 pathway are commonly observed in HPV-negative HNC patients, while they are rare in patients with HPV infection^208^ (Fig. 4).

The alterations of the Hippo pathway result in persistent yes-associated protein (YAP) and transcriptional coactivator with PDZ-binding motif (TAZ) activation in HNC, which contribute to tumor progression. The most common alterations are mutations of the FAT1 gene and amplification of the TAZ and YAP1 genes.^209,210^ Multiple cellular signals activate the paralogous kinases MST1/2, which are phosphorylated and promote their heterodimerization with SAV1. MST1/2 kinase can phosphorylate LATS1/2 and the LATS1/2 scaffold protein MOB. Then, LATS1/2 phosphorylates YAP/TAZ, leading to YAP/TAZ cytoplasmic retention or proteolytic decay. Activation of YAP is supposed to promote cell proliferation and maintain cancer stem cell functions, epithelial-to-mesenchymal transition, and chemotherapy resistance.^211,212^ The nuclear translocation of YAP/TAZ can inactivate the Hippo pathway, which stimulates cell proliferation^213^ (Fig. 4).

The overexpression of Sonic Hedgehog pathway proteins, SMO and GLI, has been demonstrated to be a prognostic marker and is related to chemotherapeutic resistance and anti-EGFR therapeutic resistance in HNC.^214–217^ Moreover, radiotherapy is supposed to induce GLI1 expression mediated by the mTOR/S6K1 pathway, which in turn causes radioresistance^218^ (Fig. 4).

In addition, Toll-like receptors (TLRs), especially TLR2/3/5, are highly expressed in HNC and are closely related to poor prognosis.^219–221^ Engagement of TLR3 is supposed to activate TRIF and trigger downstream signaling to activate the NF-κB pathway.^222^ TLR2/5 activation is mediated by the c-jun N-terminal kinase-related pathway, PI3K/AKT pathway, and NF-κB pathway.^223^

Overall, multiple signaling pathways are involved in regulating the tumorigenesis and progression of HNC. Among them, the EGFR pathway plays a critical role in HNC and can be activated by EGF, and downstream pathways include the PI3K/AKT, MAPK, JAK/STAT, and other signaling pathways. Moreover, the EGFR pathway can interact with other RTKs, including HER, MET, IGF-1R, VEGFR, and RET, to synergistically regulate the occurrence and development of HNC. TP53 is an important tumor suppressor gene. The p53 family is involved in HNC tumorigenesis and development, regulating the cell cycle, genomic stability, DNA repair, and apoptosis. NRF2 can activate the p53 pathway. In addition, the NF-κB pathway is also important in HNC and is supposed to crosstalk with the STAT, PI3K, and p53 pathways to promote tumor prognosis in HNC. The Notch signaling pathway is involved in HNC radiation resistance and can be activated by PI3K signaling, which can be suppressed by PTEN. Hence, the crosstalk among these signaling pathways is complex in tumorigenesis, progression, and therapy resistance in HNC.

## Targeted therapy in a preclinical model in vivo

Vivo models can be separated into spontaneous, induced, transplantation, and transgenic models. Transplantation models include subcutaneous tumor models, orthotopic tumor models, and patient-derived tumor xenografts (PDXs). In addition to the traditional animal model, novel animal models have been used in developing antitumor drugs in HNC.^224,225^ PDXs are established by patient-derived tumor tissue directly implanted in immunodeficient mice without prior management or planting in wells, which is a new method for evaluating novel therapies.^226^ The construction of a PDX model also provided a novel approach for identifying suitable alterations in tumor tissue to find promising treatment methods.

HPV is the most important oncogenic factor in HNSCC. According to the common evidence of the subtypes of HNSCC, one is HPV-related, and the other is alcohol, tobacco or oral trauma-related: HPV-positive and HPV-negative.^227^ The clinical and biological characteristics are opposite.^228^

As mentioned previously, the PDX model provides a novel approach for identifying suitable alterations in tumor tissue to find promising treatment methods.^229–232^ Recently, this model has been considered an ideal preclinical model for investigating targeted drugs. These PDX models maintained the genetic background in immunodeficient mice. According to the pathology, genes, and cells of tumors, the response in mice can simulate that in humans.^233–235^ The response of tumors with HPV or not brother the response of treatment regimens.^236–239^ The PDX model can reflect the status of HPV. HPV-negative tumors have been shown to be associated with favorable survival.^240–243^ Previous results in PDX models were identified in clinical trials, which showed that they have similar antitumor properties.

PDX models can maintain the structure of tumors and histological characteristics, although it is still debatable how long the microvessel structure from human tissue is maintained.^244,245^ Some studies claimed that prostate tumors derived from humans maintained their microvasculature after transplantation into mice and that the vessel density of the tumor was enhanced after weeks of transplantation.^246,247^ In contrast, a study reported that the vessel density of renal cell carcinoma decreased after transplantation.^246,247^ The difference after tumor implantation in nude mice may explain why some VEGFR inhibitors obtained resistance in the mouse model. Microvasculature variation may also serve as an explanation for the resistance to antiangiogenic drugs. In addition, precise research including one hundred fifty PDX mouse models assessed angiogenic phenotypes to develop a system to select suitable patients to receive targeted therapy, which may help overcome tumor heterogeneity and predict prognosis.^248^

**Fig. 5:**
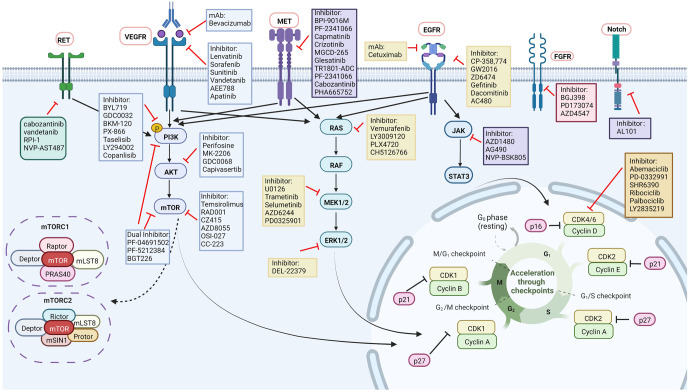
Comprehensive understanding and inhibitor direction for targeting signaling pathways in preclinical HNC treatment. EGFR epidermal growth factor receptor, EGF epidermal augmentum factor, MET mesenchymal–epithelial transition factor, JAK Janus-activated kinase, STAT signal transducer and activator of transcription, AKT serine/threonine-specific protein kinase, mTOR mammalian target of rapamycin, CDK cyclin-dependent kinase, VEGF vascular endothelial growth factor, mAb monoclonal antibody, RET rearranged during transfection, p phosphorylation

A comprehensive understanding and inhibitor direction for targeting signaling pathways in preclinical HNC treatment are shown in Fig. 5.

### EGFR inhibitors

Cetuximab is the only FDA-approved EGFR-targeted therapy in HNSCC.^249–251^ Many compounds targeting EGFR have been developed in preclinical models, with promising response and tolerability profiles. Referencing the current knowledge that multimodality treatment provides new hope for aggressive HNSCC, many studies have investigated the response of combination treatment regimens, either with conventional surgery, radiotherapy and cytotoxic chemotherapy, or in novel precision medicine targeted or immunotherapy combinatorial regimens. In xenograft tumor tissues, EGFR overexpression promoted the radiation therapy response in HPV-positive HNSCC by attenuating DNA damage repair and HPV E6 decrease.^252^

Early in 2001, the effect of cetuximab was investigated in squamous cell carcinoma in vivo, which also enhanced the efficacy of radiotherapy.^253,254^ Xenograft models revealed that EGFR inhibitor monotherapy led to partial and transient tumor regression. The levels of caveolin-1 and Sox-2 in PDX tumors acted as predictive biomarkers of the cetuximab response in HSNCC cells. The accuracy reached 88% according to the cetuximab response.^255^ The combination of cetuximab with either gefitinib (Iressa, and ZD1839, EGFR inhibitor) or erlotinib (Tarceva, and OSI-774, EGFR inhibitor) was investigated in HNSCC. More profound tumor regression and regrowth delay were observed in an in vivo model administered the combination of cetuximab and gefitinib or erlotinib.^256^ The combination of cetuximab with other treatment regimens was further investigated in HNSCC models.

CP-358,774 is a novel potential and selective inhibitor of EGFR and inhibits EGF-mediated mitogenesis in cancer cells. CP-358,774 in combination with cisplatin demonstrated a better response than cisplatin monotherapy with no detectable decrease in body weight or adverse effects.^257^ GW2016 is an effective inhibitor of the ErbB-2 and EGFR tyrosine kinase domains that is undergoing development. ZD6474 is a dual inhibitor of VEGFR-2 and EGFR tyrosine kinase. The efficacy of ZD6474 in HNSCC xenografts was detected. In vivo, ZD6474 inhibited tumor growth via apoptosis and antiangiogenic activity.^258^ Vandetanib (Zactima) is another kind of inhibitor of VEGFR-2, RET, and EGFR. Adenoid cystic carcinoma accounts for 1% of all HNC cases and can be controlled by surgery combined with radiotherapy. No efficient therapeutic compounds have been developed. In the parotid glands of a mouse model, vandetanib was well tolerated and inhibited the mean tumor volumes.^259^

Gefitinib is a selected EGFR inhibitor that inhibits signal transduction to attenuate malignant neoplasm cell growth and proliferation. Gefitinib has demonstrated potential therapeutic activity in HNSCC in vivo by blocking basal EGFR-mediated mitogenic signaling.^260^ Combination therapy with paclitaxel and EGFR inhibitors improved prognosis in a mouse model of oral cancer by promoting cell apoptosis. VEGF inhibitors combined with EGFR inhibitors and radiotherapy resulted in a significant response in the HNC orthotopic model.^261^

The response to combination therapy regimens with EGFR inhibitors in HNSCC was remarkable, while the clinical efficacy of treatment with EGFR inhibitors remained insufficient. In addition to an insufficient response rate, nearly all patients with clinical response finally developed resistance status after approximately ten months of EGFR inhibitor treatment, which indicated that during EGFR inhibitor treatment, some alterations led to the occurrence of resistance. In HN4 and HN6 cell xenograft models, the nerve growth factor (NGF)-TrkA axis induced epithelial-mesenchymal transition (EMT) and mediated resistance to EGFR inhibitors.^262,263^

Dacomitinib (EGFR, ErbB-2, and ErbB4, irreversible inhibitor) was efficient in inhibiting the tumor volume of HNSCC and acted as a radiosensitizing agent in HNSCC. Dacomitinib treatment enhanced the effect of radiotherapy.^264^ AC480 is a novel pan-HER (EGFR and HER2) inhibitor that alone cannot promote tumor cell apoptosis. However, combination with radiotherapy could enhance its effect. The tumor size of HNSCC xenografts in vivo was significantly reduced after AC480 plus radiation.^265^

### VEGF inhibitors

Bevacizumab, sorafenib, and sunitinib are the most common VEGF inhibitors used in targeted therapy.^266^ Bevacizumab is a monoclonal antibody that inhibits angiogenesis in tumors.^267^ The antitumor effect of bevacizumab has been investigated in preclinical tumor models. Bevacizumab successfully inhibited HNSCC growth and had a promising effect on inhibiting HNSCC. Extracellular matrix metalloprotease inducer (EMMPRIN and CD147) is a membrane-bound glycoprotein observed on the membrane of cancer cells.^268^ CD147 overexpression was shown in distinct types of tumors, including HNSCC, hepatocellular carcinoma, gastric cancer, thyroid carcinoma, cervical adenocarcinomas, bladder cancer, and ovarian cancer.^269,270^ The overexpression of CD147 in HNSCC xenografts promoted tumor growth and facilitated the production of VEGF.^271^ CD147 also acted as a predictive effector in predicting the response to bevacizumab in an HNSCC xenograft model.^272^ Although bevacizumab could not inhibit the proliferation of HNSCC cell lines, bevacizumab demonstrated dramatic anticancer efficiency in an HNSCC xenograft model. Bevacizumab plus paclitaxel led to a remarkable effect in HNSCC tumors compared with the single compound effect.^273^ The HNSCC xenograft model benefited from bevacizumab in combination with cisplatin. However, cetuximab treatment alleviated the synergistic effect of bevacizumab and cisplatin.^274^ Cetuximab treatment in combination with other regimens needs further exploration. In addition to HNSCC, bevacizumab had a potential effect in a mouse model of medullary thyroid carcinoma (MTC) and anaplastic thyroid carcinoma. Bevacizumab and EGFR inhibitor monotherapy or in combination blocked angiogenesis in tumors and inhibited tumor growth. Both regimens were superior to doxorubicin treatment.^275^ Pretreatment with bevacizumab promoted radiotherapy in MTC with moderate adverse effects.^276^

Lenvatinib is a novel dual inhibitor targeting VEGFR-2 and FGFR1 that demonstrates robust anticancer efficiency in PDXs and humanized mice, which is equivalent to bevacizumab.^277,278^ AEE788 is a specific kinase inhibitor targeting VEGFR and has a potential effect on EGFR. AEE788 alone and in combination with paclitaxel significantly inhibited the proliferation of tumors in the tongues of athymic nude mice.^279,280^

Apatinib is a novel selective VEGFR-2 inhibitor that inhibits the proliferative ability of thyroid carcinoma and squamous cell carcinoma in vivo and vitro and has been identified in multiple studies.^281,282^ In a tumor xenograft model, mice benefited from either apatinib alone or apatinib in combination with cytotoxic drugs.^283^ Tumor angiogenesis was also suppressed after apatinib treatment. The outcomes demonstrated that apatinib inhibited cancer growth compared with the untreated model group. Apatinib combined with radiation therapy demonstrated a stronger effect in inhibiting tumors than either apatinib treatment alone or radiation therapy alone. The tumor partial oxygen pressure, VEGFR-2-positive cells, and CD31-positive cells in the combination group were lower than those in the single regimen-treated group, which demonstrated that angiogenesis in tumors was blocked.^283^ SCH772984 is an ERK inhibitor and has no effect on tumor growth. Apatinib combined with SCH772984 exhibited a greater inhibitory effect on tumor growth of oral squamous cell carcinoma in vivo, which revealed that inhibition of ERK promoted the anticancer efficiency of apatinib in vivo.^284^ VEGF inhibitors, including ONC201, cabozantinib (XL-184), linifanib (ABT-869), sunitinib, motesanib, pazopanib, axitinib (AG-013736), and PTK787/ZK 222584, all had promising effects in the HNC model.^285–289^

### FGFR inhibitors

BGJ398 is a pan-FGFR inhibitor administered orally that mainly targets FGFR1-3. The effect of BGJ398 in HNSCC was closely related to the expression of FGFR in vivo.^290^ Furthermore, the selective FGFR inhibitor PD173074 alone also resulted in a remarkable response in an HNSCC xenograft model.^291^ AZD4547 is another novel inhibitor with a potent ability to inhibit FGFR1, FGFR2 and FGFR3, which could promote the effect of radiotherapy in an HNSCC patient-derived xenograft model.^292^

### PI3K/Akt/mTOR pathway inhibition

#### PI3K inhibitors

PI3K inhibitors alone do not exhibit a remarkable efficacy like an EGFR tyrosine kinase inhibitor and only selected tumor benefits from them. Human HNSCC xenografts with PIK3CA mutations exhibited susceptibility to therapy with PI3K inhibitors.

BYL719 is a kind of PI3K inhibitor that demonstrated a significant antitumor effect on HNSCC in xenografts. HNSCC patients could benefit from BYL719 inhibitors, and depletion of MYC, p53 mutation, or YAP especially potentiates patients.^293^ Combining BYL719 with KTN3379, a monoclonal antibody targeting HER3, enhanced the suppression of HNSCC in vivo.^294^ However, some HNSCC cells with PI3K-independent activation were resistant to PI3K-independent activation. The AXL-EGFR interaction mediated the process and promoted the antitumor effect of the PI3Kα inhibitor BYL719.^295,296^ Ribociclib is a specific CDK4/6 inhibitor that showed a synergistic effect in combination with BYL719 in nonkeratinizing NPC.^297^ IGF2 inhibitors enhanced the efficacy of BYL719 and taselisib (GDC0032) for the treatment of HPV-positive HNSCC. A PI3K inhibitor also showed promising anticancer effects in cetuximab-resistant oral squamous cell carcinoma.^298^

BKM120, a PI3K inhibitor, inhibited HNSCC cell proliferation in vivo.^299,300^ Among the 353 tested cell lines in mouse xenografts, BKM120 particularly inhibited cancer cells with somatic PI3Kα alternations. BKM120 combined with cetuximab and irradiation significantly inhibited orthotopic xenograft tumors of HNSCC, which provided a rationale for clinical treatment.^301^ Both BKM120 and BYL719 promoted radiosensitive effects in an HNSCC xenograft model. PX-866 also had anticancer efficacy with PIK3CA alterations.^302,303^ Compared with radiation monotherapy, taselisib, a PI3K inhibitor, monotherapy improved the inhibition of cancer cell proliferation. Taselisib plus radiation therapy completely inhibited cancer cell growth, while no significant difference between gross tumor volume was observed at the beginning and end of therapy after three months.^58,304^ LY294002 and copanlisib (BAY 80-6946) were also developed in cancer cell xenograft models.^305–307^ Moreover, patients who responded to PI3K inhibitors acquired drug resistance over time. Few studies have observed acquired resistance to PI3K inhibitors in HNSCC. In HNSCC PDXs that develop resistance to PI3K inhibitors, MAPK activation was detected in the tissue. Inhibiting MAPK activity could resensitize drug-resistant cells to PI3K inhibitors.^308^

#### AKT inhibitors

AKT overexpression in malignant tumor cells demonstrated a stronger response to perifosine than AKT-downregulated cells.^309,310^ The response in tumor cells is related to the phosphorylation of AKT. Perifosine, an AKT inhibitor, promoted cell growth, and apoptosis and blocked the cell cycle, which provided considerable insight into tumor treatment.^311,312^ In HNSCC xenografts, perifosine combined with radiotherapy completely inhibited tumor proliferation and prolonged tumor survival and regression via apoptosis. MK-2206, a novel AKT inhibitor, plus cisplatin also showed a synergistic effect in HSNCC cells.^313^ The ATP-competitive inhibitor ipatasertib (GDC0068) demonstrated significant anti-proliferative effects in mouse xenografts, and the effect in PTEN mutation tumors or with PI3K alterations was enhanced. The AKT inhibitor capivasertib (AZD5363) inhibited AKT activation and resensitized saracatinib-resistant HNSCC cells to saracatinib.^314^ Capivasertib in combination with saracatinib inhibited tumor proliferation more efficiently than either agent in xenografts.

#### mTOR inhibitors

mTOR inhibitors have been proven to improve the efficacy of chemotherapy and radiotherapy without increasing adverse effects by preventing lactate production and inhibiting HNSCC cell proliferation.^315^ In an orthotopic xenograft model of HNSCC, temsirolimus, a potent mTOR inhibitor, exhibited effects on tumor growth. Temsirolimus plus an anti-EGFR inhibitor had a synergistic anticancer effect.^316^ Both temsirolimus and RAD001 treatment showed significant tumor shrinkage, and mTOR activation was inhibited in HPV-positive oral and cervical squamous cell carcinoma (SCC) xenografts. Phosphorylation of its downstream effectors pS6 and pAKT (S473) was observed to be inhibited.^37^ RAD001 also prevented tumor growth of cells with HPV-negative TP53 mutation in vivo through autophagy activity.^317^ The novel mTOR inhibitors CZ415, AZD8055, OSI-027 (ASP4876), and CC-223 monotherapy inhibited HNSCC cell growth in vivo.^318–320^ mTOR monotherapy and combination with other treatment regimens all demonstrated promising effects on the HNSCC xenograft model.

PF-04691502 is an ATP-competitive, dual inhibitor of PI3K and mTOR administered orally. PF-04691502 has produced significant radiosensitization in nonmetastatic HNSCC xenografts.^321^ The study demonstrated that radiosensitization in all HNSCC cell lines was identified regardless of p53, and treatment with PF-04691502 downregulated radiosensitization in normal fibroblasts compared with tumor cells. When the PI3K/AKT/mTOR survival pathway was activated by radiotherapy, PF-04691502 facilitated the efficacy of radiotherapy by inhibiting phosphorylation of effectors in the pathway in HNSCC xenografts, similar to other inhibitors in the PI3K/AKT/mTOR pathway.^322^ Antitumor activity was also observed in HNSCC xenografts with alterations in PIK3CA after treatment with the dual PI3K-mTOR inhibitor PF-5212384, and the activity was promoted by the MEK inhibitor PD-0325901.^323^ BGT226 significantly inhibited tumor growth in HNSCC xenografts.^324,325^ The PI3K/Akt/mTOR pathway has been widely developed in HNSCC. Many inhibitors have been investigated in preclinical studies.

### c-MET inhibitors

BPI-9016 M is a c-MET inhibitor that inhibits tumor growth by promoting tumor cell apoptosis and facilitating DNA damage. Treatment with BPI-9016 M promoted the radiosensitization of tumors in vivo. The volume of esophageal squamous cell carcinoma tumor xenografts was significantly reduced in the combination group compared with each regimen alone.^326^ In a xenograft model, the c-MET inhibitor tepotinib also enhanced the effect of radiotherapy. In addition to radiotherapy, the c-MET tyrosine kinase inhibitor PF-2341066 enhanced the effect of cisplatin in HNSCC.^327^ Not all c-MET inhibitors in combination with other regimens had a synergistic effect. Capmatinib (c-MET-specific inhibitor) plus pitavastatin (HMGCR inhibitor) synergistically inhibited tumor growth and served as a novel treatment regimen in oral and esophageal cancer. However, pitavastatin combined with other c-MET inhibitors (crizotinib or MGCD-265) had no synergistic action on tumors in vivo compared with capmatinib plus pitavastatin.^328^ The results showed that pitavastatin and capmatinib were some of the best combination approaches for inhibiting tumors in vivo, which is worth exploring in clinical trials. The multiple AXL/MET/VEGFR inhibitor glesatinib combined with the MEK inhibitor trametinib also exhibited a remarkable effect in inhibiting tumors. Trametinib as a single compound decreased the tumor volume up to 72% compared with 91% in the combination group. The outcomes demonstrated that mice treated with glesatinib in combination with trametinib showed a promising increased magnitude and durability of response.^329^ The novel c-MET inhibitor TR1801‐ADC (cMet‐targeted “third‐generation” ADC), PF-2341066 (selective c-MET inhibitor), cabozantinib (XL-184), and PHA665752 showed promising effects in HNC in vivo.^330–332^

### RAF inhibitors

RAF inhibitors have a vital role in treating thyroid cancer, and the efficiency of RAF inhibitors is associated with BRAF status. Vemurafenib, a BRAF kinase inhibitor, has remarkable effects in thyroid cancer, but drug resistance results in vemurafenib treatment failure. LY3009120 (pan-RAF inhibitor) helped overcome vemurafenib resistance.^333,334^ PLX4720, a highly selective B-Raf (V600E) inhibitor, significantly attenuated tumor aggression in vivo in thyroid cancer with the B-Raf (V600E) mutation, which also contributed to drug resistance.^335,336^ A tyrosine kinase inhibitor (ponatinib) combined with PLX4720 showed amazing synergistic action in xenograft models of B-Raf (V600E) thyroid cancer, which also contributed to overcome PLX4720 resistance.^337^ The c-MET kinase inhibitor PF-04217903 could enhance the effect of the MEK/RAF inhibitor CH5126766 in murine anaplastic thyroid cancers, which provides new help to patients.^338^ B-Raf (V600E) inhibitor also acted as a radiosensitizer in B-Raf (V600E)-mutant thyroid cancer cells.^339^ RAF inhibitors combined with PI3K inhibitors or MEK1/2 inhibitors all exhibited promising effects in thyroid cancer in a mouse model.^340,341^

### MEK and ERK inhibitors

MEK inhibitors, such as U0126, trametinib, selumetinib, AZD6244, and PD-0325901, demonstrated promising effects in thyroid cancer and squamous carcinoma in HNC in a mouse model. The MEK inhibitor trametinib also resensitized saracatinib-cisplatin HNSCC cells to cisplatin in an orthotopic xenograft model. PD-0325901 enhanced the radiosensitization of HNSCC in vivo.^342,343^

The results offer new insight into overcoming chemoresistance in preclinical HNSCC models and contribute to the further use of MEK inhibitors in clinical research.^344^ The MEK inhibitor selumetinib has been approved in advanced differentiated thyroid carcinoma.^345^ However, some tumors develop MEKi resistance, but the mechanism remains unknown. After MEK inhibitor resistance animal models were established successfully, RTK and SHP2 were observed to be active. SHP2 inhibitor combined with AZD6244 significantly inhibited the tumor volumes and weight compared with the control group, which was superior to single-agent treatment. AZD6244 could also enhance the efficiency of the selective RTK inhibitor tipifarnib in HRAS-driven dedifferentiated thyroid cancers in vivo.^346^ In PDX models of thyroid cancer with different KRAS, BRAF, and NRAS mutations, the MEK inhibitor selumetinib combined with the MDM2 inhibitor KRT-232 showed a remarkable effect. Combination treatment prolonged the survival of mice via MAPK signaling pathway blockade.^347^ Although patients could significantly benefit from lenvatinib (E7080, multitarget inhibitor, mostly for VEGFR-2 (KDR)/VEGFR3 (Flt-4)), the adverse effect of lenvatinib was not tolerated in patients with radioiodine-refractory thyroid cancer.^348^ Lenvatinib plus the MEK inhibitor selumetinib (AZD6244) promoted the anticancer effect in mice with anaplastic thyroid cancer. Combining selumetinib with an FGFR3 inhibitor (PD173074) significantly reduced the tumor volumes and weight in HNSCC xenografts.^349^ DEL-22379, a relatively specific ERK inhibitor, showed remarkable anticancer efficiency in BRAF-mutant anaplastic thyroid cancer in vivo and was a candidate target for cancer therapy.^350^

### Notch inhibitors

AL101 (osugacestat) is a potent γ-secretase inhibitor that blocks the activity of all four Notch receptors. In an adenoid cystic carcinoma xenograft model with Notch1 mutations, the tumor volumes and body weight were reduced by AL101, which provided fairly broad therapeutic prospects in adenoid cystic carcinoma.^351^

### JAK/STAT inhibitors

AZD1480 is an inhibitor targeting JAK1/JAK2 that has been investigated in several tumor models. In PDX models from two independent HNSCC patients, treatment with AZD1480 significantly decreased tumor volumes and weight by decreasing pSTAT3 Tyr705 phosphorylation.^86,352^ In accordance with the promising effect of AZD1480 in HNSCC, another JAK2/STAT3 inhibitor, AG490, was tested in a HNSCC transgenic mouse model, which not only inhibited angiogenesis by suppressing the VEGF receptor but also decreased myeloid-derived suppressor cells.^353^ AG490 further suppressed metastasis and tumor cell proliferation in oral squamous cell carcinoma in vivo.^354^ The JAK/STAT signaling pathway was active after radiotherapy, which may contribute to radiotherapy resistance to reduce the antitumor effect. NVP-BSK805, an inhibitor of JAK2 kinase, promoted DNA double-strand breaks to block the cell cycle and suppress DNA repair to enhance the radiosensitizing effect in esophageal squamous cell carcinoma in vivo. The results demonstrated an attractive prospect of NVP-BSK805 in HNSCC.^355^

### CDK4/6 inhibitors

The effect of CDK4/6 inhibitors is a hotpot in HNSCC.^356–360^ Abemaciclib is the first CDK4/6 inhibitor that has been widely investigated. In vivo, abemaciclib inhibited cancer cell growth compared with the model group without inducing tumor recurrence. The combination of mTOR inhibitors with abemaciclib enhances its anticancer effect.^361^ PD-0332991 is a specific inhibitor of CDK4/6 that has demonstrated potent anticancer effects in HNSCC. The metastasis of esophageal squamous carcinoma could also be suppressed by PD-0332991. The mentioned results provide promising insight into the use of PD-0332991 in clinical trials.^362^

SHR6390 is another new inhibitor specifically inhibiting CDK4/6. SHR6390 suppressed tumor cell growth in vitro and suppressed tumor growth in PDX models.^363–365^ This kind of inhibitor suppressed RB phosphorylation and blocked the cell cycle at G1 in vivo. The combination of SHR6390 with paclitaxel or cisplatin had synergistic action in inhibiting tumor growth in vivo.^365^ The expression of cdK4/6 was related to the prognosis of tumors.

Ribociclib (LEE011) is a selective CDK4/6 inhibitor used in aggressive thyroid cancer via oral administration in a PDX tumor model.^366^ Ribociclib promoted the sensitivity of radiation-resistant esophageal cancer cells by inhibiting YAP1 expression. The combination of ribociclib with radiation overcame radiotherapy resistance in HNSCC in vivo.^367^ Ribociclib has a remarkable influence in HPV-negative HNSCC models. However, ribociclib had no significant efficacy in HPV-positive HNSCC models. In patient-derived tumor xenograft models, the response to ribociclib is closely correlated with retinoblastoma protein production.^368^ The combination of cetuximab and ribociclib showed no synergistic effect in the HNSCC model, but the administration of ribociclib promoted sensitivity to CDK4/6 inhibitors in cetuximab-resistant HPV-negative PDX models.^359^

Palbociclib could block the cell cycle at G1 phase by targeting CDK4/6. In Epstein‒Barr virus-positive HNSCC, palbociclib demonstrated promising effects and decreased the Epstein‒Barr virus titer.^369^ In oral squamous cell carcinoma patient-derived xenografts, palbociclib showed limited efficiency. The administration of combination palbociclib with cetuximab was investigated in preclinical studies, but the effect needs further exploration.^370^ CDKN2A/2B mutation in PDX models acted as a predictive biomarker in predicting palbociclib response. PDX of esophageal squamous cell carcinoma with CDKN2A/2B loss was more sensitive to CDK4/6 inhibitors than those with wild-type CDKN2A/2B.^371^ Palbociclib has shown a promising effect in HNSCC, and the adverse effect of palbociclib is well tolerated. Some drugs have synergistic effects in treating HNSCC. To further explore the synergistic effect of palbociclib plus other drugs, etuximab (EGFR inhibitor), PF-04691502 (PI3K inhibitor), and carboplatin were evaluated, but the efficiency of those regimens was not satisfactory. Trametinib, a MEK inhibitor, has shown a remarkable influence in suppressing HNSCC in vivo. The combination of palbociclib and trametinib was developed in Detroit 562 cells in immunodeficient mice. The tumor weight and volume were reduced by the combination therapy compared with either monotherapy. No significant profiles were observed after palbociclib and trametinib combination treatment.^372^ Palbociclib and SAHA combination therapy also exhibited a synergistic effect in suppressing NPC growth.^373^ LY2835219, a selective CDK4/6 inhibitor, inhibited tumor cell proliferation and blocked the cell cycle by suppressing CDK4/6-dependent Ser780 phosphorylation, which showed a potent effect in treating HNSCC. The combination of LY2835219 with a mTOR inhibitor significantly suppressed HNSCC tumor growth in vivo.^361,374^

### RET inhibitors

RPI-1, a novel 2-indolinone RET tyrosine kinase inhibitor, has shown promising results in sporadic papillary thyroid carcinomas with frequent RET alterations in vivo.^375^ The therapeutic effect was quite immediate, and tumor proliferation was efficiently controlled, with the tumor weight reduced to 20% of the control group. RPI-1 also has remarkable efficacy in MTC xenografts by facilitating tumor cell apoptosis and inhibiting angiogenesis.^376^ The results demonstrated that RET oncogene activity is closely related to the maintenance and survival of MEN2A-type MTC, which contributed to the further administration of RPI-1 in treating thyroid cancer with RET alternation.

NVP-AST487 is another novel RET tyrosine kinase inhibitor that can also inhibit KDR, Flt-4, Flt-3, c-Kit, and c-Abl.^377^ The effect of NVP-AST487 was identified in vivo in medullary thyroid cancer with oncogenic RET. The efficiency of NVP-AST487 was observed to be dose-dependent on RET expression. In the mice treated with 50 mg/kg, the tumor volumes, weight and RET production all exhibited a dramatic decrease. Some multiple target inhibitors, vandetanib and XL-184, were reported to target RET in addition to EGF-R2, VEGF-R3, and EGFR.^285,378,379^ As discussed above, vandetanib showed remarkable effects in adenoid cystic carcinoma, and XL-184 also presented promising results and a moderate effect in HNC by targeting other receptors.^380^

### Novel inhibitors

JNK inhibitors, such as SP600125 and AS601245, have been evaluated in preclinical studies in vivo.^381^ The focal adhesion kinase (FAK) inhibitor defactinib also exhibited an excellent effect in HNSCC. In addition, dual inhibitors have been developed and investigated in xenograft models. Bosutinib (SKI-606) is a second-generation tyrosine kinase inhibitor that acts as a dual inhibitor of Src and Abl. Bosutinib demonstrated a remarkable effect in suppressing tumor growth in vivo.^382^ APG-1252-M1, a dual inhibitor of BCL-2/BCL-XL, had a moderate effect in NPC in vivo. The combination of gemcitabine with APG-1252-M1 led to a promising antitumor effect.^383^

## Targeted therapy in clinical

### EGFR inhibitors

#### EGFR inhibition in locally/regionally advanced head and neck cancer (LA-HNC)

Cetuximab is a chimeric mouse-human monoclonal IgG1 antibody against the extracellular domain of EGFR that can inhibit the functions of EGFR and induce cancer cell death via antibody-dependent NK cell-mediated cytotoxicity (ADCC).^384^ In 2001, Robert et al. first reported that cetuximab is well tolerated in combination with radiotherapy in LA-HNC patients.^24^ Later, a multinational randomized phase 3 study demonstrated that the addition of cetuximab to concomitant high-dose radiotherapy significantly improved disease control and prolonged survival (OS: 49.0 vs. 29.3 months; progression-free survival (PFS): 17.1 months vs. 12.4 months) in locally advanced (LA)-HNSCC patients (Table 1).^25^ Except for acneiform rash and infusion reactions, no significant difference was observed in the incidence of other severe adverse effects between groups, including mucositis, nausea, and radiation dermatitis. These promising results allowed the FDA approval of cetuximab in combination with radiotherapy for the treatment of LA-HNSCC in 2006. The 5-year follow-up of this study also supported the superiority of the combination strategy over radiotherapy alone.^385^ Moreover, cetuximab-treated patients who experienced serious rash exhibited better survival than those with no or low-grade rash.^385^ However, this does not necessarily mean that cetuximab plus radiotherapy can be an effective and safe replacement for the standard chemoradiation composed of cisplatin and radiotherapy. Head-to-head comparison studies indicated that cetuximab + radiotherapy is inferior to the standard chemoradiotherapy (cisplatin + radiotherapy) strategy, with reduced treatment compliance, comparable efficacy outcomes, and increased toxicities.^386–388^ In HPV-positive patients, the addition of cetuximab to radiotherapy showed lower local disease control and shorter survival outcomes than the addition of cisplatin.^389,390^ Cetuximab also brings no additional benefits when combined with cisplatin plus radiotherapy.^391,392^ Therefore, cisplatin remained the first-choice radiosensitizer in all eligible patients, especially for those with HPV infection.

**Table 1:** Representative clinical trial results of targeted therapies in HNC

Target	Treatment	Condition	Phase	Enrollment	Median follow-up	Local control or response rate	OS (months)	PFS (months)	NCT number
EGFR	a. RT + cetuximab; b. RT	LA-SCCHN	3	424	54.0 months	3-year local control: 47% vs. 34%	49.0 vs. 29.3	17.1 vs. 12.4	NCT00004227^25^
EGFR	a. RT + cetuximab; b. RT + cisplatin	LA-SCCHN	2	88	19.3 months	2-year local control: 53% vs. 80%	2-year: 68% vs. 78%	2-year MFS: 97% vs. 90%	NCT01216020^378^
EGFR	a. RT + cetuximab; b. RT + cisplatin	LA-SCCHN	3	298	3.2	3-year LRF: 23% vs. 9%	3-year: 78% vs. 88%	3-year EFS: 67% vs. 85%	NCT01969877^380^
EGFR	a. RT + cetuximab; b. RT + cisplatin	HPV (+) OPC	3	334	25.9 months	Locoregional recurrences: 12% vs 3%	2-year: 89·4% vs 97·5%	–	ISRCTN33522080^381^
EGFR	a. RT + cetuximab; b. RT + cisplatin	HPV (+) OPC	3	805	4.5 years	5-year LRF: 17.3% vs. 9.9%	5-year: 77.9% vs. 84.6%	5-year: 67.3% vs. 78.4%	NCT01302834^382^
EGFR	a. TPF/TP + cetuximab ICT; b. TPF/TP ICT	advanced laryngeal/hypopharyngeal cancer	2	180	24 months	–	24-month: 62% vs. 58%	24-month LFS: 47.1.% vs. 46.6%	NCT00508664^389^
EGFR	a. TPF ICT; b. TPC ICT	LA-SCCHN	2	100	400 days	CR: 32.7% vs. 49.0%	400-day: 78.5% vs. 86.1%	400-day: 66.5% vs. 70.0%	EudraCT-No. 2011-005540-99^390^
EGFR	a. Cisplatin + cetuximab; b. cisplatin + placebo	R/M-SCCHN	3	117	31 months	ORR: 26% vs. 10%	9.2 vs. 8.0	4.2 vs. 2.7	^407^
EGFR	a. PF + cetuximab; b. PF	R/M-SCCHN	3	442	18.2 vs. 19.1 months	ORR: 36% vs. 20%	10.1 vs. 7.4	5.6 vs. 3.3	NCT00122460^26^
EGFR	Paclitaxel, carboplatin + cetuximab	R/M-SCCHN	2	47	20 months	ORR: 40%	14.7	5.2	UMIN000010507^412^
EGFR	a. PCF; b. Cetuximab + docetaxel + cisplatin	R/M-SCCHN	2	541	30.2 vs. 34.4 months	ORR: 59% vs. 57%	13.4 vs. 14.5	6.2 vs. 6.0	NCT02268695^414^
EGFR	a. RT + panitumumab; b. RT + cisplatin	LA-SCCHN	2	152	107.5 vs. 123 weeks	2-year local control: 51% vs. 61%	2-year: 63% vs. 71%	2-year: 41% vs. 62%	NCT00547157^393^
EGFR	a. PF + panitumumab; b. PF	R/M-SCCHN	3	657	44 vs. 35 months	Objective response: 36% vs. 25%	11.1 vs. 9.0	5.8 vs. 4.6	NCT00460265^419^
EGFR	a. RT + cisplatin + nimotuzumab; b. RT + cisplatin	LA-SCCHN	3	536	39.13 months	2-year LRC: 67.5% vs. 57.6%	2-year: 63.8% vs. 57.7%	2-year: 61.8% vs. 50.1	CTRI/2014/09/004980^398^
EGFR	PF + nimotuzumab	R/M-NPC	2	35	13.2 months	ORR: 71.4%	16.3	7.0	NCT01616849^422^
EGFR	Gefitinib + fluorouracil + hydroxyurea +RT	LA-SCCHN	3	69	3.5 years	CR: 90%	4-year: 74%	4-year: 72%	^402^
	a. Afatinib; b. methotrexate	R/M-SCCHN	3	483	6.7 months	ORR: 10% vs. 6%	6.8 vs. 6.0	2.6 vs. 1.7	NCT01345682^429^
VEGF(R)	a. Sorafenib; b. placebo	LA- or R/M-thyroid cancer	3	417	16.2 months	ORR: 12.2% vs. 0.5%	NR	10.8 vs. 5.8	NCT00984282^439^
VEGF(R)	sorafenib + PF	R/M-NPC	2	54	19.0 months	ORR: 77.8%	7.2	11.8	^442^
VEGF(R)	a. Lenvatinib; b. placebo	Radioiodine-refractory thyroid cancer	3	392	17.1 months	ORR: 64.8% vs. 1.5%	–	18.3 vs. 3.6	NCT01321554^443^
VEGF(R)	a. Apatinib; b. placebo	LA- or R/M-thyroid cancer	3	92	18.1 months	ORR: 54.3%	29.9	NR	NCT03048877^456^
VEGF(R)	a. Chemotherapy + bevacizumab; b. chemotherapy	R/M-SCCHN	3	403	40 months	ORR: 35.5% vs. 24.5%	12.6 vs. 11.0	6.0 vs. 4.3	NCT00588770^465^
VEGF(R)	RT + PF + bevacizumab	LA-NPC	2	46	2.5 years	–	2-year: 90.9%	2-year: 83.7%	NCT00408694^467^
VEGF(R)+ EGFR	cetuximab + bevacizumab	R/M-SCCHN	2	46	9.7 months	ORR: 16	7.5	2.8	^416^
VEGF(R)+ EGFR	a. RT + cetuximab + pemetrexed + bevacizumab; b. RT + cetuximab + pemetrexed	LA-SCCHN	2	78	32 months	–	2-year: 88%	2-year: 75% vs. 79%	NCT00703976^472^
PI3K	a. Buparlisib; b. Buparlisib + cetuximab	R/M-SCCHN	2	53	–	Disease control rate: 49% vs. 91%	143 vs. 206 days	63 vs. 111 days	NCT01527877^473^
PI3K	a. Buparlisib + paclitaxel; b. placebo + paclitaxel	R/M-SCCHN	2	158	18.1 months	ORR: 39% vs. 11%	10.4 vs. 6.5	4.6 vs. 3.5	NCT01852292^475^
mTOR	Temsirolimus + carboplatin + paclitaxel	R/M-SCCHN	2	39	–	ORR: 41.7%	12.8	5.9	^492^
c-MET	a. Cabozantinib; b. placebo	Progressive MTC	3	330	13.9 months	ORR: 28% vs. 0%	26.6 vs. 21.1	11.2 vs. 4.0	NCT00704730^496,497^
c-MET	a. Cabozantinib; b. placebo	Radioiodine-refractory DTC	3	227	8.9 months	ORR: 15% vs. 0%	6-month: 85% vs. 73%	6-month: 57% vs. 17%	NCT03690388^499^
RET	Selpercatinib	RET-altered thyroid cancers	1–2	162	7.8 months	ORR: 69–79%	–	1-year: 64–92%	NCT03157128^503^
RET	a. Vandetanib; b. placebo	LA- or metastatic MTC	3	331	24 months	ORR: 45% vs. 13%	–	6-month: 83% vs. 63%	NCT00410761^504^

*RT* radiotherapy, *MFS* metastasis-free survival, *HPV* human papillomavirus, *OPC* oropharyngeal cancer, *LRF* locoregional failure and distant metastasis, *EFS* event-free survival, *TPF* taxanes, cisplatin, and 5-FU, *ICT* induction chemotherapy, *LFS* laryngectomy-free survival, *TPC* taxanes, cisplatin, and cetuximab, *RTC* radiotherapy + cetuximab, *PF* cisplatin and 5-FU, *PCF* platinum, cetuximab and 5-FU, *LRC* locoregional control, *R/M-NPC* recurrent/metastatic nasopharyngeal carcinoma, *NR* not reached, *MTC* medullary thyroid cancer, *DTC* differentiated thyroid cancer

Induction chemotherapy (ICT) with taxanes, cisplatin, and 5-FU (TPF) before receiving chemoradiotherapy or radiotherapy resulted in increased tumor responses and reduced failure in local control and distant metastasis in LA-HNSCC.^393,394^ Whether the addition of cetuximab to ICT regimens brings more benefits than drawbacks remains controversial. In several phase 2 clinical trials, adding cetuximab to the TPF regimen resulted in tolerable and long-term control of LA-HNSCC, especially in HPV-negative cases.^395,396^ However, the head-to-head DeLOS-II trial indicated that the addition of cetuximab to the TPF ICT regimen (TPF-C) showed no superiority in survival outcomes over TPF alone.^397^ Nevertheless, cetuximab can be considered an effective and tolerable substitute for 5-FU in this regime, with a comparable overall response rate (ORR) and OS and slightly fewer serious adverse effects.^398^ Cetuximab, paclitaxel, and carboplatin (PCC) is an alternative ICT regimen that is safe and could help induce a strong local response and promising survival.^399,400^

Other monoclonal antibodies of EGFR are still under intensive investigation, including panitumumab, nimotuzumab, zalutumumab, etc. Similar to cetuximab, panitumumab cannot replace cisplatin when combined with radiotherapy for LA-HNSCC according to the results from the CONCERT-2^401^ and HN.6^402^ trials. Panitumumab plus radiotherapy showed no advantage in improving the local control rate, survival time, and quality of life in treated patients compared with cisplatin plus radiotherapy. Meanwhile, adding panitumumab to the standard chemoradiotherapy strategy failed to provide any benefit,^403^ making panitumumab an unsuitable choice for LA-HNSCC patients. The addition of nimotuzumab to radiotherapy with or without cisplatin provided long-term survival benefits for up to five years and improved the complete response rate in LA-HNSCC patients.^404,405^ In a phase 3 clinical trial involving 536 LA-HNSCC patients, nimotuzumab plus cisplatin and radiotherapy significantly improved the locoregional control rate and PFS without negatively impacting the quality of life.^406,407^ Except for a higher incidence of mucositis, other adverse effects of grade 3 or more were similar with or without nimotuzumab.^406^ The promising results strongly supported the addition of nimotuzumab to LA-HNSCC patients who are treated with cisplatin and radiotherapy. Another monoclonal antibody, zalutumumab, extended the survival time from 8.4 to 9.9 weeks in recurrent or metastatic (R/M) HNSCC patients who had failed platinum-based chemotherapy.^408^ Meanwhile, moderate-to-severe skin rash during zalutumumab treatment was related to superior OS, independent of HPV infection and p16 status.^409^

Some small molecular inhibitors of EGFR are also under investigation for the management of HNSCC, including selective inhibitors (e.g., gefitinib, erlotinib) and dual-target inhibitors (e.g., afatinib, lapatinib, and dacomitinib). Gefitinib is an orally administered selective inhibitor of EGFR. When administered fluorouracil, hydroxyurea, and radiotherapy, gefitinib demonstrated a strong complete response rate and favorable survival outcomes (4-year OS: 74%; PFS: 72%) after a median follow-up of 3.5 years in LA-HNSCC patients.^410^ Lapatinib monotherapy also showed evidence of clinical activity with an ORR of 17% in LA-HNSCC patients compared with placebo.^411^ Lapatinib plus chemoradiotherapy is safe and induces a high complete response rate and long median PFS in p16-negative LA-HNSCC patients.^412^ However, the addition of lapatinib to chemoradiotherapy, followed by lapatinib maintenance brought additional toxicity with limited efficacy in patients with high-risk HNSCC after surgery.^413^ Therefore, lapatinib is unsuitable for long-term maintenance treatment.

#### EGFR inhibition in recurrent or metastatic head and neck cancer (R/M-HNC)

Cetuximab monotherapy was well tolerated and induced a response rate of 13% in R/M-HNSCC patients.^414^ Combining cetuximab with cisplatin contributed to a significant improvement in ORR (26% vs. 10%) but failed to prolong survival for R/M-HNSCC patients when compared with cisplatin monotherapy.^415–417^ In the landmark EXTREME study, cetuximab combined with platinum (cisplatin or carboplatin) and fluorouracil (PCF) resulted in improved median PFS (5.6 months vs. 3.3 months), OS (10.1 months vs. 7.4 months), and response rates (36% vs. 20%) compared with chemotherapy alone.^26^ This led to the approval of cetuximab in combination with platinum-based therapy with fluorouracil as the first-line treatment for R/M-HNSCC patients. However, this standard of care regime has some disadvantages in clinical use, including the requirement of hospitalization to ensure proper hydration and continuous infusion of fluorouracil and severe toxicities such as nausea and anorexia. To improve the feasibility and reduce the adverse effects, cetuximab is under investigation for combining different chemotherapeutics in the treatment of R/M-HNSCC. For instance, cetuximab combined with paclitaxel,^418^ docetaxel and cisplatin,^419^ or paclitaxel and platinum showed promising activity and tolerability as first-line treatment in R/M-HNSCC patients.^420,421^ The cetuximab, carboplatin, and paclitaxel (PCC) regimen induced an ORR of 40%, median OS of 14.7 months, and median PFS of 5.2 months, comparable to those reported with PCF treatment.^26,420^ Further studies involving more patients are needed to confirm the efficacy of PCC and compare it with PCF. In the GORTEC 2014-01 TPExtreme phase 2 study, although a combination of cetuximab, docetaxel, and cisplatin (TPEx) showed no significant improvement in survival outcomes in R/M-HNSCC patients when compared with the PCF regimen, it showed a significantly better safety profile, with fewer people experiencing grade 3 or worse adverse effects (81% vs. 93%).^422^ Therefore, the TPEx regime can be a reliable alternative to the PCF regime in the first-line treatment of R/M-HNSCC patients. In addition to chemotherapy, cetuximab combined with the CDK4/6 inhibitor palbociclib,^423^ VEGF monoclonal antibody bevacizumab,^424^ or immunotherapy (pembrolizumab^425^ and nivolumab^426^) also showed promising clinical activity and safety profiles in R/M-HNSCC patients, including platinum-resistant and cetuximab-resistant patients.

In the phase 3 SPECTRUM clinical study, the addition of panitumumab to cisplatin and fluorouracil resulted in longer median PFS (5.8 months vs. 4.6 months) than chemotherapy alone in R/M-HNSCC patients, especially in patients with p16-negative tumors.^427^ Meanwhile, panitumumab and paclitaxel combination as first-line treatment contributed to almost 50% of confirmed ORR and median PFS of 7.5 months in R/M-HNSCC patients, comparable to the previously reported efficacy of cetuximab–paclitaxel or PCF regimes.^26,418,428^ Panitumumab, docetaxel, and cisplatin may have the potential to improve PFS by 1.4 months in R/M-HNSCC patients. However, there is a tendency for a decrease in OS in the panitumumab-containing regimen compared to chemotherapy alone.^429^ Therefore, further studies are necessary to evaluate the advantages and disadvantages of this triplet combination. A combination of nimotuzumab, cisplatin, and fluorouracil also demonstrated promising efficacy, including both overall response, survival outcomes, and tolerability in recurrent metastatic NPC (R/M-NPC) patients.^430^ These results are consistent with a retrospective study evaluating the antitumor activity and toxicity of nimotuzumab in combination with chemotherapy as first-treatment in 203 R/M-NPC patients.^431^

In a phase 3 study performed on 486 patients, gefitinib monotherapy failed to improve the ORR and survival outcomes in R/M-HNSCC patients when compared to methotrexate.^432^ The addition of gefitinib to docetaxel also failed to improve the clinical outcomes of R/M-HNSCC patients with poor prognosis.^433^ Thus, gefitinib may be used with caution in R/M-HNSCC patients. Erlotinib monotherapy is well tolerated and yields prolonged disease stabilization in heavily pretreated R/M-HNSCC patients.^434^ Erlotinib in combination with the chemotherapeutic cisplatin with^435^ or without^436^ docetaxel exhibited favorable antitumor activity and tolerability comparable to historical controls in R/M-HNSCC, supporting further evaluation of these regimens. Dual-target inhibitors, such as afatinib and dacomitinib, are also under clinical evaluation against R/M-HNSCC. As an irreversible blocker of the ErbB family, afatinib monotherapy induced significantly prolonged PFS (2.6 vs. 1.7 months) versus methotrexate as second-line treatment in R/M-HNSCC patients with manageable safety profiles in the phase 3 LUX-Head & Neck 1 study.^437,438^ The most common serious adverse effects were afatinib-related skin rash/acne and diarrhea. Subgroup analysis indicated that median PFS favored afatinib in patients with p16-negative, EGFR-amplified, HER3-low, and PTEN-high tumors. However, in patients with p16-positive disease, afatinib failed to display an advantage in enhancing PFS.^439^ Therefore, it is necessary to examine the p16 status of R/M-HNSCC before giving afatinib treatment to guarantee patients’ benefits. Similar results were obtained from the LUX-Head & Neck 3 study carried out in an Asian population, demonstrating the superiority and feasibility of afatinib over methotrexate as a second-line treatment for R/M-HNSCC patients.^440^ Afatinib may also improve the efficacy of pembrolizumab by promoting antigen presentation machinery in the tumor microenvironment.^441^ Dacomitinib is another paninhibitor of the ErbB family that demonstrated clinical activity and acceptable toxicity in platinum-refractory HNSCC patients.^442,443^ Poziotinib, a dual inhibitor of EGFR and HER2, exhibited clinically meaningful efficacy (ORR: 24%, median PFS: 4.0 months, median OS: 7.6 months) in R/M-HNSCC patients,^444^ which was noninferior to that induced by afatinib monotherapy.^437,438^

### VEGF inhibitors

Sorafenib is an orally active inhibitor of multiple kinases, including BRAF, VEGFR1, and VEGFR-2, which can inhibit cancer cell proliferation and angiogenesis.^445,446^ In the phase 3 DECISION study, sorafenib significantly improved the ORR (12.2% vs. 0.5%) and PFS (10.8 vs. 5.8 months) versus placebo in patients with locally advanced or metastatic, radioactive iodine-refractory, differentiated thyroid cancer.^447^ Most AEs were grade 1 or 2, and no unexpected AEs occurred. Based on the promising results, sorafenib received FDA and EMA approval for the treatment of radioactive iodine-refractory or metastatic differentiated thyroid cancer (DTC). Further analysis of the DECISION study revealed that elevated baseline levels of VEGFR and thyroglobulin and the presence of RAS mutations were associated with poor PFS, whereas BRAF mutations were related to better PFS.^448^ In R/M-NPC patients, sorafenib showed only modest anticancer activity, with an ORR of 3.7% and an OS of 4.2 months.^449^ To improve the efficacy, sorafenib was combined with cisplatin and 5-fluorouracil. This triplet combination strategy is tolerable and highly effective in treating R/M-NPC patients, with the ORR reaching 77.8% and favorable survival outcomes (PFS: 7.2 months, OS: 11.8 months).^450^ Lenvatinib is an oral inhibitor of VEGFR1/2/3, FGFR1, platelet-derived growth factor receptor α (PDGFRα), RET, and c-kit. In the phase 3 SELECT study, lenvatinib yielded a significantly extended PFS (18.3 vs. 3.6 months) and improved response rate (64.8% vs. 1.5%) compared with placebo, including elevated complete response (1.5% vs. 0%), in radioiodine-refractory thyroid cancer patients of any age.^451,452^ This study promoted the approval of lenvatinib in treating patients with progressive DTC that progressed after radioactive iodine therapy. Despite the promising results from DTC, lenvatinib seems to be disappointing in treating anaplastic thyroid cancer.^453,454^

Sunitinib and apatinib are also multitarget inhibitors of VEGFR, PDGFR, and c-kit, which have been approved for the management of gastrointestinal stromal tumors, renal cell carcinoma, pancreatic cancer, and lung cancer.^455–459^ Sunitinib monotherapy only showed modest clinical activity in R/M-HNSCC patients and was associated with a high incidence of hemorrhage.^460,461^ Although sunitinib may be effective in managing thyroid cancer, serious side effects have also been reported, including asthenia/fatigue and mucosal and cutaneous toxicities.^462,463^ Further studies should focus on different combination strategies to improve sunitinib’s efficacy and safety. In the REALITY placebo-controlled phase 3 clinical trial, apatinib monotherapy exhibited significant promising benefits in both enhanced ORR (54.3% vs. 2.2%) and prolonged survival (both PFS and OS) with favorable safety profiles, strongly supporting the application of apatinib in LA- or metastatic radioactive iodine-refractory DTC patients.^464^ Apatinib may also show efficacy in patients with R/M-NPC^465–467^ and locally advanced oral squamous cell carcinoma^468^ alone or in combination. Other multitarge inhibitors, including anlotinib^469–471^ and donafenib,^472^ have shown antitumor activity with favorable safety in thyroid cancer patients in early phase clinical trials. More studies are warranted to confirm these results.

Bevacizumab is a humanized anti-VEGF monoclonal antibody approved for the treatment of various cancer types, including colon cancer, lung cancer, and breast cancer. Although bevacizumab has not been licensed for use in HNC, preclinical and clinical studies have confirmed its effectiveness and tolerability in improving the outcomes of HNC patients. The addition of bevacizumab to platinum-based chemotherapy^473^ or nonplatinum pemetrexed^474^ significantly improved the response rate (30–35.5%) and survival (OS: 11.3–12.6 months) compared to chemotherapy alone in R/M-HNSCC patients. However, it should be noted that bevacizumab treatment may result in a significantly increased rate of serious bleeding. Whether bevacizumab can increase the sensitivity of HNSCC patients to chemoradiotherapy remains controversial. It has been reported that the addition of bevacizumab to standard chemoradiotherapy (radiotherapy + cisplatin + fluorouracil) is effective and highly tolerable in LA-NPC patients with no hemorrhage of grade 3 to 5. The 2-year OS and PFS reached 90.9% and 83.7, respectively, with a median follow-up of 2.5 years.^475^ However, a phase 2 clinical trial evaluating the effect of bevacizumab plus a different chemoradiotherapy regimen (5-fluorouracil, hydroxyurea, and radiotherapy) in intermediate-stage and T4N0 HNC was terminated in advance because of locoregional progression in the bevacizumab arm,^476^ suggesting the potential of bevacizumab to promote tumor progression in this regime. Accumulating studies have also indicated that there is crosstalk between the EGFR and VEGF pathways and upregulation of VEGF-related angiogenesis-mediated resistance to EGFR inhibition.^477^ Consistently, simultaneous inhibition of EGFR (cetuximab or erlotinib) and VEGF pathways (bevacizumab) was reported to be well tolerated and active in R/M-HNSCC patients, with an ORR of 15–16%, PFS of 2.8–4.1 months and OS of 7.1–7.5 months, indicating bevacizumab’s role in improving patients’ sensitivity to EGFR-targeted therapy.^424,478^ Although dual EGFR and VEGF inhibition combined with concurrent chemoradiation is effective in LA-HNSCC patients,^479^ a head-to-head phase 2 study indicated that the addition of bevacizumab failed to bring additional efficacy to cetuximab-containing chemoradiotherapy.^480^ In contrast, the addition of bevacizumab increased the hemorrhagic rate.^480^ Therefore, more studies concerning the effect of bevacizumab on chemoradiotherapy in HNSCC patients are warranted, either alone or in combination with EGFR-targeted agents.

Therefore, the VEGFR pathway is a promising therapeutic target in HNSCC, especially in thyroid cancer. Further studies should focus on minimizing unwanted adverse effects, especially bleeding events, by developing more tolerable agents with biomarker-driven studies.

### Inhibition of the PI3K/AKT/mTOR pathway

#### PI3K inhibitors

PI3K inhibitors can be divided into two different types: ATP-competitive (e.g., alpelisib, buparlisib, copanlisib) and noncompetitive (PX-866) inhibitors. Almost all the inhibitors are panclass I inhibitors except for alpelisib, which is isoform-specific and targets only the p110α of PI3K.^37^ Clinical evaluation of PI3K inhibitors in HNC is mainly in early phase clinical trials. In R/M-HNSCC, the addition of buparlisib to cetuximab is well tolerated and significantly improved the disease control rate (49% vs. 91%) and prolonged survival outcomes (PFS: 63 vs. 111 days; OS: 143 vs. 206 days) compared to buparlisib monotherapy.^481,482^ Similarly, a combination of buparlisib and paclitaxel showed manageable safety profiles and superiority in median PFS (3.5 vs. 4.6 months) in R/M-HNSCC patients who received previous platinum treatment, indicating the potential of this combination as a second-line treatment strategy.^483^ Biomarker analysis of the molecular alterations revealed that TP53 alterations, HPV-negative status, low mutational load, or high infiltration of CD8 T cells are indicators of survival benefit after treatment with buparlisib and paclitaxel.^484^ Based on these promising results, a randomized phase 3 clinical trial is recruiting 483 patients to assess the efficacy and safety of buparlisib plus paclitaxel compared to paclitaxel alone in R/M-HNSCC patients (NCT04338399). Two phase 1 studies indicated that alpelisib is effective and has a manageable safety profile at dosages of 200 mg-250 mg when combined with cisplatin-based chemoradiotherapy or cetuximab plus radiotherapy in LA-HNSCC patients.^485,486^ Further clinical trials are needed to evaluate its efficacy in HNC patients. However, according to the currently available data, copanlisib and PX-866 demonstrated unfavorable toxicity or no improvement in clinical outcomes when combined with cetuximab in R/M-HNSCC patients, regardless of HPV infection status.^487,488^

#### AKT inhibitors

Only three AKT inhibitors have been investigated against HNC, including MK-2206, perifosine, and ipatasertib. Serious adverse effects of AKT inhibitors include skin rash, hyperglycemia, fatigue, and ulcerative keratitis.^489–491^ Clinical data are still insufficient about the efficacy of AKT inhibitors in HNC. In a phase 2 clinical trial, perifosine failed to show single-agent activity in R/M-HNSCC patients, hindering further exploration of perifosine as monotherapy in this disease.^492^ The results of two ongoing phase 2 clinical trials will help us better understand how AKT inhibitors act in HNC patients (NCT01306045 and NCT05172258).

#### mTOR inhibitors

Current research on mTOR inhibitors in HNC has concentrated on two analogs of rapamycin, everolimus, and temsirolimus. In HNSCC patients, whether everolimus treatment brings more benefits than drawbacks remains controversial. Some phase 2 clinical trials indicated that everolimus had limited activity with a low response rate as a monotherapy or in combination with erlotinib.^493–495^ Two studies investigating the efficacy and safety of everolimus in combination with cisplatin and radiotherapy were even terminated as a result of funding problems and toxicity (NCT01057277, NCT01009346). However, other phase 2 studies reported that everolimus treatment displayed significant antitumor effects in aggressive radioiodine-refractory or advanced follicular-derived thyroid cancer, with a high rate of disease control, relatively low toxicity profile, and median PFS of 9 months.^496,497^ For temsirolimus, in the TEMHEAD phase 2 study, temsirolimus yielded modest antitumor activity in R/M-HNSCC patients with a PFS of 56 days and OS of 152 days,^498^ which is similar to the history of single-agent cetuximab in platinum-failed patients.^414^ Baseline caspase 3 activity is inversely correlated with PFS in temsirolimus-treated HNSCC patients, making it a potentially useful noninvasive biomarker of sensitivity to mTOR inhibitors.^499^ Recently, studies have focused on exploring effective combination strategies for temsirolimus. In a phase 2 study, temsirolimus plus low-dose chemotherapy (carboplatin and paclitaxel) induced a relatively high response rate (41.7%) and long survival (OS: 12.8 months; PFS: 5.9 months) compared to other treatments in R/M-HNSCC patients.^500^ However, there were no patients receiving only chemotherapy in this study, making it difficult to evaluate the additional benefits of temsirolimus. In another randomized phase 2 study, a combination of temsirolimus and cetuximab induced a significantly high response in cetuximab-resistant R/M-HNSCC patients compared with temsirolimus alone, although no significant improvement in PFS was observed, indicating that dual blockade of mTOR and EGFR pathways may be a potential strategy to overcome EGFR resistance.^501^

### C-MET inhibitors

C-MET overexpression is common in HNSCC patients, and overactivation of MET signaling contributes to resistance to anti-EGFR therapy.^108,502^ Most c-MET inhibitors are multitarget inhibitors that mainly inhibit the function of c-MET. For instance, cabozantinib is an oral inhibitor of multiple kinases, including c-MET, VEGFR-2, RET, KIT, AXL, and FLT3.^503^ In an international, randomized, placebo-controlled phase 3 clinical trial, cabozantinib monotherapy (140 mg/d) achieved a significant increase in objective response rate and PFS with acceptable toxicity in 330 patients with metastatic MTC.^504,505^ Prolonged PFS can be observed in all subgroups of patients with different ages, previous treatments and RET mutation statuses.^504,506^ Based on these results, in November 2012, cabozantinib received FDA approval for the treatment of metastatic MTC. Cabozantinib as salvage therapy for radioiodine-refractory DTC who progressed after treatment with VEGFR targeted therapy also increased the objective response rate by 15% and prolonged PFS, indicating the potential of cabozantinib as a treatment option for thyroid cancer patients with no available standard of care.^507,508^ Therefore, cabozantinib was soon approved as a second-line treatment for adult and pediatric patients with LA- or metastatic DTC who are ineligible or refractory to radioactive iodine. In contrast, other c-MET inhibitors, including tivantinib and foretinib, failed to show clinical benefits over risk in patients with R/M-HNSCC alone or combined with cetuximab.^509,510^

### RET inhibitors

Selpercatinib (LOXO-292) is an ATP-competitive, highly selective small molecule inhibitor of RET kinase. In the phase 1/2 LIBRETTO-001 trial, selpercatinib exhibited durable efficacy with mostly low-grade adverse effects in patients with RET-altered thyroid cancer, including RET-mutant medullary thyroid cancer and RET fusion-positive thyroid cancer.^511^ The response rate and PFS were drastically high in all groups of patients, regardless of previous vandetanib or cabozantinib treatment. Owing to the promising results, selpercatinib received accelerated approval for the treatment of patients with RET-mutant MTC or advanced or metastatic RET fusion-positive thyroid cancer in 2020. Since its initial approval, selpercatinib has altered the treatment paradigm for cancer patients with *RET* mutations. In 2022, the approval of selpercatinib has been expanded to all kinds of solid tumors with a RET gene fusion. Full approval of selpercatinib may be contingent on the confirmation of the clinical benefits from several ongoing phase 3 clinical trials (NCT04819100, NCT04211337, NCT04194944). Vandetanib is a once-daily oral triple-inhibitor of the RET, VEGFR, and EGFR signaling pathways. Vandetanib monotherapy successfully increased the objective response and survival of patients with advanced or metastatic thyroid cancer, with the estimated PFS reaching up to 30.5 months compared to placebo (19.3 months).^512^ Soon, the favorable efficacy of vandetanib promoted its FDA approval in treating advanced MTC in 2011. Pralsetinib is another selective and strong inhibitor of RET that also showed clinical activity and safety in RET-altered thyroid cancers in a phase 1/2 study.^513^ Further studies are needed to confirm its performance in the clinic.

### Other targeted therapies

**Fig. 6:**
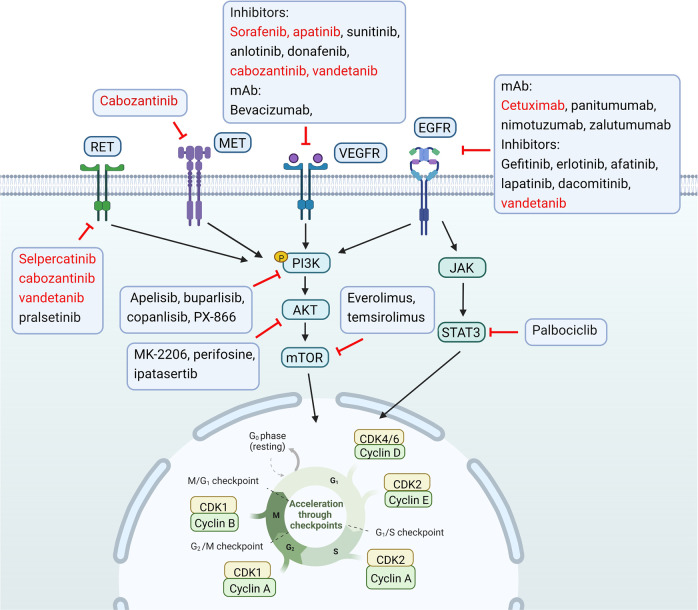
Comprehensive understanding and inhibitor direction for targeting signaling pathways in clinical trials in HNC treatment. EGFR epidermal growth factor receptor, MET mesenchymal–epithelial transition factor, AKT serine/threonine-specific protein kinase, mTOR mammalian target of rapamycin, CDK cyclin-dependent kinase, VEGF vascular endothelial growth factor, JAK jenus-activated kinase, STAT signal transducer and activator of transcription, mAb monoclonal antibody, RET rearranged during transfection, p phosphorylation

Some other signaling pathways can be targeted and evaluated in clinical trials for HNC, such as activin receptor-like kinase 1 (ALK1), CDK, STAT3, and indoleamine 2,3-dioxygenase 1 (IDO1). A comprehensive understanding and inhibitor directions for targeting signaling pathways in clinical trials in HNC treatment are shown in Fig. 6. Most of these therapeutic agents are still under clinical trials with limited data released about their efficacy and safety. Epacadostat is a strong and selective inhibitor of the IDO1 enzyme. It showed favorable safety and antitumor efficacy in combination with pembrolizumab in multiple advanced solid tumors, including HNSCC, in a multicenter, open-label phase 1/2 trial.^514^ A phase 3 study is ongoing to assess the efficacy and safety of epacadostat plus pembrolizumab, pembrolizumab monotherapy, and the EXTREME regimen in R/M-HNSCC patients (NCT03358472), the results of which will help us gain a comprehensive understanding of the clinical activity of epacadostat. Palbociclib, an inhibitor of CDK4/6, showed synergistic effects with cetuximab in patients with platinum and cetuximab double-resistant, HPV-unrelated HNSCC in a phase 2 study, suggesting its role in overcoming resistance to EGFR-targeted therapy.^423^ Dalantercept is a blocker of ALK1 signaling and angiogenesis, which showed a favorable safety profile but only modest activity in R/M-HNSCC patients as monotherapy.^515^

## Conclusion

Although aggressive treatment regimens of inhibitors, chemotherapy, radiotherapy, and surgical resection have been used in HNC, the treatment response in HNC is poor without significant improvement. The heterogeneity status of HNC led to the development of novel and specific effectors for identifying superior therapeutic regimens. Over the past decades, a comprehensive understanding of the pathogenesis, drug resistance mechanism, and alterations have been investigated in HNC. Various novel small molecular inhibitor monotherapies or combinations with other regimens have been developed in HNC in preclinical models and clinical trials. These new drugs have demonstrated remarkable responses and moderate adverse effects by inhibiting targeted gene expression. Compared with traditional regimens such as chemotherapy and radiotherapy, small molecular inhibitors result in fewer adverse effects and improve the tolerance of patients.

In this review, classic targets, such as the PI3K/AKT/mTOR pathway, EGFR signaling, VEGF signaling, FGFR signaling, and MEK/ERK signaling, are discussed. Some novel targets, including MET signaling, CDK4/6 signaling, and Notch signaling, were also involved in this research and need further investigation. EGFR signaling in HNC is the central signaling pathway in targeted treatment. EGFR is an essential effector in convergent signaling pathways, which is also the basis of oncogenic signaling inhibition. TP53 is one of the prominently mutated genes in HNC and is closely related to tumor progression, recurrence and therapeutic resistance. TP53 plays a critical role in regulating HNC. Other gene alterations facilitating the process of HNC are also discussed in this review.

Radiotherapy plays an important role in treating HNSCC. Furthermore, radioresistance leads to the treatment failure of advanced-stage HNSCC. Inhibitors promoting radiosensitivity are worth thoroughly research. Inhibitors of the PI3K/AKT/mTOR pathway, EGFR signaling, VEGF signaling, FGFR signaling, MEK/ERK signaling, MET signaling, CDK4/6 signaling, and Notch signaling have been investigated in promoting radiosensitivity.

Chemotherapy and chemoradiotherapy have been shown to improve clinical outcomes and are considered standard-of-care strategies for HNSCC. Cisplatin-based regimens were the most commonly used combination as a conventional treatment strategy or ICT. However, chemotherapy-induced toxicity is a major concern in HNSCC treatment. Therefore, efforts are focusing on developing multicombination therapy with improved efficacy and safety. Great success has been achieved with the development of cetuximab, which has been approved for the treatment of R/M-HNSCC patients in combination with platinum-based therapy and fluorouracil as first-line therapy. Inspired by the triad, scientists are exploring new combination strategies involving other targeted therapeutics and chemotherapeutics. Some results are promising, while others are obscure. For instance, a combination of panitumumab, cisplatin, and fluorouracil prolonged survival in R/M-HNSCC patients when compared with chemoradiotherapy.^427^ In contrast, it has been reported that adding cetuximab to the TPF ICT regimen showed no superiority in clinical outcomes over ICT alone in the DeLOS-II trial.^397^ Therefore, more studies are necessary to guide the application of targeted therapies and chemotherapies.

CDK4/6 inhibition provides new hope for HPV-negative tumor patients. The response to CDK4/6 inhibitors in HPV-negative HNSCC patients was favorable.^516^ HPV-positive HNSCC patients could not benefit from CDK4/6 inhibitors. However, it seems that not all HPV-negative HNSCC patients could benefit from inhibitors. Further investigation of CDK4/6 inhibitors in HPV-negative HNSCC patients should be developed.^517,518^ The mechanism of CDK4/6 resistance was also explored, which provided considerate predictive biomarkers in predicting the prognosis of the CDK4/6 response.

RET inhibitors have shown remarkable effects in clinical trials. Owing to the promising results, selpercatinib received accelerated approval for the treatment of patients with RET-mutant MTC or advanced or metastatic RET fusion-positive thyroid cancer in 2020. In 2022, the approval of selpercatinib has been expanded to all kinds of solid tumors with a RET gene fusion. Further administration of RET inhibitors is expected.

Although targeted therapies have achieved satisfactory effects in selected patients with specific alterations, the effect of some targeted therapies is far from desirable. Many inhibitors have been approved for treating patients with EGFR alterations. Gefitinib is reported to be effective in NSCLC patients with EGFR exon 19 deletions or exon 21 (L858R) mutations, which might result in undesirable effects in ~15% of HNSCC patients.^519^ In patients with an unfavorable response rate to EGFR-targeted therapies, some articles demonstrated that these patients may also develop drug resistance.^520,521^ It was reported that multiple downstream effectors served as substitutive signaling and were discovered to be continuously activated, which led to EGFR inhibitor resistance.^522^ Only 5% of HNC patients have EGFR alterations, which may contribute to the limited effectiveness of tyrosine kinase inhibitors.^523^ Genetic sequencing was used to identify specific targets among various types of cancers. However, enormous genetic heterogeneity and precise alterations in genomes result in targeted therapy failure in specific selected humans. Therefore, it is important to identify the heterogeneity of tumors to help choose efficient small molecule inhibitors.

The response to targeted therapies relies on its specific target in tumor tissue. However, off-target side effects may lead to treatment failure and severe adverse effects.^524^ The bystander effect may also result in treatment failure. Antibody-directed enzyme prodrug therapy consists of a tumor-specific antibody and a drug-activating enzyme, which may promote the antibody action on targeted tissues.^525–527^ Some small molecule inhibitors belonging to antibody-directed enzyme prodrug therapy exist between antigen-positive and antigen-negative cancer cells, leading to the bystander effect.^525^ The bystander effect could be avoided by regulating the interval time between the enzyme and drugs.

This information offered new insights into treating HNC and different cancers at distinct sites with various pathological subtypes that can be treated by various regimens. The rate of survival and severe toxicities was improved after novel treatment regimens. Multiple studies in progress could contribute to the specific use of novel compounds in therapeutic strategies and precisely identify patients with favorable outcomes via relatively predictive effectors. Due to moderate adverse effects, small molecular inhibitors are still in urgent demand, but the treatment response needs further improvement. Collectively, thorough and comprehensive studies offer more knowledge about the mechanisms of HNC, which has a potential role in preventing and treating HNC. The clinical translation of new inhibitors is still crucial, and combining new agents with traditional regimens also has great potency in treating HNC.

## References

[1] Sung H (2021). Global cancer statistics 2020: GLOBOCAN estimates of incidence and mortality worldwide for 36 cancers in 185 countries. CA Cancer J. Clin..

[2] Leemans CR, Snijders PJF, Brakenhoff RH (2018). The molecular landscape of head and neck cancer. Nat. Rev. Cancer.

[3] Ferlay J (2019). Estimating the global cancer incidence and mortality in 2018: GLOBOCAN sources and methods. Int. J. Cancer.

[4] Yan, F. et al. The evolution of care of cancers of the head and neck region: state of the science in 2020. *Cancers* **12**, 1543 (2020). 10.3390/cancers12061543 PMC7352543 32545409

[5] Johnson DE (2020). Head and neck squamous cell carcinoma. Nat. Rev. Dis. Prim..

[6] Chien YC (2001). Serologic markers of Epstein-Barr virus infection and nasopharyngeal carcinoma in Taiwanese men. N. Engl. J. Med..

[7] Ferris RL (2016). Nivolumab for recurrent squamous-cell carcinoma of the head and neck. N. Engl. J. Med..

[8] Seiwert TY (2016). Safety and clinical activity of pembrolizumab for treatment of recurrent or metastatic squamous cell carcinoma of the head and neck (KEYNOTE-012): an open-label, multicentre, phase 1b trial. Lancet Oncol..

[9] Burtness B (2019). Pembrolizumab alone or with chemotherapy versus cetuximab with chemotherapy for recurrent or metastatic squamous cell carcinoma of the head and neck (KEYNOTE-048): a randomised, open-label, phase 3 study. Lancet.

[10] Van den Bossche V (2022). Microenvironment-driven intratumoral heterogeneity in head and neck cancers: clinical challenges and opportunities for precision medicine. Drug Resist. Updat..

[11] Kang Y (2020). Advances in targeted therapy mainly based on signal pathways for nasopharyngeal carcinoma. Signal Transduct. Target Ther..

[12] Caetano RS, Lima FF, Gomes EP, Volpato LE (2022). Quality of life of patients after treatment for cancer in the head and neck region: a case-control study. Cureus.

[13] Brook I (2020). Late side effects of radiation treatment for head and neck cancer. Radiat. Oncol. J..

[14] Lazarus CL (2009). Effects of chemoradiotherapy on voice and swallowing. Curr. Opin. Otolaryngol. Head. Neck Surg..

[15] Hendler FJ, Ozanne BW (1984). Human squamous cell lung cancers express increased epidermal growth factor receptors. J. Clin. Invest..

[16] Wells A (1999). EGF receptor. Int. J. Biochem Cell Biol..

[17] Sibilia M (1998). A strain-independent postnatal neurodegeneration in mice lacking the EGF receptor. EMBO J..

[18] de Jong JS, van Diest PJ, van der Valk P, Baak JP (1998). Expression of growth factors, growth-inhibiting factors, and their receptors in invasive breast cancer. II: correlations with proliferation and angiogenesis. J. Pathol..

[19] Wells A (2000). Tumor invasion: role of growth factor-induced cell motility. Adv. Cancer Res..

[20] Kabolizadeh P (2012). The role of cetuximab in the management of head and neck cancers. Expert Opin. Biol. Ther..

[21] Specenier P, Vermorken JB (2013). Cetuximab: its unique place in head and neck cancer treatment. Biologics.

[22] Krishnamurthy S (2022). The dogma of cetuximab and radiotherapy in head and neck cancer—a dawn to dusk journey. Clin. Transl. Radiat. Oncol..

[23] Baselga J (2000). Phase I studies of anti-epidermal growth factor receptor chimeric antibody C225 alone and in combination with cisplatin. J. Clin. Oncol..

[24] Robert F (2001). Phase I study of anti-epidermal growth factor receptor antibody cetuximab in combination with radiation therapy in patients with advanced head and neck cancer. J. Clin. Oncol..

[25] Bonner JA (2006). Radiotherapy plus cetuximab for squamous-cell carcinoma of the head and neck. N. Engl. J. Med..

[26] Vermorken JB (2008). Platinum-based chemotherapy plus cetuximab in head and neck cancer. N. Engl. J. Med..

[27] Singh P (2021). Real-world treatment patterns and outcomes in patients with head and neck cancer: point-in-time survey of oncologists in Italy and Spain. Adv. Ther..

[28] Solomon B, Young RJ, Rischin D (2018). Head and neck squamous cell carcinoma: genomics and emerging biomarkers for immunomodulatory cancer treatments. Semin. cancer Biol..

[29] Xu MJ, Johnson DE, Grandis JR (2017). EGFR-targeted therapies in the post-genomic era. Cancer Metastasis Rev..

[30] Byeon HK, Ku M, Yang J (2019). Beyond EGFR inhibition: multilateral combat strategies to stop the progression of head and neck cancer. Exp. Mol. Med..

[31] Harari PM, Wheeler DL, Grandis JR (2009). Molecular target approaches in head and neck cancer: epidermal growth factor receptor and beyond. Semin. Radiat. Oncol..

[32] Kalyankrishna S, Grandis JR (2006). Epidermal growth factor receptor biology in head and neck cancer. J. Clin. Oncol..

[33] Zhang J, Saba NF, Chen GZ, Shin DM (2015). Targeting HER (ERBB) signaling in head and neck cancer: an essential update. Mol. Asp. Med..

[34] Brand TM (2013). Nuclear EGFR as a molecular target in cancer. Radiother. Oncol.: J. Eur. Soc. Therapeutic Radiol. Oncol..

[35] Vouri M (2016). Axl-EGFR receptor tyrosine kinase hetero-interaction provides EGFR with access to pro-invasive signalling in cancer cells. Oncogenesis.

[36] Lo HW (2010). Nuclear mode of the EGFR signaling network: biology, prognostic value, and therapeutic implications. Discov. Med..

[37] Marquard FE, Jucker M (2020). PI3K/AKT/mTOR signaling as a molecular target in head and neck cancer. Biochem. Pharm..

[38] Wang Z (2017). mTOR co-targeting strategies for head and neck cancer therapy. Cancer Metastasis Rev..

[39] Kang H, Kiess A, Chung CH (2015). Emerging biomarkers in head and neck cancer in the era of genomics. Nat. Rev. Clin. Oncol..

[40] Gutkind JS, Day TA, Lippman SM, Szabo E (2019). Targeting mTOR in head and neck cancer-response. Clin. Cancer Res..

[41] Farah CS (2021). Molecular landscape of head and neck cancer and implications for therapy. Ann. Transl. Med..

[42] Seiwert TY (2015). Integrative and comparative genomic analysis of HPV-positive and HPV-negative head and neck squamous cell carcinomas. Clin. Cancer Res..

[43] Sun Y, Wang Z, Qiu S, Wang R (2021). Therapeutic strategies of different HPV status in head and neck squamous cell carcinoma. Int. J. Biol. Sci..

[44] Lee MJ, Jin N, Grandis JR, Johnson DE (2020). Alterations and molecular targeting of the GSK-3 regulator, PI3K, in head and neck cancer. Biochimica et. Biophysica Acta Mol. Cell Res..

[45] Liu P, Cheng H, Roberts TM, Zhao JJ (2009). Targeting the phosphoinositide 3-kinase pathway in cancer. Nat. Rev. Drug Discov..

[46] Engelman JA, Luo J, Cantley LC (2006). The evolution of phosphatidylinositol 3-kinases as regulators of growth and metabolism. Nat. Rev. Genet..

[47] Zhang P, Steinberg BM (2000). Overexpression of PTEN/MMAC1 and decreased activation of Akt in human papillomavirus-infected laryngeal papillomas. Cancer Res..

[48] Maehama T, Dixon JE (1998). The tumor suppressor, PTEN/MMAC1, dephosphorylates the lipid second messenger, phosphatidylinositol 3,4,5-trisphosphate. J. Biol. Chem..

[49] Kim YC, Guan KL (2015). mTOR: a pharmacologic target for autophagy regulation. J. Clin. Invest..

[50] LoPiccolo J, Blumenthal GM, Bernstein WB, Dennis PA (2008). Targeting the PI3K/Akt/mTOR pathway: effective combinations and clinical considerations. Drug Resist. Updat..

[51] Vander Broek R (2015). The PI3K/Akt/mTOR axis in head and neck cancer: functions, aberrations, cross-talk, and therapies. Oral. Dis..

[52] Tan, F. H., Bai, Y., Saintigny, P. & Darido, C. mTOR signalling in head and neck cancer: heads up. *Cells* **8**, 333 (2019). 10.3390/cells8040333 PMC6523933 30970654

[53] Or YY (2006). PIK3CA mutations in nasopharyngeal carcinoma. Int. J. Cancer.

[54] García-Escudero R (2018). Overexpression of PIK3CA in head and neck squamous cell carcinoma is associated with poor outcome and activation of the YAP pathway. Oral. Oncol..

[55] Cohen Y (2011). Mutational analysis of PTEN/PIK3CA/AKT pathway in oral squamous cell carcinoma. Oral. Oncol..

[56] Squarize CH (2013). PTEN deficiency contributes to the development and progression of head and neck cancer. Neoplasia.

[57] Izumi H (2020). Pathway-specific genome editing of PI3K/mTOR tumor suppressor genes reveals that PTEN loss contributes to cetuximab resistance in head and neck cancer. Mol. cancer therapeutics.

[58] Glorieux M, Dok R, Nuyts S (2020). The influence of PI3K inhibition on the radiotherapy response of head and neck cancer cells. Sci. Rep..

[59] Xia A (2018). Co-treatment with BEZ235 enhances chemosensitivity of A549/DDP cells to cisplatin via inhibition of PI3K/Akt/mTOR signaling and downregulation of ERCC1 expression. Oncol. Rep..

[60] Bruce JP (2015). Nasopharyngeal cancer: molecular landscape. J. Clin. Oncol..

[61] Horikawa T (2011). Epstein-Barr virus latent membrane protein 1 induces Snail and epithelial-mesenchymal transition in metastatic nasopharyngeal carcinoma. Br. J. Cancer.

[62] Li SS (2011). Latent membrane protein 1 mediates the resistance of nasopharyngeal carcinoma cells to TRAIL-induced apoptosis by activation of the PI3K/Akt signaling pathway. Oncol. Rep..

[63] Yang CF (2016). EB-virus latent membrane protein 1 potentiates the stemness of nasopharyngeal carcinoma via preferential activation of PI3K/AKT pathway by a positive feedback loop. Oncogene.

[64] Lo AK (2018). Activation of sterol regulatory element-binding protein 1 (SREBP1)-mediated lipogenesis by the Epstein-Barr virus-encoded latent membrane protein 1 (LMP1) promotes cell proliferation and progression of nasopharyngeal carcinoma. J. Pathol..

[65] Luo X (2018). DNMT1 mediates metabolic reprogramming induced by Epstein-Barr virus latent membrane protein 1 and reversed by grifolin in nasopharyngeal carcinoma. Cell Death Dis..

[66] Xiang T (2018). Vasculogenic mimicry formation in EBV-associated epithelial malignancies. Nat. Commun..

[67] Braicu, C. et al. A comprehensive review on MAPK: a promising therapeutic target in cancer. *Cancers* **11**, 1618 (2019). 10.3390/cancers11101618 PMC6827047 31652660

[68] Ullah, R., Yin, Q., Snell, A. H. & Wan, L. RAF-MEK-ERK pathway in cancer evolution and treatment. *Semin. Cancer Biol.* **85**, 123–154 (2021). 10.1016/j.semcancer.2021.05.010 33992782

[69] Degirmenci, U., Wang, M. & Hu, J. Targeting aberrant RAS/RAF/MEK/ERK signaling for cancer therapy. *Cells* **9**, 198 (2020). 10.3390/cells9010198 PMC7017232 31941155

[70] Ngan HL (2022). Precision drugging of the MAPK pathway in head and neck cancer. NPJ Genom. Med..

[71] Barbosa R, Acevedo LA, Marmorstein R (2021). The MEK/ERK network as a therapeutic target in human cancer. Mol. Cancer Res.: MCR.

[72] Samatar AA, Poulikakos PI (2014). Targeting RAS-ERK signalling in cancer: promises and challenges. Nat. Rev. Drug Discov..

[73] Ngan HL (2020). MAPK pathway mutations in head and neck cancer affect immune microenvironments and ErbB3 signaling. Life Sci. Alliance.

[74] Asati V, Mahapatra DK, Bharti SK (2016). PI3K/Akt/mTOR and Ras/Raf/MEK/ERK signaling pathways inhibitors as anticancer agents: structural and pharmacological perspectives. Eur. J. Medicinal Chem..

[75] De Luca A (2012). The RAS/RAF/MEK/ERK and the PI3K/AKT signalling pathways: role in cancer pathogenesis and implications for therapeutic approaches. Expert Opin. Ther. Targets.

[76] Gkouveris I (2014). Erk1/2 activation and modulation of STAT3 signaling in oral cancer. Oncol. Rep..

[77] Zhang L (2017). Genomic analysis of nasopharyngeal carcinoma reveals TME-based subtypes. Mol. Cancer Res.: MCR.

[78] Li YY (2017). Exome and genome sequencing of nasopharynx cancer identifies NF-κB pathway activating mutations. Nat. Commun..

[79] Peng Q (2018). Mitogen-activated protein kinase signaling pathway in oral cancer. Oncol. Lett..

[80] Wee, P. & Wang, Z. Epidermal growth factor receptor cell proliferation signaling pathways. *Cancers* **9**, 52 (2017). 10.3390/cancers9050052 PMC5447962 28513565

[81] Li Z, Li N, Shen L (2018). MAP2K6 is associated with radiation resistance and adverse prognosis for locally advanced nasopharyngeal carcinoma patients. Cancer Manag. Res..

[82] King AJ (1998). The protein kinase Pak3 positively regulates Raf-1 activity through phosphorylation of serine 338. Nature.

[83] Nikitakis NG, Siavash H, Sauk JJ (2004). Targeting the STAT pathway in head and neck cancer: recent advances and future prospects. Curr. Cancer Drug Targets.

[84] Leeman RJ, Lui VW, Grandis JR (2006). STAT3 as a therapeutic target in head and neck cancer. Expert Opin. Biol. Ther..

[85] Geiger JL, Grandis JR, Bauman JE (2016). The STAT3 pathway as a therapeutic target in head and neck cancer: Barriers and innovations. Oral. Oncol..

[86] Sen M (2015). JAK kinase inhibition abrogates STAT3 activation and head and neck squamous cell carcinoma tumor growth. Neoplasia.

[87] Bu LL (2017). STAT3 induces immunosuppression by upregulating PD-1/PD-L1 in HNSCC. J. Dent. Res..

[88] De Carvalho TG (2013). Search for mutations in signaling pathways in head and neck squamous cell carcinoma. Oncol. Rep..

[89] Yu H (2014). Revisiting STAT3 signalling in cancer: new and unexpected biological functions. Nat. Rev. Cancer.

[90] Lai SY, Johnson FM (2010). Defining the role of the JAK-STAT pathway in head and neck and thoracic malignancies: implications for future therapeutic approaches. Drug Resist. Updat..

[91] Lai SY (2005). Erythropoietin-mediated activation of JAK-STAT signaling contributes to cellular invasion in head and neck squamous cell carcinoma. Oncogene.

[92] Song JI, Grandis JR (2000). STAT signaling in head and neck cancer. Oncogene.

[93] Mali SB (2015). Review of STAT3 (signal transducers and activators of transcription) in head and neck cancer. Oral. Oncol..

[94] Morgan, E. L. & Macdonald, A. Manipulation of JAK/STAT signalling by high-risk HPVs: potential therapeutic targets for HPV-associated malignancies. *Viruses* **12**, 977 (2020). 10.3390/v12090977 PMC7552066 32899142

[95] Zou S (2020). Targeting STAT3 in cancer immunotherapy. Mol. Cancer.

[96] Hartmann S, Bhola NE, Grandis JR (2016). HGF/Met signaling in head and neck cancer: impact on the tumor microenvironment. Clin. Cancer Res..

[97] Cho YA (2016). Alteration status and prognostic value of MET in head and neck squamous cell carcinoma. J. Cancer.

[98] Arnold, L., Enders, J. & Thomas, S. M. Activated HGF-c-Met axis in head and neck cancer. *Cancers* **9**, 169 (2017). 10.3390/cancers9120169 PMC5742817 29231907

[99] Rothenberger, N. J. & Stabile, L. P. Hepatocyte growth factor/c-met signaling in head and neck cancer and implications for treatment. *Cancers* **9**, 39 (2017). 10.3390/cancers9040039 PMC5406714 28441771

[100] Fu J (2021). HGF/c-MET pathway in cancer: from molecular characterization to clinical evidence. Oncogene.

[101] Cecchi F, Rabe DC, Bottaro DP (2010). Targeting the HGF/Met signalling pathway in cancer. Eur. J. Cancer.

[102] De Silva DM (2017). Targeting the hepatocyte growth factor/Met pathway in cancer. Biochemical Soc. Trans..

[103] Lim YC, Kang HJ, Moon JH (2014). C-Met pathway promotes self-renewal and tumorigenecity of head and neck squamous cell carcinoma stem-like cell. Oral. Oncol..

[104] Raj S (2022). Molecular mechanism(s) of regulation(s) of c-MET/HGF signaling in head and neck cancer. Mol. Cancer.

[105] Liu D (2020). Roles of the HGF/Met signaling in head and neck squamous cell carcinoma: focus on tumor immunity (review). Oncol. Rep..

[106] Tsang CM (2020). Translational genomics of nasopharyngeal cancer. Semin. Cancer Biol..

[107] Bruce JP (2021). Whole-genome profiling of nasopharyngeal carcinoma reveals viral-host co-operation in inflammatory NF-κB activation and immune escape. Nat. Commun..

[108] Cancer Genome Atlas Network. Comprehensive genomic characterization of head and neck squamous cell carcinomas. *Nature* **517**, 576–582 (2015). 10.1038/nature14129 PMC4311405 25631445

[109] Marur S, D’Souza G, Westra WH, Forastiere AA (2010). HPV-associated head and neck cancer: a virus-related cancer epidemic. Lancet Oncol..

[110] Castellsagué X (2016). HPV involvement in head and neck cancers: comprehensive assessment of biomarkers in 3680 patients. J. Natl Cancer Inst..

[111] Sullivan KD, Galbraith MD, Andrysik Z, Espinosa JM (2018). Mechanisms of transcriptional regulation by p53. Cell Death Differ..

[112] Kaiser AM, Attardi LD (2018). Deconstructing networks of p53-mediated tumor suppression in vivo. Cell Death Differ..

[113] Bykov VJN, Eriksson SE, Bianchi J, Wiman KG (2018). Targeting mutant p53 for efficient cancer therapy. Nat. Rev. Cancer.

[114] Aubrey BJ (2018). How does p53 induce apoptosis and how does this relate to p53-mediated tumour suppression?. Cell Death Differ..

[115] Levine AJ (2018). Reviewing the future of the P53 field. Cell Death Differ..

[116] Huang L (2011). The p53 inhibitors MDM2/MDMX complex is required for control of p53 activity in vivo. Proc. Natl Acad. Sci. USA.

[117] Duffy MJ, Synnott NC, O’Grady S, Crown J (2022). Targeting p53 for the treatment of cancer. Semin. Cancer Biol..

[118] Si H (2016). TNF-α modulates genome-wide redistribution of ΔNp63α/TAp73 and NF-κB cREL interactive binding on TP53 and AP-1 motifs to promote an oncogenic gene program in squamous cancer. Oncogene.

[119] Rothenberg SM, Ellisen LW (2012). The molecular pathogenesis of head and neck squamous cell carcinoma. J. Clin. Invest..

[120] Rocco JW (2006). p63 mediates survival in squamous cell carcinoma by suppression of p73-dependent apoptosis. Cancer Cell.

[121] Lo Muzio L (2005). p63 overexpression associates with poor prognosis in head and neck squamous cell carcinoma. Hum. Pathol..

[122] Cai, B. H. et al. P63 and P73 activation in cancers with p53 mutation. *Biomedicines* **10**, (2022). 10.3390/biomedicines10071490 PMC9313412 35884795

[123] Moses, M. A. et al. Molecular mechanisms of p63-mediated squamous cancer pathogenesis. *Int. J. Mol. Sci*. **20**, 3590 (2019). 10.3390/ijms20143590 PMC6678256 31340447

[124] Compagnone M (2017). ΔNp63-mediated regulation of hyaluronic acid metabolism and signaling supports HNSCC tumorigenesis. Proc. Natl Acad. Sci. USA.

[125] Hu Y (2012). Association between the p73 exon 2 G4C14-to-A4T14 polymorphism and cancer risk: a meta-analysis. DNA Cell Biol..

[126] Nemajerova, A. & Moll, U. M. Tissue-specific roles of p73 in development and homeostasis. *J. Cell Sci.* **132**, jcs233338 (2019). 10.1242/jcs.233338 PMC6803362 31582429

[127] Rozenberg, J. M. et al. Dual role of p73 in cancer microenvironment and DNA damage response. *Cells* **10**, 3516 (2021). 10.3390/cells10123516 PMC8700694 34944027

[128] Maas AM, Bretz AC, Mack E, Stiewe T (2013). Targeting p73 in cancer. Cancer Lett..

[129] Rozenberg JM (2021). The p53 family member p73 in the regulation of cell stress response. Biol. Direct.

[130] Klanrit P (2009). PML involvement in the p73-mediated E1A-induced suppression of EGFR and induction of apoptosis in head and neck cancers. Oncogene.

[131] Ramos H, Raimundo L, Saraiva L (2020). p73: From the p53 shadow to a major pharmacological target in anticancer therapy. Pharmacol. Res..

[132] Yoon MK, Ha JH, Lee MS, Chi SW (2015). Structure and apoptotic function of p73. BMB Rep..

[133] Engeland K (2022). Cell cycle regulation: p53-p21-RB signaling. Cell Death Differ..

[134] Chen L, Liu S, Tao Y (2020). Regulating tumor suppressor genes: post-translational modifications. Signal Transduct. Target Ther..

[135] Gipson BJ, Robbins HA, Fakhry C, D’Souza G (2018). Sensitivity and specificity of oral HPV detection for HPV-positive head and neck cancer. Oral. Oncol..

[136] Mitchell S, Vargas J, Hoffmann A (2016). Signaling via the NFκB system. Wiley Interdiscip. Rev. Syst. Biol. Med..

[137] DiDonato JA, Mercurio F, Karin M (2012). NF-κB and the link between inflammation and cancer. Immunological Rev..

[138] Vander Broek R, Snow GE, Chen Z, Van Waes C (2014). Chemoprevention of head and neck squamous cell carcinoma through inhibition of NF-κB signaling. Oral. Oncol..

[139] Zhang Q, Lenardo MJ, Baltimore D (2017). 30 Years of NF-κB: a blossoming of relevance to human pathobiology. Cell.

[140] Smale ST (2012). Dimer-specific regulatory mechanisms within the NF-κB family of transcription factors. Immunological Rev..

[141] Monisha J (2017). Nuclear factor kappa b: a potential target to persecute head and neck cancer. Curr. Drug Targets.

[142] Prasad S, Ravindran J, Aggarwal BB (2010). NF-kappaB and cancer: how intimate is this relationship. Mol. Cell. Biochem..

[143] Yi M (2018). Rediscovery of NF-κB signaling in nasopharyngeal carcinoma: How genetic defects of NF-κB pathway interplay with EBV in driving oncogenesis?. J. Cell. Physiol..

[144] Campion NJ (2021). The molecular march of primary and recurrent nasopharyngeal carcinoma. Oncogene.

[145] Oeckinghaus A, Hayden MS, Ghosh S (2011). Crosstalk in NF-κB signaling pathways. Nat. Immunol..

[146] Gilmore TD (2006). Introduction to NF-kappaB: players, pathways, perspectives. Oncogene.

[147] Yu H (2020). Targeting NF-κB pathway for the therapy of diseases: mechanism and clinical study. Signal Transduct. Target Ther..

[148] Van Waes C (2007). Nuclear factor-kappaB in development, prevention, and therapy of cancer. Clin. Cancer Res..

[149] Allen CT, Ricker JL, Chen Z, Van Waes C (2007). Role of activated nuclear factor-kappaB in the pathogenesis and therapy of squamous cell carcinoma of the head and neck. Head. neck.

[150] Li L, Zhang ZT (2019). Genetic association between NFKBIA and NFKB1 gene polymorphisms and the susceptibility to head and neck cancer: a meta-analysis. Dis. Markers.

[151] Fan Y, Mao R, Yang J (2013). NF-κB and STAT3 signaling pathways collaboratively link inflammation to cancer. Protein Cell.

[152] King KE (2019). Intersection of the p63 and NF-κB pathways in epithelial homeostasis and disease. Mol. Carcinogenesis.

[153] Lee TL (2007). A novel nuclear factor-kappaB gene signature is differentially expressed in head and neck squamous cell carcinomas in association with TP53 status. Clin. Cancer Res..

[154] Clevers H, Nusse R (2012). Wnt/β-catenin signaling and disease. Cell.

[155] Liu F, Millar SE (2010). Wnt/beta-catenin signaling in oral tissue development and disease. J. Dent. Res..

[156] Cui C (2018). Is β-catenin a druggable target for cancer therapy?. Trends biochemical Sci..

[157] Aminuddin A, Ng PY (2016). Promising druggable target in head and neck squamous cell carcinoma: Wnt signaling. Front. Pharmacol..

[158] Xie J, Huang L, Lu YG, Zheng DL (2020). Roles of the Wnt signaling pathway in head and neck squamous cell carcinoma. Front. Mol. Biosci..

[159] Yu F (2021). Wnt/β-catenin signaling in cancers and targeted therapies. Signal Transduct. Target Ther..

[160] Nusse R, Clevers H (2017). Wnt/β-catenin signaling, disease, and emerging therapeutic modalities. Cell.

[161] Paluszczak, J. The significance of the dysregulation of canonical Wnt signaling in head and neck squamous cell carcinomas. *Cells* **9**, 723 (2020). 10.3390/cells9030723 PMC7140616 32183420

[162] Reyes, M. et al. Wnt/β-catenin signaling in oral carcinogenesis. *Int. J. Mol. Sci.* **21**, 4682 (2020). 10.3390/ijms21134682 PMC7369957 32630122

[163] Torres VI, Godoy JA, Inestrosa NC (2019). Modulating Wnt signaling at the root: porcupine and Wnt acylation. Pharmacol. Ther..

[164] Alamoud KA, Kukuruzinska MA (2018). Emerging insights into Wnt/β-catenin signaling in head and neck cancer. J. Dent. Res..

[165] Zhang X, Dong S, Xu F (2018). Structural and druggability landscape of frizzled G protein-coupled receptors. Trends Biochem. Sci..

[166] MacDonald, B. T. & He, X. Frizzled and LRP5/6 receptors for Wnt/β-catenin signaling. *Cold Spring Harbor Persp. Biol.* **4**, a007880 (2012). 10.1101/cshperspect.a007880 PMC3504444 23209147

[167] Ren Q, Chen J, Liu Y (2021). LRP5 and LRP6 in Wnt signaling: similarity and divergence. Front. Cell Dev. Biol..

[168] Valenta T, Hausmann G, Basler K (2012). The many faces and functions of β-catenin. EMBO J..

[169] Reya T, Clevers H (2005). Wnt signalling in stem cells and cancer. Nature.

[170] Graham TA (2000). Crystal structure of a beta-catenin/Tcf complex. Cell.

[171] Guan Z (2014). SOX1 down-regulates β-catenin and reverses malignant phenotype in nasopharyngeal carcinoma. Mol. cancer.

[172] Chan SL (2007). The tumor suppressor Wnt inhibitory factor 1 is frequently methylated in nasopharyngeal and esophageal carcinomas. Lab. Investig..

[173] Collu GM, Hidalgo-Sastre A, Brennan K (2014). Wnt-Notch signalling crosstalk in development and disease. Cell. Mol. Life Sci.: CMLS.

[174] Patni AP (2021). Comprehending the crosstalk between Notch, Wnt and Hedgehog signaling pathways in oral squamous cell carcinoma - clinical implications. Cell. Oncol..

[175] Porcheri, C., Meisel, C. T. & Mitsiadis, T. Multifactorial contribution of notch signaling in head and neck squamous cell carcinoma. *Int. J. Mol. Sci.* **20**, 1520 (2019). 10.3390/ijms20061520 PMC6471940 30917608

[176] Fukusumi T, Califano JA (2018). The NOTCH pathway in head and neck squamous cell carcinoma. J. Dent. Res..

[177] Borggrefe T (2016). The Notch intracellular domain integrates signals from Wnt, Hedgehog, TGFβ/BMP and hypoxia pathways. Biochimica et. Biophysica Acta.

[178] Lee SH (2016). Notch1 signaling contributes to stemness in head and neck squamous cell carcinoma. Lab. Investig..

[179] Shah, P. A. et al. NOTCH1 signaling in head and neck squamous cell carcinoma. *Cells* **9**, 2677 (2020). 10.3390/cells9122677 PMC7764697 33322834

[180] Dotto GP (2009). Crosstalk of Notch with p53 and p63 in cancer growth control. Nat. Rev. Cancer.

[181] Nguyen BC (2006). Cross-regulation between Notch and p63 in keratinocyte commitment to differentiation. Genes Dev..

[182] Porcheri C, Mitsiadis TA (2021). Notch in head and neck cancer. Adv. Exp. Med. Biol..

[183] Nowell CS, Radtke F (2017). Notch as a tumour suppressor. Nat. Rev. Cancer.

[184] Vassilakopoulou M, Psyrri A, Argiris A (2015). Targeting angiogenesis in head and neck cancer. Oral. Oncol..

[185] Lim SC (2005). Expression of c-erbB receptors, MMPs and VEGF in head and neck squamous cell carcinoma. Biomedicine Pharmacother. = Biomedecine pharmacotherapie.

[186] Zang J (2013). Prognostic value of vascular endothelial growth factor in patients with head and neck cancer: a meta-analysis. Head. neck.

[187] Yue B (2014). Knockdown of neuropilin-1 suppresses invasion, angiogenesis, and increases the chemosensitivity to doxorubicin in osteosarcoma cells - an in vitro study. Eur. Rev. Med. Pharmacol. Sci..

[188] Christopoulos A, Ahn SM, Klein JD, Kim S (2011). Biology of vascular endothelial growth factor and its receptors in head and neck cancer: beyond angiogenesis. Head Neck.

[189] Hsu HW (2014). Combination antiangiogenic therapy and radiation in head and neck cancers. Oral. Oncol..

[190] Seiwert TY, Cohen EE (2008). Targeting angiogenesis in head and neck cancer. Semin. Oncol..

[191] Salvatore D, Santoro M, Schlumberger M (2021). The importance of the RET gene in thyroid cancer and therapeutic implications. Nat. Rev. Endocrinol..

[192] Belli C (2020). Progresses toward precision medicine in RET-altered solid tumors. Clin. Cancer Res..

[193] Kawai K, Takahashi M (2020). Intracellular RET signaling pathways activated by GDNF. Cell Tissue Res..

[194] Drilon A, Hu ZI, Lai GGY, Tan DSW (2018). Targeting RET-driven cancers: lessons from evolving preclinical and clinical landscapes. Nat. Rev. Clin. Oncol..

[195] Subbiah V, Cote GJ (2020). Advances in targeting RET-dependent cancers. Cancer Discov..

[196] Perri F (2015). Targeted therapy: a new hope for thyroid carcinomas. Crit. Rev. Oncol./Hematol..

[197] Ernani V, Kumar M, Chen AY, Owonikoko TK (2016). Systemic treatment and management approaches for medullary thyroid cancer. Cancer Treat. Rev..

[198] Li AY (2019). RET fusions in solid tumors. Cancer Treat. Rev..

[199] Subbiah V (2020). State-of-the-art strategies for targeting RET-dependent cancers. J. Clin. Oncol..

[200] Cloer EW (2019). NRF2 activation in cancer: from DNA to protein. Cancer Res..

[201] Robertson, H., Dinkova-Kostova, A. T. & Hayes, J. D. NRF2 and the ambiguous consequences of its activation during initiation and the subsequent stages of tumourigenesis. *Cancers* **12**, 3609 (2020). 10.3390/cancers12123609 PMC7761610 33276631

[202] Namani A, Matiur Rahaman M, Chen M, Tang X (2018). Gene-expression signature regulated by the KEAP1-NRF2-CUL3 axis is associated with a poor prognosis in head and neck squamous cell cancer. BMC Cancer.

[203] Baird, L. & Yamamoto, M. The molecular mechanisms regulating the KEAP1-NRF2 pathway. *Mol. Cell. Biology*. **40**, e00099-20 (2020). 10.1128/MCB.00099-20 PMC7296212 32284348

[204] Lu MC, Ji JA, Jiang ZY, You QD (2016). The Keap1-Nrf2-ARE pathway as a potential preventive and therapeutic target: an update. Medicinal Res. Rev..

[205] Tang YC (2021). c-MYC-directed NRF2 drives malignant progression of head and neck cancer via glucose-6-phosphate dehydrogenase and transketolase activation. Theranostics.

[206] Chen W (2012). Does Nrf2 contribute to p53-mediated control of cell survival and death?. Antioxid. redox Signal..

[207] Shin D, Kim EH, Lee J, Roh JL (2018). Nrf2 inhibition reverses resistance to GPX4 inhibitor-induced ferroptosis in head and neck cancer. Free Radic. Biol. Med..

[208] Ramesh PS, Devegowda D, Singh A, Thimmulappa RK (2020). NRF2, p53, and p16: Predictive biomarkers to stratify human papillomavirus associated head and neck cancer patients for de-escalation of cancer therapy. Crit. Rev. Oncol./Hematol..

[209] Faraji, F. et al. Genomic hippo pathway alterations and persistent YAP/TAZ activation: new hallmarks in head and neck cancer. *Cells* **11**, 1370 (2022). 10.3390/cells11081370 PMC9028246 35456049

[210] Shin E, Kim J (2020). The potential role of YAP in head and neck squamous cell carcinoma. Exp. Mol. Med..

[211] Segrelles C, Paramio JM, Lorz C (2018). The transcriptional co-activator YAP: a new player in head and neck cancer. Oral. Oncol..

[212] Santos-de-Frutos, K., Segrelles, C. & Lorz, C. Hippo pathway and YAP signaling alterations in squamous cancer of the head and neck. *J. Clin. Med.* **8**, 2131(2019). 10.3390/jcm8122131 PMC6947155 31817001

[213] Hasegawa K (2021). YAP signaling induces PIEZO1 to promote oral squamous cell carcinoma cell proliferation. J. Pathol..

[214] Richtig G (2019). Hedgehog pathway proteins SMO and GLI expression as prognostic markers in head and neck squamous cell carcinoma. Histopathology.

[215] Noman ASM (2020). Widespread expression of Sonic hedgehog (Shh) and Nrf2 in patients treated with cisplatin predicts outcome in resected tumors and are potential therapeutic targets for HPV-negative head and neck cancer. Therapeutic Adv. Med. Oncol..

[216] Enzenhofer E (2016). Impact of sonic hedgehog pathway expression on outcome in HPV negative head and neck carcinoma patients after surgery and adjuvant radiotherapy. PLoS ONE.

[217] Keysar SB (2013). Hedgehog signaling alters reliance on EGF receptor signaling and mediates anti-EGFR therapeutic resistance in head and neck cancer. Cancer Res..

[218] Gan GN (2014). Hedgehog signaling drives radioresistance and stroma-driven tumor repopulation in head and neck squamous cancers. Cancer Res..

[219] Hasnat, S. et al. The prognostic value of toll-like receptors in head and neck squamous cell carcinoma: a systematic review and meta-analysis. *Int. J. Mol. Sci.* **21**, 7255 (2020). 10.3390/ijms21197255 PMC7582583 33008143

[220] Szczepanski MJ (2009). Triggering of Toll-like receptor 4 expressed on human head and neck squamous cell carcinoma promotes tumor development and protects the tumor from immune attack. Cancer Res..

[221] Szczepański M (2007). Assessment of expression of toll-like receptors 2, 3 and 4 in laryngeal carcinoma. Eur. Arch. Oto-Rhino-Laryngol..

[222] Umemura N (2012). Defective NF-κB signaling in metastatic head and neck cancer cells leads to enhanced apoptosis by double-stranded RNA. Cancer Res..

[223] Rydberg C (2009). Toll-like receptor agonists induce inflammation and cell death in a model of head and neck squamous cell carcinomas. Immunology.

[224] Tinhofer I, Braunholz D, Klinghammer K (2020). Preclinical models of head and neck squamous cell carcinoma for a basic understanding of cancer biology and its translation into efficient therapies. Cancers Head Neck.

[225] Kim S (2009). Animal models of cancer in the head and neck region. Clin. Exp. Otorhinolaryngol..

[226] Zeng M (2022). Patient-derived xenograft: a more standard “avatar” model in preclinical studies of gastric cancer. Front Oncol..

[227] Kobayashi, K. et al. A review of HPV-related head and neck cancer. *J. Clin. Med.* **7**, 241 (2018). 10.3390/jcm7090241 PMC6162868 30150513

[228] Hennessey PT, Westra WH, Califano JA (2009). Human papillomavirus and head and neck squamous cell carcinoma: recent evidence and clinical implications. J. Dent. Res..

[229] Ballard DH, Boyer CJ, Alexander JS (2019). Organoids—preclinical models of human disease. N. Engl. J. Med..

[230] Drost J, Clevers H (2018). Organoids in cancer research. Nat. Rev. Cancer.

[231] He J (2020). Organoid technology for tissue engineering. J. Mol. Cell Biol..

[232] Wang XW (2022). Establishment of a patient-derived organoid model and living biobank for nasopharyngeal carcinoma. Ann. Transl. Med..

[233] Sachs N (2018). A living biobank of breast cancer organoids captures disease heterogeneity. Cell.

[234] Dorn CR, Priester WA (1976). Epidemiologic analysis of oral and pharyngeal cancer in dogs, cats, horses, and cattle. J. Am. Vet. Med Assoc..

[235] Lin MC (2017). Therapeutic vaccine targeting Epstein-Barr virus latent protein, LMP1, suppresses LMP1-expressing tumor growth and metastasis in vivo. BMC Cancer.

[236] Yip YL (2018). Establishment of a nasopharyngeal carcinoma cell line capable of undergoing lytic Epstein-Barr virus reactivation. Lab. Investig..

[237] Cai L (2015). Epstein-Barr virus-encoded microRNA BART1 induces tumour metastasis by regulating PTEN-dependent pathways in nasopharyngeal carcinoma. Nat. Commun..

[238] Wei J (2015). Blockage of LMP1-modulated store-operated Ca(2+) entry reduces metastatic potential in nasopharyngeal carcinoma cell. Cancer Lett..

[239] de Jong M, Essers J, van Weerden WM (2014). Imaging preclinical tumour models: improving translational power. Nat. Rev. Cancer.

[240] Hoffman RM (2015). Patient-derived orthotopic xenografts: better mimic of metastasis than subcutaneous xenografts. Nat. Rev. Cancer.

[241] Richmond A, Su Y (2008). Mouse xenograft models vs GEM models for human cancer therapeutics. Dis. Model Mech..

[242] Merlino G (2013). Meeting report: The future of preclinical mouse models in melanoma treatment is now. Pigment Cell Melanoma Res..

[243] Busson P (1988). Establishment and characterization of three transplantable EBV-containing nasopharyngeal carcinomas. Int. J. Cancer.

[244] Garrido-Laguna I (2011). Tumor engraftment in nude mice and enrichment in stroma-related gene pathways predict poor survival and resistance to gemcitabine in patients with pancreatic cancer. Clin. Cancer Res.

[245] Gray DR (2004). Short-term human prostate primary xenografts: an in vivo model of human prostate cancer vasculature and angiogenesis. Cancer Res..

[246] Pitts TM (2010). Development of an integrated genomic classifier for a novel agent in colorectal cancer: approach to individualized therapy in early development. Clin. Cancer Res..

[247] Sanz L (2009). Differential transplantability of human endothelial cells in colorectal cancer and renal cell carcinoma primary xenografts. Lab. Investig..

[248] Smith V, Wirth GJ, Fiebig HH, Burger AM (2008). Tissue microarrays of human tumor xenografts: characterization of proteins involved in migration and angiogenesis for applications in the development of targeted anticancer agents. Cancer Genomics Proteom..

[249] Kono SA, Haigentz M, Yom SS, Saba N (2012). EGFR monoclonal antibodies in the treatment of squamous cell carcinoma of the head and neck: a view beyond cetuximab. Chemother. Res. Pr..

[250] Astsaturov I, Cohen RB, Harari PM (2006). EGFR-targeting monoclonal antibodies in head and neck cancer. Curr. Cancer Drug Targets.

[251] Kim YP (2014). Effective therapeutic approach for head and neck cancer by an engineered minibody targeting the EGFR receptor. PLoS ONE.

[252] Alsahafi EN (2021). EGFR overexpression increases radiotherapy response in HPV-positive head and neck cancer through inhibition of DNA damage repair and HPV E6 downregulation. Cancer Lett..

[253] Huang SM, Harari PM (2000). Modulation of radiation response after epidermal growth factor receptor blockade in squamous cell carcinomas: inhibition of damage repair, cell cycle kinetics, and tumor angiogenesis. Clin. Cancer Res..

[254] Harari PM, Huang SM (2001). Head and neck cancer as a clinical model for molecular targeting of therapy: combining EGFR blockade with radiation. Int. J. Radiat. Oncol. Biol. Phys..

[255] Bouhaddou M (2021). Caveolin-1 and Sox-2 are predictive biomarkers of cetuximab response in head and neck cancer. JCI Insight.

[256] Huang S (2004). Dual-agent molecular targeting of the epidermal growth factor receptor (EGFR): combining anti-EGFR antibody with tyrosine kinase inhibitor. Cancer Res..

[257] Pollack VA (1999). Inhibition of epidermal growth factor receptor-associated tyrosine phosphorylation in human carcinomas with CP-358,774: dynamics of receptor inhibition in situ and antitumor effects in athymic mice. J. Pharm. Exp. Ther..

[258] Sano D (2016). [Corrigendum] Antitumor effects of ZD6474 on head and neck squamous cell carcinoma. Oncol. Rep..

[259] Choi S (2008). Vandetanib inhibits growth of adenoid cystic carcinoma in an orthotopic nude mouse model. Clin. Cancer Res..

[260] Bruzzese F (2006). Synergistic antitumor activity of epidermal growth factor receptor tyrosine kinase inhibitor gefitinib and IFN-alpha in head and neck cancer cells in vitro and in vivo. Clin. Cancer Res..

[261] Bozec A (2008). Combined effects of bevacizumab with erlotinib and irradiation: a preclinical study on a head and neck cancer orthotopic model. Br. J. Cancer.

[262] Lin C (2020). Nerve growth factor (NGF)-TrkA axis in head and neck squamous cell carcinoma triggers EMT and confers resistance to the EGFR inhibitor erlotinib. Cancer Lett..

[263] Raoof S (2019). Targeting FGFR overcomes EMT-mediated resistance in EGFR mutant non-small cell lung cancer. Oncogene.

[264] Wilson GD (2021). Dacomitinib and gedatolisib in combination with fractionated radiation in head and neck cancer. Clin. Transl. Radiat. Oncol..

[265] Torres MA (2011). AC480, formerly BMS-599626, a pan Her inhibitor, enhances radiosensitivity and radioresponse of head and neck squamous cell carcinoma cells in vitro and in vivo. Invest N. Drugs.

[266] Kong, D. H. et al. A review of anti-angiogenic targets for monoclonal antibody cancer therapy. *Int. J. Mol. Sci.* **18**, 1786 (2017). 10.3390/ijms18081786 PMC5578174 28817103

[267] Meadows, K. L. & Hurwitz, H. I. Anti-VEGF therapies in the clinic. *Cold Spring Harb Perspect. Med*. **2**, a006577 (2012). 10.1101/cshperspect.a006577 PMC3475399 23028128

[268] Huang P (2015). RNA interference targeting CD147 inhibits the proliferation, invasiveness, and metastatic activity of thyroid carcinoma cells by down-regulating glycolysis. Int. J. Clin. Exp. Pathol..

[269] Pinheiro C (2015). Reprogramming energy metabolism and inducing angiogenesis: co-expression of monocarboxylate transporters with VEGF family members in cervical adenocarcinomas. BMC Cancer.

[270] Li S, Nguyen TT, Bonanno JA (2014). CD147 required for corneal endothelial lactate transport. Invest. Ophthalmol. Vis. Sci..

[271] Grass GD, Toole BP (2015). How, with whom and when: an overview of CD147-mediated regulatory networks influencing matrix metalloproteinase activity. Biosci. Rep..

[272] Newman JR (2009). EMMPRIN expression is required for response to bevacizumab therapy in HNSCC xenografts. Cancer Lett..

[273] Fujita K (2007). Anti-tumor effects of bevacizumab in combination with paclitaxel on head and neck squamous cell carcinoma. Oncol. Rep..

[274] Wang Y (2010). Investigation of the efficacy of a bevacizumab-cetuximab-cisplatin regimen in treating head and neck squamous cell carcinoma in mice. Target Oncol..

[275] Prichard CN (2007). Concurrent cetuximab and bevacizumab therapy in a murine orthotopic model of anaplastic thyroid carcinoma. Laryngoscope.

[276] Salaun PY (2010). Toxicity and efficacy of combined radioimmunotherapy and bevacizumab in a mouse model of medullary thyroid carcinoma. Cancer.

[277] Sun Q (2022). Lenvatinib for effectively treating antiangiogenic drug-resistant nasopharyngeal carcinoma. Cell Death Dis..

[278] Yamamoto Y (2014). Lenvatinib, an angiogenesis inhibitor targeting VEGFR/FGFR, shows broad antitumor activity in human tumor xenograft models associated with microvessel density and pericyte coverage. Vasc. Cell.

[279] Yigitbasi OG (2004). Tumor cell and endothelial cell therapy of oral cancer by dual tyrosine kinase receptor blockade. Cancer Res..

[280] Younes MN (2006). Concomitant inhibition of epidermal growth factor and vascular endothelial growth factor receptor tyrosine kinases reduces growth and metastasis of human salivary adenoid cystic carcinoma in an orthotopic nude mouse model. Mol. Cancer Ther..

[281] Hu Y, Zhou N, Wang R (2021). Apatinib strengthens the anti-tumor effect of cisplatin in thyroid carcinoma through downregulating VEGFR2. J. BUON.

[282] Chi Y (2022). Apatinib inhibits tumour progression and promotes antitumour efficacy of cytotoxic drugs in oesophageal squamous cell carcinoma. J. Cell Mol. Med..

[283] Liu S (2020). Apatinib combined with radiotherapy enhances antitumor effects in an in vivo nasopharyngeal carcinoma model. Cancer Control.

[284] Zhao TC (2021). Targeting ERK combined with apatinib may be a promising therapeutic strategy for treating oral squamous cell carcinoma. Am. J. Cancer Res..

[285] Bagheri-Yarmand R (2021). ONC201 shows potent anticancer activity against medullary thyroid cancer via transcriptional inhibition of RET, VEGFR2, and IGFBP2. Mol. Cancer Ther..

[286] Lin H (2016). 2ME2 inhibits the activated hypoxia-inducible pathways by cabozantinib and enhances its efficacy against medullary thyroid carcinoma. Tumour Biol..

[287] Hoy SM (2014). Cabozantinib: a review of its use in patients with medullary thyroid cancer. Drugs.

[288] Hsu HW (2013). Linifanib (ABT-869) enhances radiosensitivity of head and neck squamous cell carcinoma cells. Oral. Oncol..

[289] Inteeworn N (2007). Simultaneous application of the vascular endothelial growth factor (VEGF) receptor inhibitor PTK787/ZK 222584 and ionizing radiation does not further reduce the growth of canine oral melanoma xenografts in nude mice. Vet. J..

[290] Goke F (2015). FGFR1 expression levels predict BGJ398 sensitivity of FGFR1-dependent head and neck squamous cell cancers. Clin. Cancer Res..

[291] Sweeny L (2012). Inhibition of fibroblasts reduced head and neck cancer growth by targeting fibroblast growth factor receptor. Laryngoscope.

[292] Fisher MM (2020). Fibroblast growth factor receptors as targets for radiosensitization in head and neck squamous cell carcinomas. Int. J. Radiat. Oncol. Biol. Phys..

[293] Ganci F (2020). PI3K inhibitors curtail MYC-dependent mutant p53 gain-of-function in head and neck squamous cell carcinoma. Clin. Cancer Res..

[294] Meister KS (2019). HER3 targeting potentiates growth suppressive effects of the PI3K inhibitor BYL719 in pre-clinical models of head and neck squamous cell carcinoma. Sci. Rep..

[295] Elkabets M (2015). AXL mediates resistance to PI3Kalpha inhibition by activating the EGFR/PKC/mTOR axis in head and neck and esophageal squamous cell carcinomas. Cancer Cell.

[296] Badarni M (2019). Repression of AXL expression by AP-1/JNK blockage overcomes resistance to PI3Ka therapy. JCI Insight.

[297] Wong CH (2018). Preclinical evaluation of ribociclib and its synergistic effect in combination with alpelisib in non-keratinizing nasopharyngeal carcinoma. Sci. Rep..

[298] Tsuchihashi H (2020). Selective inhibition of PI3K110alpha as a novel therapeutic strategy for cetuximabresistant oral squamous cell carcinoma. Oncol. Rep..

[299] Bhatia S (2018). Role of EphB3 receptor in mediating head and neck tumor growth, cell migration, and response to PI3K inhibitor. Mol. Cancer Ther..

[300] Yun MR (2018). ERK-dependent IL-6 autocrine signaling mediates adaptive resistance to pan-PI3K inhibitor BKM120 in head and neck squamous cell carcinoma. Oncogene.

[301] Bozec A (2017). Combination of phosphotidylinositol-3-kinase targeting with cetuximab and irradiation: a preclinical study on an orthotopic xenograft model of head and neck cancer. Head Neck.

[302] Wong KK, Engelman JA, Cantley LC (2010). Targeting the PI3K signaling pathway in cancer. Curr. Opin. Genet. Dev..

[303] Mishra, R. et al. PI3K inhibitors in cancer: clinical implications and adverse effects. *Int. J. Mol. Sci.* **22**, 3464 (2021). 10.3390/ijms22073464 PMC8037248 33801659

[304] Zumsteg ZS (2016). Taselisib (GDC-0032), a potent beta-sparing small molecule inhibitor of PI3K, radiosensitizes head and neck squamous carcinomas containing activating PIK3CA alterations. Clin. Cancer Res..

[305] Lin J (2010). Inhibitor of differentiation 1 contributes to head and neck squamous cell carcinoma survival via the NF-kappaB/survivin and phosphoinositide 3-kinase/Akt signaling pathways. Clin. Cancer Res..

[306] Fetz V (2009). Inducible NO synthase confers chemoresistance in head and neck cancer by modulating survivin. Int. J. Cancer.

[307] Dong G (2001). Hepatocyte growth factor/scatter factor-induced activation of MEK and PI3K signal pathways contributes to expression of proangiogenic cytokines interleukin-8 and vascular endothelial growth factor in head and neck squamous cell carcinoma. Cancer Res..

[308] Ruicci KM (2020). TAM family receptors in conjunction with MAPK signalling are involved in acquired resistance to PI3Kalpha inhibition in head and neck squamous cell carcinoma. J. Exp. Clin. Cancer Res..

[309] Chiarini F (2008). The novel Akt inhibitor, perifosine, induces caspase-dependent apoptosis and downregulates P-glycoprotein expression in multidrug-resistant human T-acute leukemia cells by a JNK-dependent mechanism. Leukemia.

[310] Chatterjee M (2013). The PI3K/Akt signaling pathway regulates the expression of Hsp70, which critically contributes to Hsp90-chaperone function and tumor cell survival in multiple myeloma. Haematologica.

[311] Fei HR, Chen G, Wang JM, Wang FZ (2010). Perifosine induces cell cycle arrest and apoptosis in human hepatocellular carcinoma cell lines by blockade of Akt phosphorylation. Cytotechnology.

[312] Liu R, Liu D, Xing M (2012). The Akt inhibitor MK2206 synergizes, but perifosine antagonizes, the BRAF(V600E) inhibitor PLX4032 and the MEK1/2 inhibitor AZD6244 in the inhibition of thyroid cancer cells. J. Clin. Endocrinol. Metab..

[313] Lin J (2013). Targeting activated Akt with GDC-0068, a novel selective Akt inhibitor that is efficacious in multiple tumor models. Clin. Cancer Res..

[314] Lang L (2019). Simultaneously inactivating Src and AKT by saracatinib/capivasertib co-delivery nanoparticles to improve the efficacy of anti-Src therapy in head and neck squamous cell carcinoma. J. Hematol. Oncol..

[315] Coppock JD (2013). Improved clearance during treatment of HPV-positive head and neck cancer through mTOR inhibition. Neoplasia.

[316] Bozec A (2016). Combination of mTOR and EGFR targeting in an orthotopic xenograft model of head and neck cancer. Laryngoscope.

[317] Alam MM (2022). Rapalogs induce non-apoptotic, autophagy-dependent cell death in HPV-negative TP53 mutant head and neck squamous cell carcinoma. Mol. Carcinogenesis.

[318] Xie J, Li Q, Ding X, Gao Y (2018). Targeting mTOR by CZ415 inhibits head and neck squamous cell carcinoma cells. Cell Physiol. Biochem..

[319] Cassell A (2012). Targeting TORC1/2 enhances sensitivity to EGFR inhibitors in head and neck cancer preclinical models. Neoplasia.

[320] Wang JY, Jin X, Zhang X, Li XF (2018). CC-223 inhibits human head and neck squamous cell carcinoma cell growth. Biochem Biophys. Res. Commun..

[321] Leiker AJ (2015). Radiation enhancement of head and neck squamous cell carcinoma by the dual PI3K/mTOR inhibitor PF-05212384. Clin. Cancer Res..

[322] Tonlaar N (2017). Antitumor activity of the dual PI3K/MTOR inhibitor, PF-04691502, in combination with radiation in head and neck cancer. Radiother. Oncol..

[323] Mohan S (2015). MEK inhibitor PD-0325901 overcomes resistance to PI3K/mTOR inhibitor PF-5212384 and potentiates antitumor effects in human head and neck squamous cell carcinoma. Clin. Cancer Res..

[324] Erlich RB (2012). Preclinical evaluation of dual PI3K-mTOR inhibitors and histone deacetylase inhibitors in head and neck squamous cell carcinoma. Br. J. Cancer.

[325] Chang KY (2011). Novel phosphoinositide 3-kinase/mTOR dual inhibitor, NVP-BGT226, displays potent growth-inhibitory activity against human head and neck cancer cells in vitro and in vivo. Clin. Cancer Res..

[326] Jiang C (2021). Radiosensitizing effect of c-Met kinase inhibitor BPI-9016M in esophageal squamous cell carcinoma cells in vitro and in vivo. Ann. Transl. Med..

[327] Zhao Y (2015). Met tyrosine kinase inhibitor, PF-2341066, suppresses growth and invasion of nasopharyngeal carcinoma. Drug Des. Devel Ther..

[328] Xu B, Muramatsu T, Inazawa J (2021). Suppression of MET signaling mediated by pitavastatin and capmatinib inhibits oral and esophageal cancer cell growth. Mol. Cancer Res.: MCR.

[329] Vigoda M (2022). Functional proteomics of patient derived head and neck squamous cell carcinoma cells reveal novel applications of trametinib. Cancer Biol. Ther..

[330] Gymnopoulos M (2020). TR1801-ADC: a highly potent cMet antibody-drug conjugate with high activity in patient-derived xenograft models of solid tumors. Mol. Oncol..

[331] Sun S (2014). Targeting the c-Met/FZD8 signaling axis eliminates patient-derived cancer stem-like cells in head and neck squamous carcinomas. Cancer Res..

[332] Bu R (2012). c-Met inhibitor synergizes with tumor necrosis factor-related apoptosis-induced ligand to induce papillary thyroid carcinoma cell death. Mol. Med..

[333] Wei WJ (2017). Obatoclax and LY3009120 efficiently overcome vemurafenib resistance in differentiated thyroid cancer. Theranostics.

[334] Tsumagari K (2018). Bortezomib sensitizes thyroid cancer to BRAF inhibitor in vitro and in vivo. Endocr. Relat. Cancer.

[335] Nucera C (2011). Targeting BRAFV600E with PLX4720 displays potent antimigratory and anti-invasive activity in preclinical models of human thyroid cancer. Oncologist.

[336] Nehs MA (2012). Late intervention with anti-BRAF(V600E) therapy induces tumor regression in an orthotopic mouse model of human anaplastic thyroid cancer. Endocrinology.

[337] Ghosh C (2020). A combinatorial strategy for targeting BRAF (V600E)-mutant cancers with BRAF(V600E) inhibitor (PLX4720) and tyrosine kinase inhibitor (ponatinib). Clin. Cancer Res..

[338] Knauf JA (2018). Hgf/Met activation mediates resistance to BRAF inhibition in murine anaplastic thyroid cancers. J. Clin. Invest..

[339] Robb R (2019). Inhibiting BRAF oncogene-mediated radioresistance effectively radiosensitizes BRAF(V600E)-mutant thyroid cancer cells by constraining DNA double-strand break repair. Clin. Cancer Res..

[340] Jin N (2011). Synergistic action of a RAF inhibitor and a dual PI3K/mTOR inhibitor in thyroid cancer. Clin. Cancer Res..

[341] Song H (2018). The MEK1/2 inhibitor AZD6244 sensitizes BRAF-mutant thyroid cancer to vemurafenib. Med. Sci. Monit..

[342] Affolter A (2016). Targeting irradiation-induced mitogen-activated protein kinase activation in vitro and in an ex vivo model for human head and neck cancer. Head Neck.

[343] Lin X (2019). Inhibition of cisplatin-resistant head and neck squamous cell carcinoma by combination of Afatinib with PD0325901, a MEK inhibitor. Am. J. Cancer Res..

[344] Gong X, Fan L, Wang P (2021). MEK inhibition by trametinib overcomes chemoresistance in preclinical nasopharyngeal carcinoma models. Anticancer Drugs.

[345] Zhi J (2022). Targeting SHP2 sensitizes differentiated thyroid carcinoma to the MEK inhibitor. Am. J. Cancer Res..

[346] Untch BR (2018). Tipifarnib inhibits HRAS-driven dedifferentiated thyroid cancers. Cancer Res..

[347] Pairawan S (2022). Combined MEK/MDM2 inhibition demonstrates antitumor efficacy in TP53 wild-type thyroid and colorectal cancers with MAPK alterations. Sci. Rep..

[348] Enomoto, K. et al. Synergistic effects of lenvatinib (E7080) and MEK inhibitors against anaplastic thyroid cancer in preclinical models. *Cancers* **13**, 862 (2021). 10.3390/cancers13040862 PMC7922355 33670725

[349] Ban MJ (2018). Fibroblast growth factor receptor 3-mediated reactivation of ERK signaling promotes head and neck squamous cancer cell insensitivity to MEK inhibition. Cancer Sci..

[350] Zaballos MA (2022). Inhibiting ERK dimerization ameliorates BRAF-driven anaplastic thyroid cancer. Cell. Mol. Life Sci.: CMLS.

[351] Ferrarotto R (2022). AL101, a gamma-secretase inhibitor, has potent antitumor activity against adenoid cystic carcinoma with activated NOTCH signaling. Cell Death Dis..

[352] Fang J (2015). JAK2 inhibitor blocks the inflammation and growth of esophageal squamous cell carcinoma in vitro through the JAK/STAT3 pathway. Oncol. Rep..

[353] Liu JF (2018). Inhibition of JAK2/STAT3 reduces tumor-induced angiogenesis and myeloid-derived suppressor cells in head and neck cancer. Mol. Carcinogenesis.

[354] Jiang X (2020). CCL18-NIR1 promotes oral cancer cell growth and metastasis by activating the JAK2/STAT3 signaling pathway. BMC Cancer.

[355] Hua Y (2020). NVP-BSK805, an inhibitor of JAK2 kinase, significantly enhances the radiosensitivity of esophageal squamous cell carcinoma in vitro and in vivo. Drug Des. Devel Ther..

[356] Li Z (2018). Loss of the FAT1 tumor suppressor promotes resistance to CDK4/6 inhibitors via the hippo pathway. Cancer Cell.

[357] Adkins D, Ley J, Cohen J, Oppelt P (2022). The potential for selective cyclin-dependent kinase 4/6 inhibition in the therapy for head and neck squamous cell carcinoma. Cancer J..

[358] Burtness B (2022). Integrating targeted therapies in the management of head and neck cancers. Cancer J..

[359] van Caloen, G. et al. Preclinical evaluation of the association of the cyclin-dependent kinase 4/6 inhibitor, ribociclib, and cetuximab in squamous cell carcinoma of the head and neck. *Cancers* **13**, 1251 (2021). 10.3390/cancers13061251 PMC7998503 33809148

[360] Cho J, Johnson DE, Grandis JR (2018). Therapeutic implications of the genetic landscape of head and neck cancer. Semin. Radiat. Oncol..

[361] Ku BM (2016). The CDK4/6 inhibitor LY2835219 has potent activity in combination with mTOR inhibitor in head and neck squamous cell carcinoma. Oncotarget.

[362] Chen L, Pan J (2017). Dual cyclin-dependent kinase 4/6 inhibition by PD-0332991 induces apoptosis and senescence in oesophageal squamous cell carcinoma cells. Br. J. Pharm..

[363] Qin WJ (2022). CDK4/6 inhibitor enhances the radiosensitization of esophageal squamous cell carcinoma (ESCC) by activating autophagy signaling via the suppression of mTOR. Am. J. Transl. Res..

[364] Lan T (2021). Patient-derived xenograft: a developing tool for screening biomarkers and potential therapeutic targets for human esophageal cancers. Aging.

[365] Wang J (2017). CDK4/6 inhibitor-SHR6390 exerts potent antitumor activity in esophageal squamous cell carcinoma by inhibiting phosphorylated Rb and inducing G1 cell cycle arrest. J. Transl. Med..

[366] Lee HJ (2018). A selective cyclin-dependent kinase 4, 6 dual inhibitor, Ribociclib (LEE011) inhibits cell proliferation and induces apoptosis in aggressive thyroid cancer. Cancer Lett..

[367] Li F (2019). YAP1-mediated CDK6 activation confers radiation resistance in esophageal cancer—rationale for the combination of YAP1 and CDK4/6 inhibitors in esophageal cancer. Clin. Cancer Res..

[368] van Caloen G (2020). Preclinical activity of ribociclib in squamous cell carcinoma of the head and neck. Mol. Cancer Ther..

[369] Hsu CL (2018). Integrated genomic analyses in PDX model reveal a cyclin-dependent kinase inhibitor Palbociclib as a novel candidate drug for nasopharyngeal carcinoma. J. Exp. Clin. Cancer Res..

[370] Zainal NS (2019). Effects of palbociclib on oral squamous cell carcinoma and the role of PIK3CA in conferring resistance. Cancer Biol. Med..

[371] Su D (2019). Identification of predictors of drug sensitivity using patient-derived models of esophageal squamous cell carcinoma. Nat. Commun..

[372] Fang Z (2020). MEK blockade overcomes the limited activity of palbociclib in head and neck cancer. Transl. Oncol..

[373] Xue Z (2020). Therapeutic evaluation of palbociclib and its compatibility with other chemotherapies for primary and recurrent nasopharyngeal carcinoma. J. Exp. Clin. Cancer Res..

[374] Hu Q (2020). Metformin as a senostatic drug enhances the anticancer efficacy of CDK4/6 inhibitor in head and neck squamous cell carcinoma. Cell Death Dis..

[375] Petrangolini G (2006). Apoptotic cell death induction and angiogenesis inhibition in large established medullary thyroid carcinoma xenografts by Ret inhibitor RPI-1. Biochem Pharm..

[376] Cuccuru G (2004). Cellular effects and antitumor activity of RET inhibitor RPI-1 on MEN2A-associated medullary thyroid carcinoma. J. Natl Cancer Inst..

[377] Akeno-Stuart N (2007). The RET kinase inhibitor NVP-AST487 blocks growth and calcitonin gene expression through distinct mechanisms in medullary thyroid cancer cells. Cancer Res..

[378] Samadi AK (2010). A novel RET inhibitor with potent efficacy against medullary thyroid cancer in vivo. Surgery.

[379] Bentzien F (2013). In vitro and in vivo activity of cabozantinib (XL184), an inhibitor of RET, MET, and VEGFR2, in a model of medullary thyroid cancer. Thyroid.

[380] Herbst RS (2007). Vandetanib (ZD6474): an orally available receptor tyrosine kinase inhibitor that selectively targets pathways critical for tumor growth and angiogenesis. Expert Opin. Investig. Drugs.

[381] Kim WG (2012). SKI-606, an Src inhibitor, reduces tumor growth, invasion, and distant metastasis in a mouse model of thyroid cancer. Clin. Cancer Res..

[382] Segrelles, C. et al. Bosutinib inhibits EGFR activation in head and neck cancer. *Int. J. Mol. Sci.* **19**, 1824 (2018). 10.3390/ijms19071824 PMC6073167 29933569

[383] Luo F (2021). Gemcitabine and APG-1252, a novel small molecule inhibitor of BCL-2/BCL-XL, display a synergistic antitumor effect in nasopharyngeal carcinoma through the JAK-2/STAT3/MCL-1 signaling pathway. Cell Death Dis..

[384] Fasano M (2021). Head and neck cancer: the role of anti-EGFR agents in the era of immunotherapy. Therapeutic Adv. Med. Oncol..

[385] Bonner JA (2010). Radiotherapy plus cetuximab for locoregionally advanced head and neck cancer: 5-year survival data from a phase 3 randomised trial, and relation between cetuximab-induced rash and survival. Lancet Oncol..

[386] Magrini SM (2016). Cetuximab and radiotherapy versus cisplatin and radiotherapy for locally advanced head and neck cancer: a randomized phase II trial. J. Clin. Oncol..

[387] Maddalo M (2020). Cetuximab and radiation therapy versus cisplatin and radiation therapy for locally advanced head and neck cancer: long-term survival and toxicity outcomes of a randomized phase 2 trial. Int J. Radiat. Oncol. Biol. Phys..

[388] Gebre-Medhin M (2021). ARTSCAN III: a randomized phase III study comparing chemoradiotherapy with cisplatin versus cetuximab in patients with locoregionally advanced head and neck squamous cell cancer. J. Clin. Oncol..

[389] Mehanna H (2019). Radiotherapy plus cisplatin or cetuximab in low-risk human papillomavirus-positive oropharyngeal cancer (De-ESCALaTE HPV): an open-label randomised controlled phase 3 trial. Lancet.

[390] Gillison ML (2019). Radiotherapy plus cetuximab or cisplatin in human papillomavirus-positive oropharyngeal cancer (NRG Oncology RTOG 1016): a randomised, multicentre, non-inferiority trial. Lancet.

[391] Kuhnt T (2017). Hyperfractionated accelerated radiation therapy plus cetuximab plus cisplatin chemotherapy in locally advanced inoperable squamous cell carcinoma of the head and neck: Final 5‑year results of a phase II study. Strahlenther. Onkol..

[392] Ang KK (2014). Randomized phase III trial of concurrent accelerated radiation plus cisplatin with or without cetuximab for stage III to IV head and neck carcinoma: RTOG 0522. J. Clin. Oncol..

[393] Posner MR (2007). Cisplatin and fluorouracil alone or with docetaxel in head and neck cancer. N. Engl. J. Med..

[394] Vermorken JB (2007). Cisplatin, fluorouracil, and docetaxel in unresectable head and neck cancer. N. Engl. J. Med..

[395] Haddad RI (2019). Weekly paclitaxel, carboplatin, cetuximab, and cetuximab, docetaxel, cisplatin, and fluorouracil, followed by local therapy in previously untreated, locally advanced head and neck squamous cell carcinoma. Ann. Oncol..

[396] Seiwert TY (2016). Final results of a randomized phase 2 trial investigating the addition of cetuximab to induction chemotherapy and accelerated or hyperfractionated chemoradiation for locoregionally advanced head and neck cancer. Int. J. Radiat. Oncol. Biol. Phys..

[397] Dietz A (2018). Induction chemotherapy (IC) followed by radiotherapy (RT) versus cetuximab plus IC and RT in advanced laryngeal/hypopharyngeal cancer resectable only by total laryngectomy-final results of the larynx organ preservation trial DeLOS-II. Ann. Oncol..

[398] Keil F (2021). Docetaxel, cisplatin and 5-FU compared with docetaxel, cisplatin and cetuximab as induction chemotherapy in advanced squamous cell carcinoma of the head and neck: Results of a randomised phase II AGMT trial. Eur. J. Cancer.

[399] Kies MS (2010). Induction chemotherapy and cetuximab for locally advanced squamous cell carcinoma of the head and neck: results from a phase II prospective trial. J. Clin. Oncol..

[400] Wanebo HJ (2014). Induction cetuximab, paclitaxel, and carboplatin followed by chemoradiation with cetuximab, paclitaxel, and carboplatin for stage III/IV head and neck squamous cancer: a phase II ECOG-ACRIN trial (E2303). Ann. Oncol..

[401] Giralt J (2015). Panitumumab plus radiotherapy versus chemoradiotherapy in patients with unresected, locally advanced squamous-cell carcinoma of the head and neck (CONCERT-2): a randomised, controlled, open-label phase 2 trial. Lancet Oncol..

[402] Ringash J (2017). Quality of life and swallowing with standard chemoradiotherapy versus accelerated radiotherapy and panitumumab in locoregionally advanced carcinoma of the head and neck: a phase III randomised trial from the Canadian Cancer Trials Group (HN.6). Eur. J. Cancer.

[403] Mesía R (2015). Chemoradiotherapy with or without panitumumab in patients with unresected, locally advanced squamous-cell carcinoma of the head and neck (CONCERT-1): a randomised, controlled, open-label phase 2 trial. Lancet Oncol..

[404] Rodríguez MO (2010). Nimotuzumab plus radiotherapy for unresectable squamous-cell carcinoma of the head and neck. Cancer Biol. Ther..

[405] Reddy BK (2014). Nimotuzumab provides survival benefit to patients with inoperable advanced squamous cell carcinoma of the head and neck: a randomized, open-label, phase IIb, 5-year study in Indian patients. Oral. Oncol..

[406] Patil VM (2019). A randomized phase 3 trial comparing nimotuzumab plus cisplatin chemoradiotherapy versus cisplatin chemoradiotherapy alone in locally advanced head and neck cancer. Cancer.

[407] Menon N (2021). Quality of life in patients with locally advanced head and neck cancer treated with concurrent chemoradiation with cisplatin and nimotuzumab versus cisplatin alone—additional data from a phase 3 trial. Oral. Oncol..

[408] Machiels JP (2011). Zalutumumab plus best supportive care versus best supportive care alone in patients with recurrent or metastatic squamous-cell carcinoma of the head and neck after failure of platinum-based chemotherapy: an open-label, randomised phase 3 trial. Lancet Oncol..

[409] Brøndum L (2018). Associations between skin rash, treatment outcome, and single nucleotide polymorphisms in head and neck cancer patients receiving the EGFR-inhibitor zalutumumab: results from the DAHANCA 19 trial. Acta Oncol..

[410] Cohen EE (2010). Epidermal growth factor receptor inhibitor gefitinib added to chemoradiotherapy in locally advanced head and neck cancer. J. Clin. Oncol..

[411] Del Campo JM (2011). Effects of lapatinib monotherapy: results of a randomised phase II study in therapy-naive patients with locally advanced squamous cell carcinoma of the head and neck. Br. J. Cancer.

[412] Harrington K (2013). Randomised Phase II study of oral lapatinib combined with chemoradiotherapy in patients with advanced squamous cell carcinoma of the head and neck: rationale for future randomised trials in human papilloma virus-negative disease. Eur. J. Cancer.

[413] Harrington K (2015). Postoperative adjuvant lapatinib and concurrent chemoradiotherapy followed by maintenance lapatinib monotherapy in high-risk patients with resected squamous cell carcinoma of the head and neck: a phase III, randomized, double-blind, placebo-controlled study. J. Clin. Oncol..

[414] Vermorken JB (2007). Open-label, uncontrolled, multicenter phase II study to evaluate the efficacy and toxicity of cetuximab as a single agent in patients with recurrent and/or metastatic squamous cell carcinoma of the head and neck who failed to respond to platinum-based therapy. J. Clin. Oncol..

[415] Burtness B (2005). Phase III randomized trial of cisplatin plus placebo compared with cisplatin plus cetuximab in metastatic/recurrent head and neck cancer: an Eastern Cooperative Oncology Group study. J. Clin. Oncol..

[416] Baselga J (2005). Phase II multicenter study of the antiepidermal growth factor receptor monoclonal antibody cetuximab in combination with platinum-based chemotherapy in patients with platinum-refractory metastatic and/or recurrent squamous cell carcinoma of the head and neck. J. Clin. Oncol..

[417] Herbst RS (2005). Phase II multicenter study of the epidermal growth factor receptor antibody cetuximab and cisplatin for recurrent and refractory squamous cell carcinoma of the head and neck. J. Clin. Oncol..

[418] Hitt R (2012). Phase II study of the combination of cetuximab and weekly paclitaxel in the first-line treatment of patients with recurrent and/or metastatic squamous cell carcinoma of head and neck. Ann. Oncol..

[419] Guigay J (2015). Cetuximab, docetaxel, and cisplatin as first-line treatment in patients with recurrent or metastatic head and neck squamous cell carcinoma: a multicenter, phase II GORTEC study. Ann. Oncol..

[420] Tahara M (2018). Phase II trial of combination treatment with paclitaxel, carboplatin and cetuximab (PCE) as first-line treatment in patients with recurrent and/or metastatic squamous cell carcinoma of the head and neck (CSPOR-HN02). Ann. Oncol..

[421] Bossi P (2017). A randomized, phase 2 study of cetuximab plus cisplatin with or without paclitaxel for the first-line treatment of patients with recurrent and/or metastatic squamous cell carcinoma of the head and neck. Ann. Oncol..

[422] Guigay J (2021). Cetuximab, docetaxel, and cisplatin versus platinum, fluorouracil, and cetuximab as first-line treatment in patients with recurrent or metastatic head and neck squamous-cell carcinoma (GORTEC 2014-01 TPExtreme): a multicentre, open-label, randomised, phase 2 trial. Lancet Oncol..

[423] Adkins D (2019). Palbociclib and cetuximab in platinum-resistant and in cetuximab-resistant human papillomavirus-unrelated head and neck cancer: a multicentre, multigroup, phase 2 trial. Lancet Oncol..

[424] Argiris A (2013). Cetuximab and bevacizumab: preclinical data and phase II trial in recurrent or metastatic squamous cell carcinoma of the head and neck. Ann. Oncol..

[425] Sacco AG (2021). Pembrolizumab plus cetuximab in patients with recurrent or metastatic head and neck squamous cell carcinoma: an open-label, multi-arm, non-randomised, multicentre, phase 2 trial. Lancet Oncol..

[426] Chung CH (2022). Phase II multi-institutional clinical trial result of concurrent cetuximab and nivolumab in recurrent and/or metastatic head and neck squamous cell carcinoma. Clin. Cancer Res..

[427] Vermorken JB (2013). Cisplatin and fluorouracil with or without panitumumab in patients with recurrent or metastatic squamous-cell carcinoma of the head and neck (SPECTRUM): an open-label phase 3 randomised trial. Lancet Oncol..

[428] Del Barco Morillo E (2016). Phase II study of panitumumab and paclitaxel as first-line treatment in recurrent or metastatic head and neck cancer. TTCC-2009-03/VECTITAX study. Oral. Oncol..

[429] Wirth LJ (2016). PARTNER: An open-label, randomized, phase 2 study of docetaxel/cisplatin chemotherapy with or without panitumumab as first-line treatment for recurrent or metastatic squamous cell carcinoma of the head and neck. Oral. Oncol..

[430] Zhao C (2019). Anti-epidermal growth factor receptor (EGFR) monoclonal antibody combined with cisplatin and 5-fluorouracil in patients with metastatic nasopharyngeal carcinoma after radical radiotherapy: a multicentre, open-label, phase II clinical trial. Ann. Oncol..

[431] Chen C (2020). Anti-epidermal growth factor receptor monoclonal antibody plus palliative chemotherapy as a first-line treatment for recurrent or metastatic nasopharyngeal carcinoma. Cancer Med..

[432] Stewart JS (2009). Phase III study of gefitinib compared with intravenous methotrexate for recurrent squamous cell carcinoma of the head and neck [corrected]. J. Clin. Oncol..

[433] Argiris A (2013). Phase III randomized, placebo-controlled trial of docetaxel with or without gefitinib in recurrent or metastatic head and neck cancer: an eastern cooperative oncology group trial. J. Clin. Oncol..

[434] Soulieres D (2004). Multicenter phase II study of erlotinib, an oral epidermal growth factor receptor tyrosine kinase inhibitor, in patients with recurrent or metastatic squamous cell cancer of the head and neck. J. Clin. Oncol..

[435] William WN (2018). Single arm, phase II study of cisplatin, docetaxel, and erlotinib in patients with recurrent and/or metastatic head and neck squamous cell carcinomas. Oncologist.

[436] Siu LL (2007). Phase I/II trial of erlotinib and cisplatin in patients with recurrent or metastatic squamous cell carcinoma of the head and neck: a Princess Margaret Hospital phase II consortium and National Cancer Institute of Canada Clinical Trials Group Study. J. Clin. Oncol..

[437] Machiels JP (2015). Afatinib versus methotrexate as second-line treatment in patients with recurrent or metastatic squamous-cell carcinoma of the head and neck progressing on or after platinum-based therapy (LUX-Head & Neck 1): an open-label, randomised phase 3 trial. Lancet Oncol..

[438] Clement PM (2016). Afatinib versus methotrexate in older patients with second-line recurrent and/or metastatic head and neck squamous cell carcinoma: subgroup analysis of the LUX-head & neck 1 trial. Ann. Oncol..

[439] Cohen EEW (2017). Biomarkers predict enhanced clinical outcomes with afatinib versus methotrexate in patients with second-line recurrent and/or metastatic head and neck cancer. Ann. Oncol..

[440] Guo Y (2019). Afatinib versus methotrexate as second-line treatment in Asian patients with recurrent or metastatic squamous cell carcinoma of the head and neck progressing on or after platinum-based therapy (LUX-head & neck 3): an open-label, randomised phase III trial. Ann. Oncol..

[441] Kao HF (2022). Afatinib and pembrolizumab for recurrent or metastatic head and neck squamous cell carcinoma (ALPHA Study): a phase II study with biomarker analysis. Clin. Cancer Res..

[442] Abdul Razak AR (2013). A phase II trial of dacomitinib, an oral pan-human EGF receptor (HER) inhibitor, as first-line treatment in recurrent and/or metastatic squamous-cell carcinoma of the head and neck. Ann. Oncol..

[443] Kim HS (2015). Phase II clinical and exploratory biomarker study of dacomitinib in patients with recurrent and/or metastatic squamous cell carcinoma of head and neck. Clin. Cancer Res..

[444] Lee JH (2021). A phase II study of poziotinib in patients with recurrent and/or metastatic head and neck squamous cell carcinoma. Cancer Med..

[445] Wilhelm SM (2004). BAY 43-9006 exhibits broad spectrum oral antitumor activity and targets the RAF/MEK/ERK pathway and receptor tyrosine kinases involved in tumor progression and angiogenesis. Cancer Res..

[446] Murphy DA (2006). Inhibition of tumor endothelial ERK activation, angiogenesis, and tumor growth by sorafenib (BAY43-9006). Am. J. Pathol..

[447] Brose MS (2014). Sorafenib in radioactive iodine-refractory, locally advanced or metastatic differentiated thyroid cancer: a randomised, double-blind, phase 3 trial. Lancet.

[448] Brose MS (2019). Analysis of biomarkers and association with clinical outcomes in patients with differentiated thyroid cancer: subanalysis of the sorafenib phase III DECISION trial. Clin. Cancer Res..

[449] Elser C (2007). Phase II trial of sorafenib in patients with recurrent or metastatic squamous cell carcinoma of the head and neck or nasopharyngeal carcinoma. J. Clin. Oncol..

[450] Xue C (2013). Phase II study of sorafenib in combination with cisplatin and 5-fluorouracil to treat recurrent or metastatic nasopharyngeal carcinoma. Ann. Oncol..

[451] Schlumberger M (2015). Lenvatinib versus placebo in radioiodine-refractory thyroid cancer. N. Engl. J. Med..

[452] Brose MS (2017). Effect of age on the efficacy and safety of lenvatinib in radioiodine-refractory differentiated thyroid cancer in the phase III SELECT trial. J. Clin. Oncol..

[453] Higashiyama T (2022). Phase II study of the efficacy and safety of lenvatinib for anaplastic thyroid cancer (HOPE). Eur. J. Cancer.

[454] Wirth LJ (2021). Open-label, single-arm, multicenter, phase II trial of lenvatinib for the treatment of patients with anaplastic thyroid cancer. J. Clin. Oncol..

[455] Petrelli A, Giordano S (2008). From single- to multi-target drugs in cancer therapy: when aspecificity becomes an advantage. Curr. Med. Chem..

[456] Qin S (2019). Recent advances on anti-angiogenesis receptor tyrosine kinase inhibitors in cancer therapy. J. Hematol. Oncol..

[457] Bhullar KS (2018). Kinase-targeted cancer therapies: progress, challenges and future directions. Mol. Cancer.

[458] Duckett DR, Cameron MD (2010). Metabolism considerations for kinase inhibitors in cancer treatment. Expert Opin. Drug Metab. Toxicol..

[459] Petrelli A, Valabrega G (2009). Multitarget drugs: the present and the future of cancer therapy. Expert Opin. Pharmacother..

[460] Machiels JP (2010). Phase II study of sunitinib in recurrent or metastatic squamous cell carcinoma of the head and neck: GORTEC 2006-01. J. Clin. Oncol..

[461] Hui EP (2011). Hemorrhagic complications in a phase II study of sunitinib in patients of nasopharyngeal carcinoma who has previously received high-dose radiation. Ann. Oncol..

[462] Carr LL (2010). Phase II study of daily sunitinib in FDG-PET-positive, iodine-refractory differentiated thyroid cancer and metastatic medullary carcinoma of the thyroid with functional imaging correlation. Clin. Cancer Res..

[463] Ravaud A (2017). A multicenter phase II study of sunitinib in patients with locally advanced or metastatic differentiated, anaplastic or medullary thyroid carcinomas: mature data from the THYSU study. Eur. J. Cancer.

[464] Lin Y (2022). Apatinib vs placebo in patients with locally advanced or metastatic, radioactive iodine-refractory differentiated thyroid cancer: the REALITY randomized clinical trial. JAMA Oncol..

[465] Li L (2020). Apatinib, a novel VEGFR-2 tyrosine kinase inhibitor, for relapsed and refractory nasopharyngeal carcinoma: data from an open-label, single-arm, exploratory study. Invest. N. Drugs.

[466] Ruan X (2021). Apatinib for the treatment of metastatic or locoregionally recurrent nasopharyngeal carcinoma after failure of chemotherapy: a multicenter, single-arm, prospective phase 2 study. Cancer.

[467] Liu X (2022). Adjuvant apatinib in nasopharyngeal carcinoma with residual Epstein-Barr virus DNA after radiation therapy: a biomarker-driven, phase 2 trial. Int. J. Radiat. Oncol. Biol. Phys..

[468] Ju WT (2022). A pilot study of neoadjuvant combination of anti-PD-1 camrelizumab and VEGFR2 inhibitor apatinib for locally advanced resectable oral squamous cell carcinoma. Nat. Commun..

[469] Huang NS (2021). The efficacy and safety of anlotinib in neoadjuvant treatment of locally advanced thyroid cancer: a single-arm phase II clinical trial. Thyroid.

[470] Li D (2021). Anlotinib in locally advanced or metastatic medullary thyroid carcinoma: a randomized, double-blind phase IIB trial. Clin. Cancer Res..

[471] Sun Y (2018). Anlotinib for the treatment of patients with locally advanced or metastatic medullary thyroid cancer. Thyroid.

[472] Lin YS (2021). Donafenib in progressive locally advanced or metastatic radioactive iodine-refractory differentiated thyroid cancer: results of a randomized, multicenter phase II trial. Thyroid.

[473] Argiris A (2019). Phase III randomized trial of chemotherapy with or without bevacizumab in patients with recurrent or metastatic head and neck cancer. J. Clin. Oncol..

[474] Argiris A (2011). Phase II trial of pemetrexed and bevacizumab in patients with recurrent or metastatic head and neck cancer. J. Clin. Oncol..

[475] Lee NY (2012). Addition of bevacizumab to standard chemoradiation for locoregionally advanced nasopharyngeal carcinoma (RTOG 0615): a phase 2 multi-institutional trial. Lancet Oncol..

[476] Salama JK (2011). A randomized phase II study of 5-fluorouracil, hydroxyurea, and twice-daily radiotherapy compared with bevacizumab plus 5-fluorouracil, hydroxyurea, and twice-daily radiotherapy for intermediate-stage and T4N0-1 head and neck cancers. Ann. Oncol..

[477] Ciardiello F (2003). Antitumor effects of ZD6474, a small molecule vascular endothelial growth factor receptor tyrosine kinase inhibitor, with additional activity against epidermal growth factor receptor tyrosine kinase. Clin. Cancer Res..

[478] Cohen EE (2009). Erlotinib and bevacizumab in patients with recurrent or metastatic squamous-cell carcinoma of the head and neck: a phase I/II study. Lancet Oncol..

[479] Yoo DS (2012). Prospective trial of synchronous bevacizumab, erlotinib, and concurrent chemoradiation in locally advanced head and neck cancer. Clin. Cancer Res..

[480] Argiris A (2016). Phase II randomized trial of radiation therapy, cetuximab, and pemetrexed with or without bevacizumab in patients with locally advanced head and neck cancer. Ann. Oncol..

[481] Kim HR (2020). Mouse-human co-clinical trials demonstrate superior anti-tumour effects of buparlisib (BKM120) and cetuximab combination in squamous cell carcinoma of head and neck. Br. J. Cancer.

[482] Brisson RJ (2019). A pilot study of the pan-class I PI3K inhibitor buparlisib in combination with cetuximab in patients with recurrent or metastatic head and neck cancer. Head Neck.

[483] Soulières D (2017). Buparlisib and paclitaxel in patients with platinum-pretreated recurrent or metastatic squamous cell carcinoma of the head and neck (BERIL-1): a randomised, double-blind, placebo-controlled phase 2 trial. Lancet Oncol..

[484] Soulières D (2018). Molecular alterations and buparlisib efficacy in patients with squamous cell carcinoma of the head and neck: biomarker analysis from BERIL-1. Clin. Cancer Res..

[485] Dunn LA (2020). A Phase 1b study of cetuximab and BYL719 (alpelisib) concurrent with intensity modulated radiation therapy in stage III-IVB head and neck squamous cell carcinoma. Int. J. Radiat. Oncol. Biol. Phys..

[486] Day D (2020). Phase I trial of alpelisib in combination with concurrent cisplatin-based chemoradiotherapy in patients with locoregionally advanced squamous cell carcinoma of the head and neck. Oral. Oncol..

[487] Marret G (2021). Phase I trial of copanlisib, a selective PI3K inhibitor, in combination with cetuximab in patients with recurrent and/or metastatic head and neck squamous cell carcinoma. Invest. N. Drugs.

[488] Jimeno A (2015). A randomized, phase II trial of cetuximab with or without PX-866, an irreversible oral phosphatidylinositol 3-kinase inhibitor, in patients with relapsed or metastatic head and neck squamous cell cancer. Ann. Oncol..

[489] Shome D (2008). Ulcerative keratitis in gastrointestinal stromal tumor patients treated with perifosine. Ophthalmology.

[490] Ramanathan RK (2015). Phase 2 study of MK-2206, an allosteric inhibitor of AKT, as second-line therapy for advanced gastric and gastroesophageal junction cancer: a SWOG cooperative group trial (S1005). Cancer.

[491] Oldford SA, Marshall JS (2015). Mast cells as targets for immunotherapy of solid tumors. Mol. Immunol..

[492] Argiris A (2006). A phase II trial of perifosine, an oral alkylphospholipid, in recurrent or metastatic head and neck cancer. Cancer Biol. Ther..

[493] Lim SM (2013). A multicenter, phase II trial of everolimus in locally advanced or metastatic thyroid cancer of all histologic subtypes. Ann. Oncol..

[494] Massarelli E (2015). Phase II trial of everolimus and erlotinib in patients with platinum-resistant recurrent and/or metastatic head and neck squamous cell carcinoma. Ann. Oncol..

[495] Geiger JL (2016). Phase II trial of everolimus in patients with previously treated recurrent or metastatic head and neck squamous cell carcinoma. Head Neck.

[496] Schneider TC (2017). Everolimus in patients with advanced follicular-derived thyroid cancer: results of a phase II clinical trial. J. Clin. Endocrinol. Metab..

[497] Hanna GJ (2018). Genomic correlates of response to everolimus in aggressive radioiodine-refractory thyroid cancer: a phase II study. Clin. Cancer Res..

[498] Grünwald V (2015). TEMHEAD: a single-arm multicentre phase II study of temsirolimus in platin- and cetuximab refractory recurrent and/or metastatic squamous cell carcinoma of the head and neck (SCCHN) of the German SCCHN Group (AIO). Ann. Oncol..

[499] John K (2015). Baseline caspase activity predicts progression free survival of temsirolimus-treated head neck cancer patients. Eur. J. Cancer.

[500] Dunn LA (2017). A phase II study of temsirolimus added to low-dose weekly carboplatin and paclitaxel for patients with recurrent and/or metastatic (R/M) head and neck squamous cell carcinoma (HNSCC). Ann. Oncol..

[501] Seiwert TY (2020). A randomized phase 2 study of temsirolimus and cetuximab versus temsirolimus alone in recurrent/metastatic, cetuximab-resistant head and neck cancer: the MAESTRO study. Cancer.

[502] Shi K (2022). Emerging strategies to overcome resistance to third-generation EGFR inhibitors. J. Hematol. Oncol..

[503] Krajewska J, Olczyk T, Jarzab B (2016). Cabozantinib for the treatment of progressive metastatic medullary thyroid cancer. Expert Rev. Clin. Pharm..

[504] Elisei R (2013). Cabozantinib in progressive medullary thyroid cancer. J. Clin. Oncol..

[505] Schlumberger M (2017). Overall survival analysis of EXAM, a phase III trial of cabozantinib in patients with radiographically progressive medullary thyroid carcinoma. Ann. Oncol..

[506] Kurzrock R (2011). Activity of XL184 (Cabozantinib), an oral tyrosine kinase inhibitor, in patients with medullary thyroid cancer. J. Clin. Oncol..

[507] Brose MS (2021). Cabozantinib for radioiodine-refractory differentiated thyroid cancer (COSMIC-311): a randomised, double-blind, placebo-controlled, phase 3 trial. Lancet Oncol..

[508] Cabanillas ME (2017). Cabozantinib as salvage therapy for patients with tyrosine kinase inhibitor-refractory differentiated thyroid cancer: results of a multicenter phase II International Thyroid Oncology Group Trial. J. Clin. Oncol..

[509] Seiwert T (2013). Phase II trial of single-agent foretinib (GSK1363089) in patients with recurrent or metastatic squamous cell carcinoma of the head and neck. Invest. N. Drugs.

[510] Kochanny SE (2020). A randomized phase 2 network trial of tivantinib plus cetuximab versus cetuximab in patients with recurrent/metastatic head and neck squamous cell carcinoma. Cancer.

[511] Wirth LJ (2020). Efficacy of selpercatinib in RET-altered thyroid cancers. N. Engl. J. Med..

[512] Wells SA (2012). Vandetanib in patients with locally advanced or metastatic medullary thyroid cancer: a randomized, double-blind phase III trial. J. Clin. Oncol..

[513] Subbiah V (2021). Pralsetinib for patients with advanced or metastatic RET-altered thyroid cancer (ARROW): a multi-cohort, open-label, registrational, phase 1/2 study. Lancet Diabetes Endocrinol..

[514] Mitchell TC (2018). Epacadostat plus pembrolizumab in patients with advanced solid tumors: phase I results from a multicenter, open-label phase I/II trial (ECHO-202/KEYNOTE-037). J. Clin. Oncol..

[515] Jimeno A (2016). A phase 2 study of dalantercept, an activin receptor-like kinase-1 ligand trap, in patients with recurrent or metastatic squamous cell carcinoma of the head and neck. Cancer.

[516] Billard-Sandu C (2020). CDK4/6 inhibitors in P16/HPV16-negative squamous cell carcinoma of the head and neck. Eur. Arch. Oto-Rhino-Laryngol..

[517] van Caloen G, Machiels JP (2019). Potential role of cyclin-dependent kinase 4/6 inhibitors in the treatment of squamous cell carcinoma of the head and neck. Curr. Opin. Oncol..

[518] Kalu NN, Johnson FM (2017). Do CDK4/6 inhibitors have potential as targeted therapeutics for squamous cell cancers?. Expert Opin. Investig. Drugs.

[519] Rikiishi H (2012). Autophagic action of new targeting agents in head and neck oncology. Cancer Biol. Ther..

[520] Chong CR, Janne PA (2013). The quest to overcome resistance to EGFR-targeted therapies in cancer. Nat. Med..

[521] Lim SM, Syn NL, Cho BC, Soo RA (2018). Acquired resistance to EGFR targeted therapy in non-small cell lung cancer: mechanisms and therapeutic strategies. Cancer Treat. Rev..

[522] Chen LF, Cohen EE, Grandis JR (2010). New strategies in head and neck cancer: understanding resistance to epidermal growth factor receptor inhibitors. Clin. Cancer Res..

[523] Perisanidis C (2017). Prevalence of EGFR tyrosine kinase domain mutations in head and neck squamous cell carcinoma: cohort study and systematic review. Vivo.

[524] Padma VV (2015). An overview of targeted cancer therapy. Biomedicine.

[525] Stribbling SM (1997). Biodistribution of an antibody-enzyme conjugate for antibody-directed enzyme prodrug therapy in nude mice bearing a human colon adenocarcinoma xenograft. Cancer Chemother. Pharm..

[526] Syrigos KN, Epenetos AA (1999). Antibody directed enzyme prodrug therapy (ADEPT): a review of the experimental and clinical considerations. Anticancer Res..

[527] Syrigos KN, Rowlinson-Busza G, Epenetos AA (1998). In vitro cytotoxicity following specific activation of amygdalin by beta-glucosidase conjugated to a bladder cancer-associated monoclonal antibody. Int. J. Cancer.

